# Elevated mitochondrial protein import in acute myeloid leukemia increases reliance on mitochondrial protease LONP1

**DOI:** 10.1172/JCI196687

**Published:** 2026-06-16

**Authors:** Matthew Tcheng, Veronique Voisin, Geethu Emily Thomas, Anastasija A. Piric, Marcela Gronda, Rose Hurren, Dakai Ling, Yongran Yan, Lan Xin Zhang, Yue Feng, Ali Chegini, Nathan Duong, Ross S. Mancini, Stefan Quinn W. Currie, Zaynab Mamai, Brady Stock, Shahbaz Khan, Yulia Jitkova, Chaitra Sarathy, Edward Ayoub, Po Yee Mak, Andrea Arruda, Thomas Kislinger, Mark A. Reed, Bing Z. Carter, Michael Andreeff, Steven M. Kornblau, Mark D. Minden, Siavash Vahidi, Aaron D. Schimmer

**Affiliations:** 1 Princess Margaret Cancer Centre,; 2 Krembil Brain Institute, University Health Network, Toronto, Ontario, Canada.; 3 Department of Molecular and Cell Biology, University of Guelph, Guelph, Ontario, Canada.; 4 Section of Molecular Hematology and Therapy, Department of Leukemia, The University of Texas MD Anderson Cancer Center, Houston, Texas, USA.; 5 Department of Pharmacology and Toxicology, and; 6 Department of Chemistry, University of Toronto, Toronto, Ontario, Canada.; 7 Ontario Institute for Cancer Research, Toronto, Ontario, Canada.

**Keywords:** Cell biology, Metabolism, Oncology, Cancer

## Abstract

Most mitochondrial proteins are nucleus-encoded, translated in the cytosol, and imported into the mitochondria. Through gene expression analysis and functional assays, we demonstrated that mitochondrial protein import was increased in acute myeloid leukemia (AML) cells compared with normal hematopoietic cells. Increased mitochondrial protein import was positively correlated with an increase in the mitochondrial unfolded protein response (UPR^mt^), a stress-activated pathway of mitochondrial proteases and chaperones that maintains protein solubility and prevents the formation of toxic aggregates. The UPR^mt^ protease LONP1 (Lon peptidase 1) was upregulated in AML and positively correlated with increased mitochondrial protein import and UPR^mt^. Genetic or chemical inhibition of the LONP1 ATPase domain induced mitochondrial protein aggregation and selectively killed AML cells with high LONP1 expression, while sparing AML cells with low LONP1 expression and normal hematopoietic cells in vitro and in vivo. Thus, we uncovered a critical role of the UPR^mt^ protease LONP1 in buffering stress from mitochondrial protein import in AML.

## Abstract 2

Compared to normal hematopoietic cells, AML cells upregulate mitochondrial protein import and are more reliant on the mitochondrial protease LONP1 to maintain mitochondrial protein solubility.

## Graphical abstract

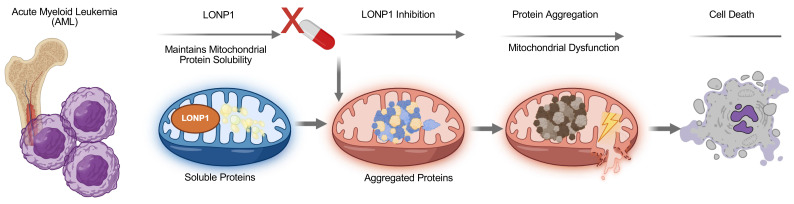

## Introduction

Mitochondria contain approximately 1,100 unique proteins, 99% of which are encoded by nuclear DNA and translated in the cytoplasm (1). From the cytoplasm, these protein precursors are imported into the subcompartments of the mitochondria through a series of protein import channels (2). Once inside the mitochondria, these unfolded, aggregation-prone precursors are processed and folded into their mature forms (2–4). To achieve this, cells employ the mitochondrial unfolded protein response (UPR^mt^) to prevent the formation of toxic protein aggregates and resolve damage to mature mitochondrial proteins (5–8). UPR^mt^ is an evolutionarily conserved pathway where mitochondrial stress is signaled to the nucleus, leading to the upregulation of nucleus-encoded mitochondrial chaperones and proteases to dampen and resolve the stress (5–8). UPR^mt^ is conceptually analogous to the ER UPR, although the specific mediators are distinct (6, 9).

Acute myeloid leukemia (AML) is an aggressive hematologic malignancy (10). Previously, our group and others demonstrated that AML cells and stem cells exhibit a distinct metabolic phenotype characterized by an increased mitochondrial mass (11), greater influx of substrates into the TCA cycle (12–14), reduced spare reserve capacity (11), and a heightened dependence on mitochondrial metabolism (15, 16) for survival and proliferation.

Here, we show that the UPR^mt^ was increased in AML to compensate for greater mitochondrial protein import. We discovered that LONP1, an AAA+ (ATPases associated with diverse cellular activities) unfoldase and serine protease localized to the mitochondrial matrix, acted as a critical mediator of the UPR^mt^ in AML. Through its AAA+ domain, LONP1 also acts in concert with mitochondrial chaperones to fold newly imported proteins. AML cells uniquely rely on LONP1 to maintain mitochondrial protein solubility. Genetic or chemical inhibition of the LONP1 AAA+ domain led to mitochondrial protein aggregation and cell death in AML cells with high LONP1 expression. In contrast, normal cells had lower levels of mitochondrial protein import and UPR^mt^ and were insensitive to LONP1 inhibition. Thus, our work highlights findings related to mitochondrial biology and a promising therapeutic strategy for a subgroup of patients with AML.

## Results

### Mitochondrial protein import is increased in primary AML compared with normal hematopoietic cells.

**Figure 1:**
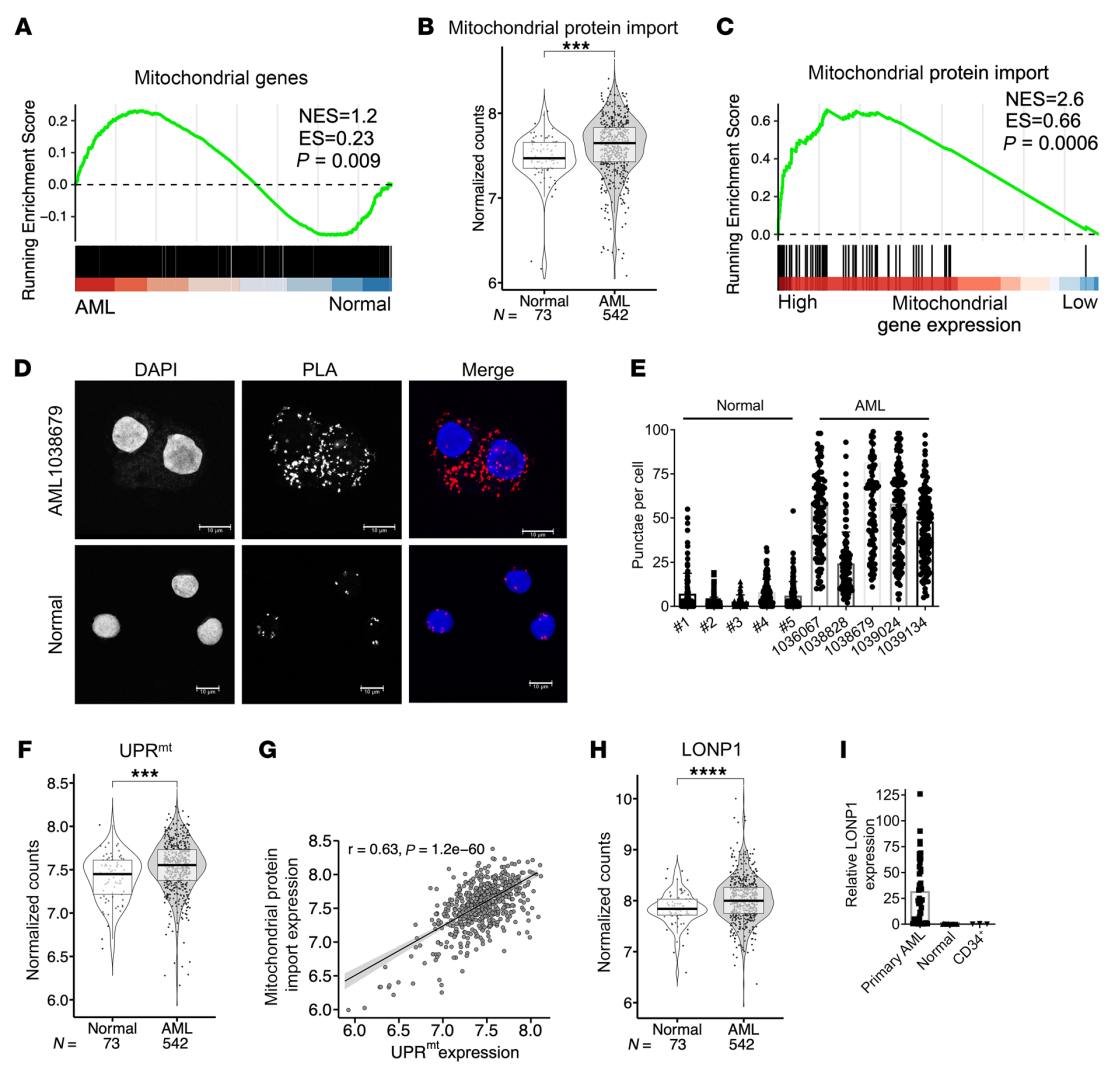
Mitochondrial protein import and the UPR^mt^ are increased in a subset of primary AML, compared with normal hematopoietic cells. (**A**) Enrichment plot of nuclear-encoded mitochondrial gene expression in primary AML (*n* = 542) and normal mononuclear bone marrow hematopoietic cells (*n* = 73) from GSE13159. (**B**) Violin plot of mitochondrial protein import gene expression (GO 0072655) in primary AML and normal mononuclear bone marrow hematopoietic cells from GEO GSE13159. The midline represents the median value. ***P = 4.9 × 10^–4^ using an unpaired, 2-tailed Student’s *t* test. (**C**) Enrichment plot of mitochondrial protein import genes in primary AML from GSE13159, stratified by mitochondrial gene expression. (**D**) Primary AML and normal mononuclear bone marrow hematopoietic cells were treated with puromycin (1 μg/mL) for 7.5 minutes, fixed, stained with anti-puromycin, anti-TOMM40 (mitochondria) antibodies, and DAPI (nucleus). Colocalization of puromycin and TOMM40 was detected by a PLA and confocal microscopy. Representative cells are shown. Scale bars: 10 μm. (**E**) Mean ± SD PLA punctae per cell from **D** in primary AML cell samples (*n* = 5) and normal mononuclear bone marrow hematopoietic cells (*n* = 5), quantified using HALO software (*n* = 82–154 cells per sample). (**F**) Violin plot of UPR^mt^ gene expression in primary AML and normal mononuclear bone marrow hematopoietic cells from GSE13159. ***P = 1.1 × 10^–3^ using an unpaired, 2-tailed Student’s *t* test. (**G**) Correlation analysis of UPR^mt^ and mitochondrial protein import gene expression in primary AML samples from GSE13159. (**H**) Violin plot of *LONP1* mRNA expression in primary AML and normal bone marrow hematopoietic cells from GSE13159. ****P = 6.9 × 10^–5^ using an unpaired, 2-tailed Student’s *t* test. (**I**) Expression of LONP1 protein in primary AML cells (*n* = 39), normal hematopoietic cells (*n* = 14), and CD34^+^ progenitors (*n* = 3) was measured in cell lysates by immunoblotting. Expression was quantified by densitometry and displayed relative to LONP1 protein levels in OCI-AML2 cells. See Supplemental Figure 5B, Supplemental Table 2 (patient cytogenetics). Data are presented as mean ± SD.

Nucleus-encoded proteins destined for the mitochondria are imported into the organelle through a series of protein import channels and carriers (1, 2). We demonstrated that expression of all 1,144 nucleus-encoded mitochondrial genes was enriched in AML cells in a subset of patients with AML compared with normal bone marrow mononuclear cells. (Figure 1A). Genes associated with mitochondrial protein import were also upregulated in a subset of patients with AML (Figure 1B and Supplemental Figure 1A; supplemental material available online with this article; https://doi.org/10.1172/JCI196687DS1). Specifically, 38% of patients with AML had increased expression of genes associated with mitochondrial protein import compared with the 90th percentile expression in normal bone marrow mononuclear cells (Figure 1B). Genes and proteins associated with mitochondrial protein import were equally expressed across ELN2017 risk groups (17), cytogenetic subtypes, and morphologic subtypes of AML (Supplemental Figure 1, B and C). The average expression of mitochondrial protein import genes or proteins was not associated with survival for patients with AML (Supplemental Figure 1, D and E). Higher expression of mitochondrial protein import genes correlated with higher expression of mitochondrial genes (Figure 1C).

Given the upregulation of genes associated with mitochondrial protein import in AML, we developed a proximity ligation assay (PLA) to quantify the import of newly synthesized proteins into the mitochondria. Newly synthesized proteins were labeled with puromycin, and a PLA assay was conducted to measure the interaction between puromycin-tagged proteins and the translocase of the outer mitochondrial membrane 40 (TOMM40) import channel. We validated the assay in AML cell lines (OCI-AML2, OCI-M2, TEX, and NB4), in which mitochondrial protein import and the PLA signal were blocked by treating cells with either cycloheximide to inhibit protein synthesis, or FCCP to reduce mitochondrial membrane potential and MitoBloCK-6 to inhibit the oxidase activity of GFER/Erv1 in the intermembrane space and thereby inhibit mitochondrial protein import similar to previously described (18–20) (Supplemental Figure 2). With this assay, we measured the import of newly synthesized proteins into the mitochondria of primary AML and normal mononuclear hematopoietic cells derived from consenting individuals donating peripheral blood granulocyte colony-stimulating factor (GCSF) mobilized hematopoietic stem and progenitor cells for allotransplantation. Compared with normal hematopoietic cells, mean mitochondrial protein import was 10-fold higher in primary AML cells (Figure 1, D and E).

### UPR^mt^ is upregulated in AML.

Newly imported precursor proteins must be folded into their mature forms by mitochondrial proteases and chaperones to prevent protein aggregation (2, 21). The UPR^mt^ is an evolutionarily conserved pathway that activates the nuclear transcription of mitochondrial proteases and chaperones in response to mitochondrial proteotoxic stress (7, 8, 22), including the stress from newly imported precursor proteins (23–25). Since there are no dedicated human UPR^mt^ gene sets in the Gene Ontology (GO) and Kyoto Encyclopedia of Genes and Genomes (KEGG) databases, we compiled a 39-gene set of the known components of the UPR^mt^ (Supplemental Figure 3A and Supplemental Table 1). Using this gene set, we measured UPR^mt^ expression in primary AML and normal mononuclear bone marrow hematopoietic cell samples. UPR^mt^ expression was higher in a subgroup of patients with AML compared with normal hematopoietic cell samples (Figure 1F and Supplemental Figure 3B). Thirty-two percent of patients with AML had increased UPR^mt^ expression compared with the 90th percentile of normal bone marrow mononuclear hematopoietic cells (Figure 1F). UPR^mt^ expression correlated with expression of the mitochondrial protein import machinery, reflecting the role of the UPR^mt^ in protecting mitochondria from the influx of newly imported proteins (Figure 1G). UPR^mt^ genes and proteins were equally expressed across AML ELN2017 risk groups (17), French-American-British (FAB) classification subtypes, and molecular mutation subtypes (Supplemental Figure 3, C and D). Increased UPR^mt^ gene and protein expression was not associated with differences in survival of patients with AML (Supplemental Figure 3, E and F).

### LONP1 is essential for a subgroup of AML cell lines and patient samples with high LONP1 expression.

To understand the essentiality of the UPR^mt^ in AML, we identified a core set of 18 genes that contributed to the positive enrichment of UPR^mt^ in patients with AML with high levels of protein import (Supplemental Figure 4, A and B). This core set of 18 genes was also enriched and overexpressed in patients with AML, compared with normal adult bone marrow mononuclear hematopoietic samples (Supplemental Figure 4, C–E). We then ranked these 18 leading-edge genes in CRISPR and shRNA gene dependency screens (26). These datasets assign a score expressing a cell line’s dependence on a particular gene for survival. Genes with a score below –0.5 are considered essential (26). Of the 18 genes, 4 genes from the CRISPR screens (*LONP1*, *DNAJA3*, *PMPCB*, *PMPCA*) and 3 genes from the shRNA screens (*LONP1*, *DNAJA3*, *PMPCB*) had mean gene effect scores below –0.5 and were therefore essential to AML cell lines (Supplemental Figure 4F). Of the 18 genes, the mitochondrial protease *LONP1* qualified as a top dependency in CRISPR and shRNA dependency screens and was selected for further analysis (Supplemental Figure 4G). LONP1 is a nucleus-encoded member of the AAA+ motor protein superfamily and is composed of 3 distinct domains: an N-terminal domain, a AAA+ domain, and a protease domain (27). LONP1 maintains mitochondrial proteostasis through progressive degradation of misfolded protein substrates, yet also collaborates with mitochondrial chaperones to regulate the folding of newly imported proteins (27, 28).

*LONP1* mRNA expression was increased in a subgroup of patients with AML (Figure 1H and Supplemental Figure 5A). Specifically, in the GSE13159 and BeatAML2. datasets, LONP1 mRNA expression was increased in 32% and 34% of patients with AML, respectively, compared with the 90th percentile distribution in normal bone marrow mononuclear hematopoietic cells (Figure 1H and Supplemental Figure 5A). Similarly, by immunoblotting, LONP1 protein expression was increased more than 2-fold in 26 of 39 primary AML samples, compared with normal (*n* = 14) and CD34^+^ (*n* = 3) adult hematopoietic cells derived from consenting individuals who donated peripheral blood GCSF mobilized hematopoietic stem and progenitor cells for allotransplantation (Figure 1I and Supplemental Figure 5B). LONP1 mRNA and protein were equally expressed across AML ELN2017 risk groups (17), FAB morphologic subtypes, and molecular mutation subtypes (Supplemental Figure 6). LONP1 was equally expressed in individuals with relapsed versus newly diagnosed AML (Supplemental Figure 7A). Increased LONP1 protein expression was associated with decreased overall survival for patients with AML (Supplemental Figure 7B), but *LONP1* mRNA expression was not correlated with survival (Supplemental Figure 7C). In primary AML samples, *LONP1* expression positively correlated with the expression of mitochondrial protein import and UPR^mt^ genes (Supplemental Figure 8).

**Figure 2:**
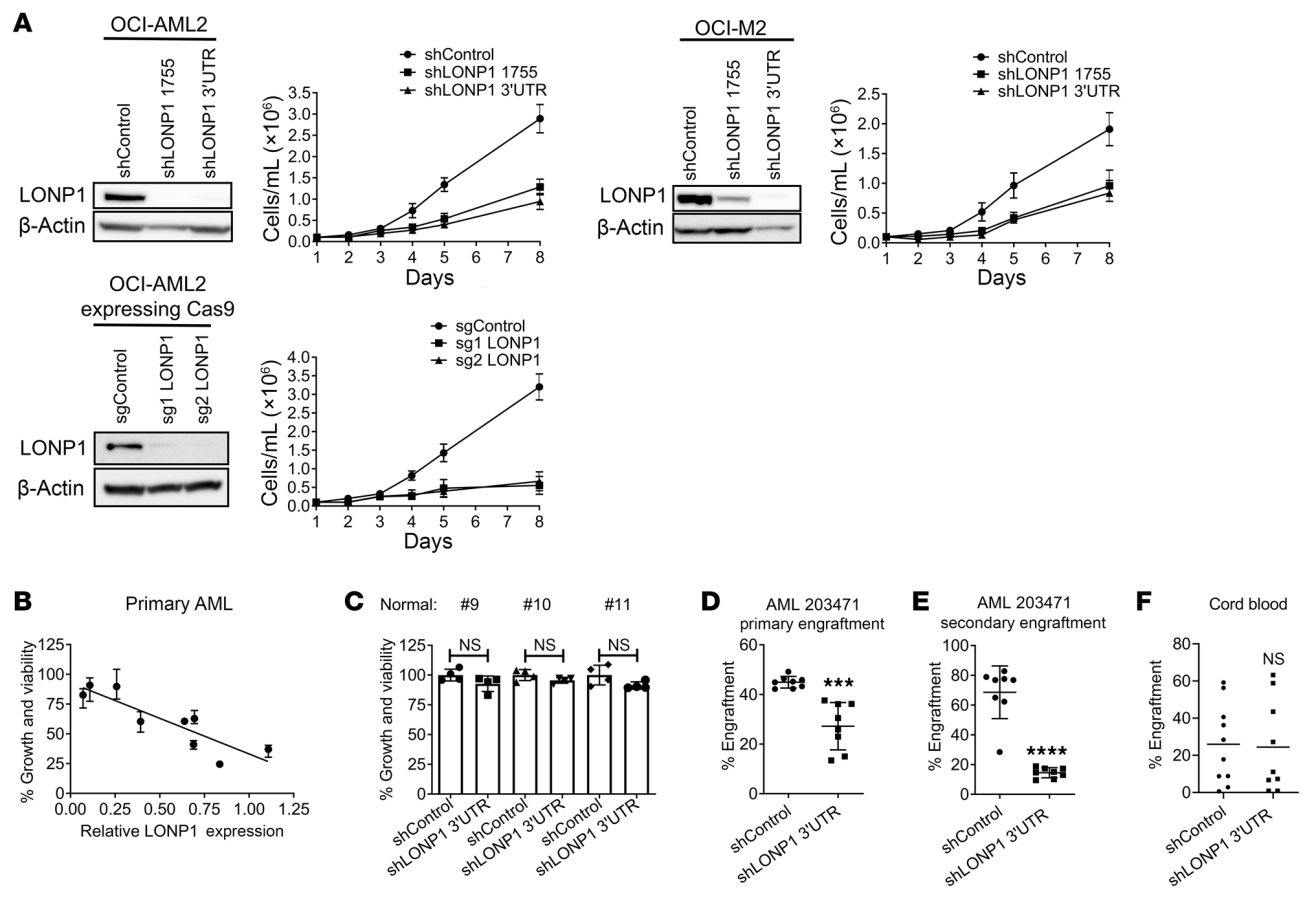
LONP1 is essential for a subgroup of AML cell lines and patient samples. (**A**) Mean ± SD growth and viability of OCI-AML2, OCI-M2, or Cas9-expressing OCI-AML2 cells by trypan blue staining after transduction with shRNA or sgRNA targeting LONP1 or control sequences. After 7 days (shRNA) or 14 days (gRNA), expression levels of LONP1 and β-actin were measured by immunoblotting. Representative data from 3 biological replicates are shown. (**B**) Primary AML samples (*n* = 9) were transduced with an shRNA targeting LONP1. After 7 days, cell viability was assessed by CellTiter-Fluor and LONP1 expression by immunoblotting. Basal protein expression before transduction relative to OCI-AML2 cells was calculated by densitometry. *r^2^* = 0.71. (**C**) Normal hematopoietic samples (*n* = 3) were transduced with an shRNA targeting LONP1 or control sequences. After 7 days, the mean ± SD cell growth, and viability was measured by CellTiter-Fluor. *P* > 0.05, by unpaired, 2-tailed Student’s *t* test. (**D**) Equal numbers of primary AML cells transduced with an shRNA targeting LONP1 or control sequences in GFP-containing vectors were injected into right femurs of NSG mice (*n* = 8 per group). After 12 weeks, mice were sacrificed, and engraftment of CD45^+^CD33^+^ cells was measured in left femurs. Transduction efficiencies were 18.5% and 16.7% for shControl and shLONP1 3′-UTR groups, respectively. ***P = 0.0002, by unpaired, 2-tailed Student’s *t* test. (**E**) Equal numbers of primary AML cells from **D** were injected into the right femurs of NSG mice (*n* = 8 per group). After 12 weeks, left femur engraftment was measured. ****P < 0.0001, by unpaired, 2-tailed Student’s *t* test. (**F**) Equal numbers of cord blood cells transduced with shRNA targeting LONP1 or control sequences in GFP-containing vectors were injected into right femurs of NS-GF mice (*n* = 9 per group). After 12 weeks, left femur engraftment was measured. Transduction efficiencies were 38.5% and 37.6% for shControl and shLONP1 3′-UTR groups, respectively. *P* = 0.0988, by unpaired, 2-tailed Student’s *t* test. Data are presented as mean ± SD.

We then evaluated the essentiality of LONP1 in AML. Knockdown of LONP1 with an shRNA reduced the growth and viability of OCI-AML2, OCI-M2, TEX, and NB4 cells (Figure 2A and Supplemental Figure 9, A and B). We also confirmed the essentiality of LONP1 in OCI-AML2 cells using CRISPR gene knockout (Figure 2A). The on-target activity of the shRNA was confirmed through rescue experiments with shRNA-resistant WT LONP1 (LONP1^WT^) cDNA (Supplemental Figure 9C).

Furthermore, we demonstrated that LONP1 was essential for the growth of AML cell lines in vivo. Knockdown of LONP1 in OCI-AML2 and TEX decreased growth and engraftment of these cells in immune deficient mice (Supplemental Figure 9, D and E). Moreover, mice xenografted with OCI-AML2 cells after LONP1 knockdown survived longer than did mice xenografted with controls cells (Supplemental Figure 9F).

Finally, we assessed the essentiality of LONP1 in primary AML and normal adult hematopoietic cells. Primary AML and normal hematopoietic cells were transduced with an shRNA targeting LONP1 or control sequences. Seven days after transduction, we measured cell viability. We found that LONP1 was variably essential in primary AML samples and that sensitivity to LONP1 depletion correlated with basal levels of LONP1. Samples with the highest basal expression of LONP1 were most sensitive to LONP1 depletion, whereas samples with lower expression were insensitive (*n* = 9, *r^2^* = 0.72) (Figure 2B). Normal adult hematopoietic cells expressed lower levels of LONP1 and were insensitive to LONP1 depletion (*n* = 3) (Figure 2C). We also assessed the effects of LONP1 depletion on the engraftment into mice of primary AML and normal hematopoietic cells. Knockdown of LONP1 decreased the engraftment of AML cells in primary and secondary engraftment studies, demonstrating an effect on the stem cell/progenitor population (Figure 2, D and E). In contrast, LONP1 knockdown did not inhibit the engraftment of human cord blood cells into NOD/SCID-3/GM/SF (NS-GF) marrow (Figure 2F).

### Chemical inhibition of the LONP1 AAA+ domain kills AML cells with high LONP1 expression.

**Figure 3:**
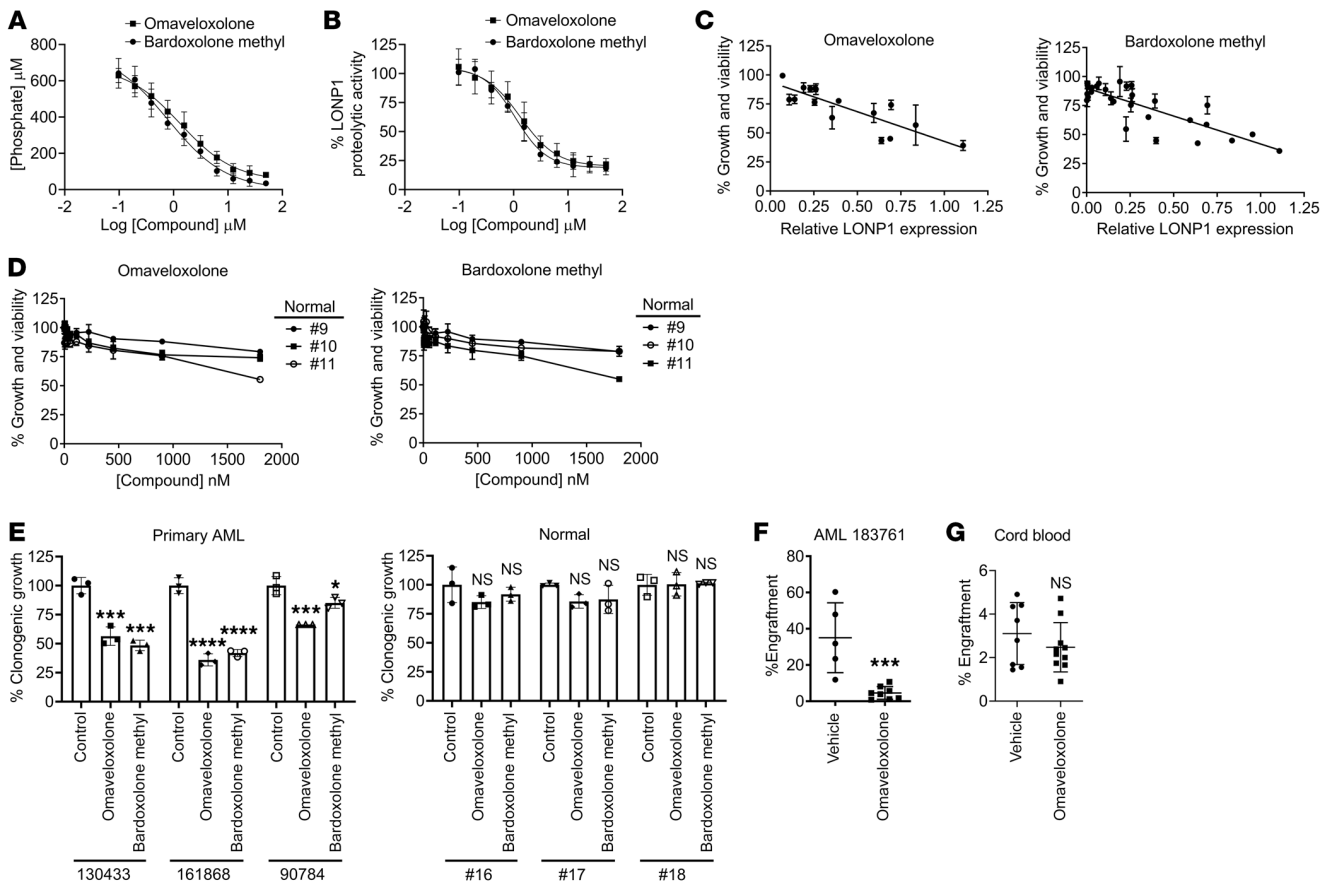
Chemical inhibition of the LONP1 AAA+ domain preferentially targets AML cells with high LONP1 expression. (**A** and **B**) Mean ± SD (**A**) ATPase activity and (**B**) proteolytic activity of recombinant LONP1 in the presence of omaveloxolone or bardoxolone methyl. (**C**) Primary AML samples were treated with 250 nM omaveloxolone (*n* = 16) or bardoxolone methyl (*n* = 30) for 72 hours. Cell growth and viability was measured by CellTiter-Fluor and LONP1 protein expression relative to OCI-AML2 cells by immunoblotting. (omaveloxolone: *r^2^* = 0.66, bardoxolone methyl: *r^2^* = 0.65). (**D**) Normal mononuclear hematopoietic cells (*n* = 3) were treated with omaveloxolone or bardoxolone methyl. After 72 hours, cell growth and viability were measured by CellTiter-Fluor. (**E**) Primary AML samples (*n* = 3) or normal mononuclear hematopoietic cells (*n* = 3) were treated with 200 nM omaveloxolone or bardoxolone methyl and plated for clonogenic growth assays. AML130433 cells (omaveloxolone: ***P = 0.0004; bardoxolone methyl: ***P = 0.0002); AML161868 cells (omaveloxolone, bardoxolone methyl: ****P < 0.0001); AML90784 cells (omaveloxolone: ***P = 0.0003; bardoxolone methyl: *P = 0.0239). NS, *P* > 0.05; 1-way ANOVA with Dunnett’s multiple-comparison test. (**F**) Primary AML cells were injected into the right femurs of NSG mice (*n* = 5–8 per group). After 6 weeks, mice were treated i.p. with omaveloxolone (7.5 mg/kg) or vehicle daily for 6 days. On day 7, engraftment of primary AML cells in the left femur was assessed with flow cytometry and anti–human CD45 and CD33 antibodies. ***P = 0.0009, by unpaired, 2-tailed Student’s *t* test. (**G**) Cord blood cells were injected into the right femurs of NS-GF mice (*n* = 8–10 per group). After 4 weeks, mice were treated with omaveloxolone (7.5 mg/kg) or vehicle for 8 weeks. Engraftment of cord blood cells in the left femur was assessed with flow cytometry and anti–human CD45 and CD33 antibodies. NS, *P* = 0.3091, by unpaired, 2-tailed Student’s *t* test. Data are presented as mean ± SD.

The synthetic triterpenoid bardoxolone methyl is a known inhibitor of LONP1 and binds to an allosteric site near the AAA+ domain (Supplemental Figure 10A) (29). Omaveloxolone is structurally related to bardoxolone methyl and was recently approved by the FDA for the treatment of Friedreich’s ataxia, a neurodegenerative disease (Supplemental Figure 10A) (30). However, the effects of omaveloxolone on LONP1 have not been reported previously. We demonstrated that omaveloxolone and bardoxolone methyl selectively inhibited the ATPase activity of recombinant LONP1 with an IC_50_ of 0.79 ± 0.61 μM and 1.33 ± 0.70 μM, respectively (Figure 3A and Supplemental Figure 10B). As ATP hydrolysis is required for the proteolytic activity of LONP1, omaveloxolone and bardoxolone methyl, expectedly, also inhibited LONP1-mediated cleavage of FITC-casein with an IC_50_ of 1.32 ± 0.62 μM and 1.08 ± 0.31 μM, respectively (Figure 3B and Supplemental Figure 10B).

We evaluated the effects of omaveloxolone and bardoxolone methyl on the growth and viability of AML cell lines. Omaveloxolone and bardoxolone methyl reduced the growth and viability of AML cells (OCI-AML2, OCI-M2, TEX, and NB4) (Supplemental Figure 10, C and D). Moreover, omaveloxolone synergized with the clinically approved anti-AML therapeutic venetoclax to increase AML cell death (Supplemental Figure 10E). While omaveloxolone and bardoxolone methyl have targets beyond LONP1 (31, 32), at low concentrations at or below the IC_50_, cell death from these drugs was specific for LONP1. At low concentrations, no further cell death was observed when treating OCI-AML2 cells depleted of LONP1. However, at higher concentrations, these drugs killed OCI-AML2 cells lacking LONP1 (Supplemental Figure 10, F–I), consistent with additional mechanisms of action at higher concentrations.

Next, we assessed the effects of these inhibitors on primary AML and normal adult hematopoietic cells. Omaveloxolone and bardoxolone methyl killed a subgroup of primary AML cells from patient samples, and sensitivity to these drugs correlated with the basal expression of LONP1. Samples with the highest LONP1 expression were most sensitive to omaveloxolone (*n* = 16, *r^2^* = 0.64) and bardoxolone methyl (*n* = 30, *r^2^* = 0.65) (Figure 3C). In contrast, inhibition of LONP1 with these drugs did not reduce the viability of adult normal hematopoietic cells obtained from consenting individuals donating peripheral blood GCSF–mobilized hematopoietic stem and progenitor cells for allotransplantation (Figure 3D). Omaveloxolone and bardoxolone methyl also inhibited the clonogenic growth of primary AML cells (*n* = 3) (Figure 3E). In contrast, the clonogenic growth of normal adult hematopoietic cells (*n* = 3) was not impaired by these drugs (Figure 3E).

Finally, we investigated the effects of systemic administration of LONP1 inhibitors in mouse models of AML. Mice xenografted with TEX, OCI-AML2, or primary AML cells were treated with either omaveloxolone or bardoxolone. Both compounds decreased tumor growth and engraftment of OCI-AML2, TEX, and primary leukemic cells in immune-deficient mice (Figure 3F and Supplemental Figure 11, A–C). LONP1 inhibition also prolonged survival of mice engrafted with AML cells (Supplemental Figure 11, D and E). In contrast, inhibition of LONP1 did not reduce the engraftment of normal cord blood cells (Figure 3G). At concentrations of omaveloxolone and bardoxolone methyl that decreased the leukemia burden, no reductions in body weights of the mice or changes in their behavior or organ histology were observed (Supplemental Figure 11, F and G).

### Inhibition of LONP1 leads to the accumulation of aggregated mitochondrial proteins in AML cells with high LONP1 expression.

LONP1 acts in concert with chaperones to solubilize mitochondrial protein precursors by folding them into mature proteins (27, 33, 34). To understand the effect of inhibiting LONP1 on mitochondrial protein solubility and aggregation, we developed an assay to visualize insoluble protein aggregates in the mitochondria. Cells were stained with proteostat, a dye that fluoresces when intercalated with aggregated proteins and Alexa Fluor 488–labeled anti-TOMM20 to mark the mitochondria. The colocalization of proteostat and TOMM20 was visualized with confocal microscopy, and the intensity of the colocalized signal was quantified by HALO image analysis software.

**Figure 4:**
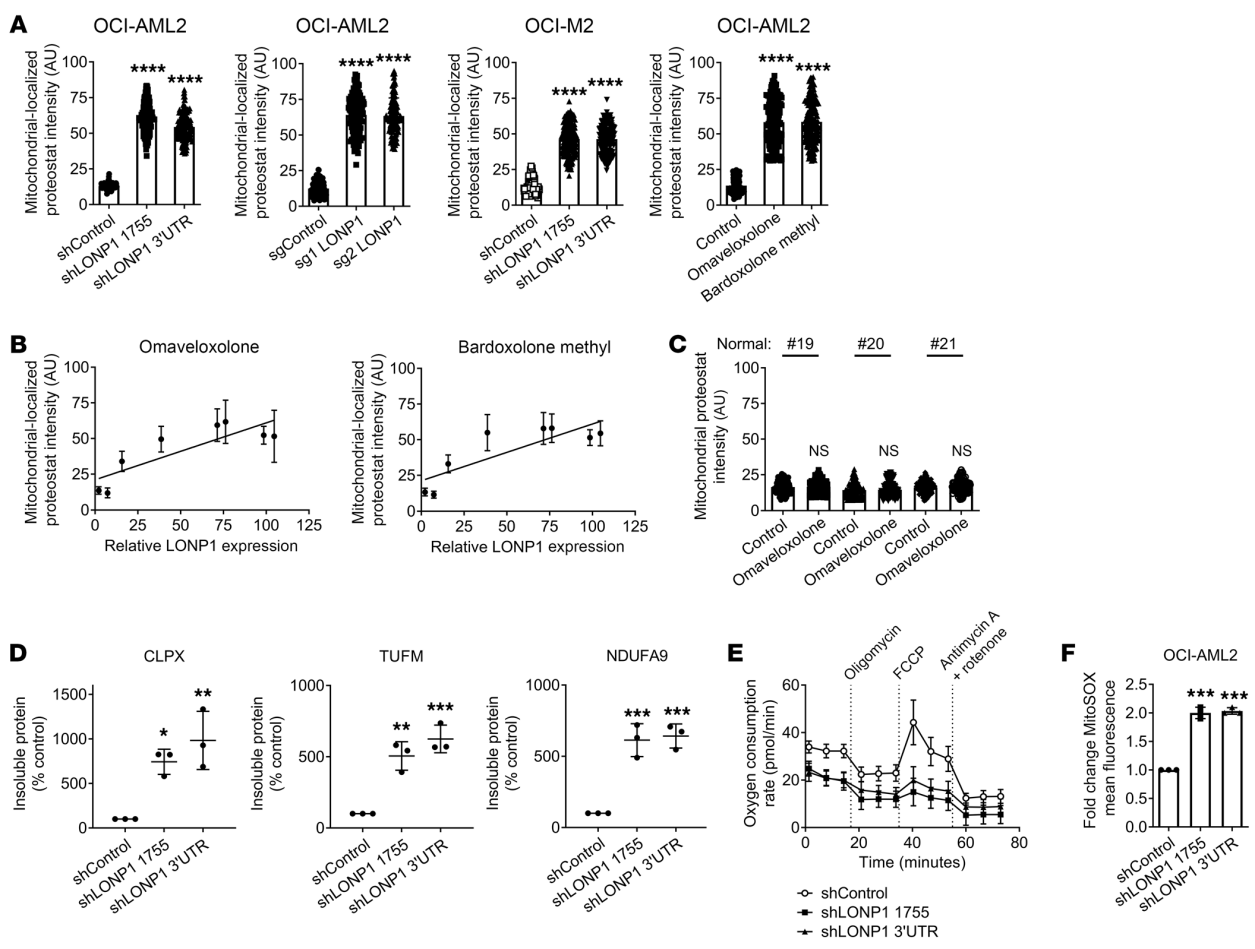
Inhibition of LONP1 leads to the accumulation of aggregated mitochondrial proteins in AML cells with high LONP1 expression. (**A**) OCI-AML2 or OCI-M2 cells were transduced with shRNA or sgRNA targeting LONP1 or control sequences or treated with 250 nM omaveloxolone or bardoxolone methyl. After 7 days (shRNA) or 14 days (gRNA) or 30 hours (chemical inhibition), cells were stained with proteostat (aggregated proteins) and FITC anti-TOMM20 (mitochondria). Cells were imaged by confocal microscopy, and colocalization of proteostat with TOMM20 was quantified by HALO software. ****P < 0.0001, by 1-way ANOVA with Dunnett’s multiple-comparison test. (**B**) Primary AML samples (*n* = 8) were treated with 250 nM omaveloxolone or bardoxolone methyl for 30 hours. Mitochondrial protein aggregation was measured as in **A**, and LONP1 expression relative to OCI-AML2 cells was measured by immunoblotting (omaveloxolone: *r^2^* = 0.64; bardoxolone methyl: *r^2^* = 0.65). (**C**) Normal mononuclear hematopoietic cells were treated with 250 nM omaveloxolone for 30 hours. Mitochondrial protein aggregation was measured as in **A**. NS, *P* > 0.05, by unpaired, 2-tailed Student’s *t* test. (**D**) OCI-AML2 cells were transduced with an shRNA targeting LONP1 or control sequences. After 7 days, LONP1 substrates (CLPX, TUFM, NDUFA9) were measured in detergent-insoluble fractions of mitochondrial lysates (Supplemental Figure 18) and quantified by densitometry. Data represent the mean ± SD of 3 biological replicates. CLPX: shLONP1 1755 (*P = 0.0152), shLONP1 3′-UTR (**P = 0.034); TUFM: shLONP1 1755 (**P = 0.0015), shLONP1 3′-UTR (***P = 0.0004), NDUFA9: shLONP1 1755 (***P = 0.0005), shLONP1 3′-UTR (***P = 0.0004) by 1-way ANOVA with Dunnett’s multiple-comparison test. (**E**) OCI-AML2 cells were transduced with an shRNA targeting LONP1 or control sequences. After 7 days, the oxygen consumption rate was measured (*n* = 14–18 per group). (**F**) OCI-AML2 cells were transduced with an shRNA targeting LONP1 or control sequences. After 7 days, mitochondrial superoxide levels were measured by flow cytometry using MitoSOX reagent. ***P = 0.0001, by 1-way ANOVA with Dunnett’s multiple-comparison test. Data are presented as mean ± SD.

We did not observe aggregated mitochondrial proteins in untreated AML cell lines or primary samples, suggesting that the UPR^mt^ and LONP1 were sufficient to maintain mitochondrial protein solubility under basal conditions. However, upon genetic depletion or chemical inhibition of LONP1, mitochondrial protein aggregates increased in AML cell lines (OCI-AML2, OCI-M2, TEX, NB4) (Figure 4A and Supplemental Figure 12–14). Likewise, inhibition of LONP1 with omaveloxolone or bardoxolone methyl induced mitochondrial protein aggregation in primary AML cells. We also observed increased mitochondrial protein aggregation in TEX cells and primary AML cells xenografted into mice treated with omaveloxolone (Supplemental Figure 15).

In primary AML, the increase in aggregated mitochondrial proteins after LONP1 inhibition positively correlated with the basal expression of LONP1 (omaveloxolone: *n* = 8, *r^2^* = 0.66; bardoxolone methyl: *n* = 8, *r^2^* = 0.65) (Figure 4B and Supplemental Figures 16 and 17). In contrast to the effects on AML, inhibiting LONP1 with omaveloxolone did not induce mitochondrial protein aggregation in normal adult hematopoietic cells (Figure 4C and Supplemental Figure 17).

We next assessed the effect of LONP1 depletion on the aggregation of specific LONP1 substrates. AML cell lines (OCI-AML2, OCI-M2, TEX, and NB4) were transduced with an shRNA or gRNA targeting LONP1 or control sequences. After transduction, mitochondrial lysates were separated into detergent-soluble and insoluble fractions. As a positive control for the aggregated protein, isolated mitochondria from cells transduced with control sequences were heat shocked at 45°C. We measured the levels of LONP1 substrates (CLPX, TUFM, and NDUFA9) in the fractions by immunoblotting and quantified them by densitometry. Genetic depletion of LONP1 decreased the abundance of substrates in the soluble fraction and promoted their accumulation in the insoluble fraction, consistent with their aggregation (Figure 4D and Supplemental Figures 18 and 19).

LONP1 substrates, such as NDUFA9, TUFM, and CLPX, are critical components of mitochondrial metabolism and oxidative phosphorylation (33, 34). As LONP1 inhibition reduced soluble levels of its substrates, we measured mitochondrial respiration and ROS production after LONP1 knockdown. Depletion of LONP1 reduced basal and maximal respiration and increased ROS production in OCI-AML2 cells (Figure 4, E and F).

### The LONP1 AAA+ domain is necessary for mitochondrial protein solubility.

**Figure 5:**
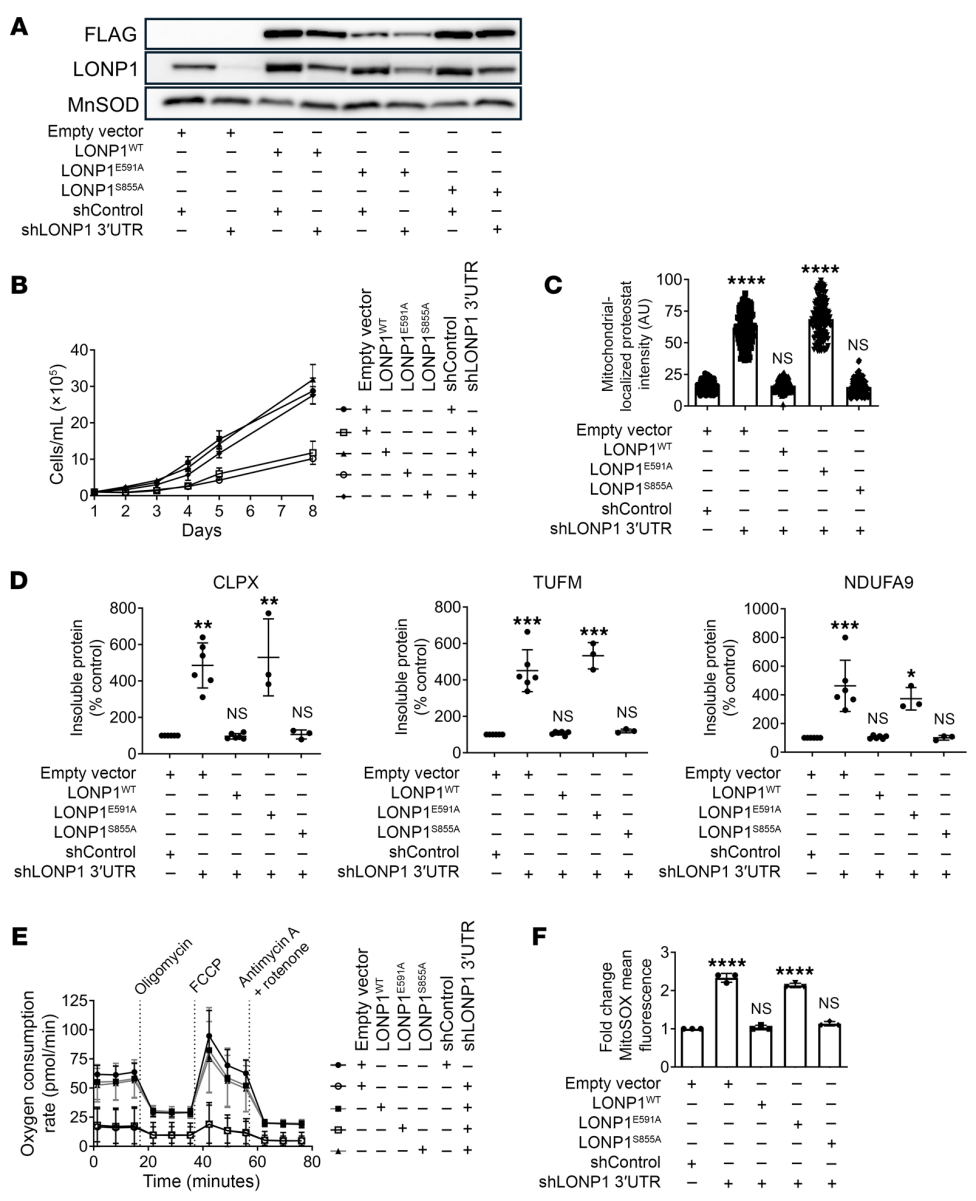
The LONP1 AAA+ domain, but not the protease domain, is necessary for mitochondrial protein solubility. (**A**) OCI-AML2 cells were transduced with empty vector, FLAG-tagged WT cDNA (LONP1^WT^), ATPase-deficient (LONP1^E591A^) cDNA, or protease-deficient LONP1 cDNA (LONP1^S855A^). Fourteen days later, cells were transduced with either an shRNA targeting the 3′-UTR of endogenous LONP1 or control sequences. After 7 days, levels of LONP1 and MnSOD protein were measured in mitochondrial lysates by immunoblotting. A representative immunoblot from 3 biological replicates is shown. (**B**) Mean ± SD growth and viability of cells from **A** were measured by trypan blue staining. Representative data from 3 biological replicates are shown. (**C**) Cells from **A** were stained as in Figure 4A to assess mitochondria-localized protein aggregation (*n* = 122–166 cells per group). ****P < 0.0001 (empty vector+shControl vs. empty vector+shLONP1 3′-UTR or LONP1^E591A^+shLONP1 3′-UTR); NS, *P* > 0.05 (empty vector+shControl vs. LONP1^WT^+shLONP1 3′-UTR or LONP1^S855A^+shLONP1 3′-UTR), by 1-way ANOVA with Dunnett’s multiple-comparison test. (**D**) Levels of CLPX, TUFM, and NDUFA9 protein in the detergent insoluble fraction of isolated mitochondria from cells in **A** were measured by immunoblotting (*n* = 3). CLPX: **P < 0.01 (empty vector+shControl vs. empty vector+shLONP1 3′-UTR or LONP1^E591A^+shLONP1 3′-UTR); NS, *P* > 0.05 (empty vector+shControl vs. LONP1^WT^+shLONP1 3′-UTR or LONP1^S855A^+shLONP1 3′-UTR). TUFM: ***P < 0.001 (empty vector+shControl vs. empty vector+shLONP1 3′-UTR or LONP1^E591A^+shLONP1 3′-UTR); NS, *P* > 0.05 (empty vector+shControl vs. LONP1^WT^+shLONP1 3′-UTR or LONP1^S855A^+shLONP1 3′-UTR). NDUFA9: ***P = 0.0004 (empty vector+shControl vs. empty vector+shLONP1 3′-UTR); *P = 0.0179 (empty vector+shControl vs. LONP1^E591A^+shLONP1 3′-UTR); NS, *P* > 0.05 (empty vector+shControl vs. LONP1^WT^+shLONP1 3′-UTR or LONP1^S855A^+shLONP1 3′-UTR). (**E**) Oxygen consumption of cells from **A** was measured with a Seahorse Metabolic Flux Bioanalyzer (*n* = 9–15 wells per group). (**F**) Mitochondrial superoxide in cells from **A** was assessed as in Figure 4F. ****P < 0.0001 (empty vector+shControl vs. empty vector+shLONP1 3′-UTR or LONP1^E591A^+shLONP1 3′-UTR); NS, *P* > 0.05 (empty vector+shControl vs. LONP1^WT^+shLONP1 3′-UTR or LONP1^S855A^+shLONP1 3′-UTR) by 1-way ANOVA with Dunnett’s multiple-comparison test. Data are presented as mean ± SD.

We next determined which domain of LONP1 is necessary for maintaining mitochondrial protein solubility. We engineered OCI-AML2 cells by overexpressing cDNA corresponding to WT LONP1 (LONP1^WT^), ATPase-deficient LOPN1 (LONP1^E591A^), or proteolytically deficient LONP1 (LONP1^S855A^) and subsequently knocked down endogenous LONP1 using an shRNA targeting the 3′-UTR of the endogenous gene. Overexpression of the LONP1 constructs and depletion of endogenous LONP1 were confirmed by immunoblotting (Figure 5A). We confirmed that the E591A mutation selectively abolished the ATPase activity of the enzyme and that the S855A mutation selectively abolished the protease activity (Supplemental Figure 20, A and B).

Overexpression of LONP1^S855A^, which retains a functional AAA+ domain, rescued OCI-AML2 cells from LONP1 depletion. These cells did not accumulate aggregated mitochondrial proteins or ROS and maintained normal oxygen consumption. These cells also proliferated normally. In contrast, overexpression of LONP1^E591A^, which retains an intact proteolytic site, failed to rescue OCI-AML2 from LONP1 inhibition. These cells displayed increased mitochondrial protein aggregation, increased ROS, decreased oxygen consumption, and decreased cell growth and viability (Figure 5, B–F, Supplemental Figure 20, C and D, and Supplemental Figure 21). Thus, taken together, the LONP1 AAA+ domain was necessary to maintain mitochondrial protein solubility, mitochondrial function, and cell viability, whereas a functional protease domain was not essential.

### Normal tissues have lower LONP1 levels and do not require LONP1 to maintain mitochondrial protein solubility.

**Figure 6:**
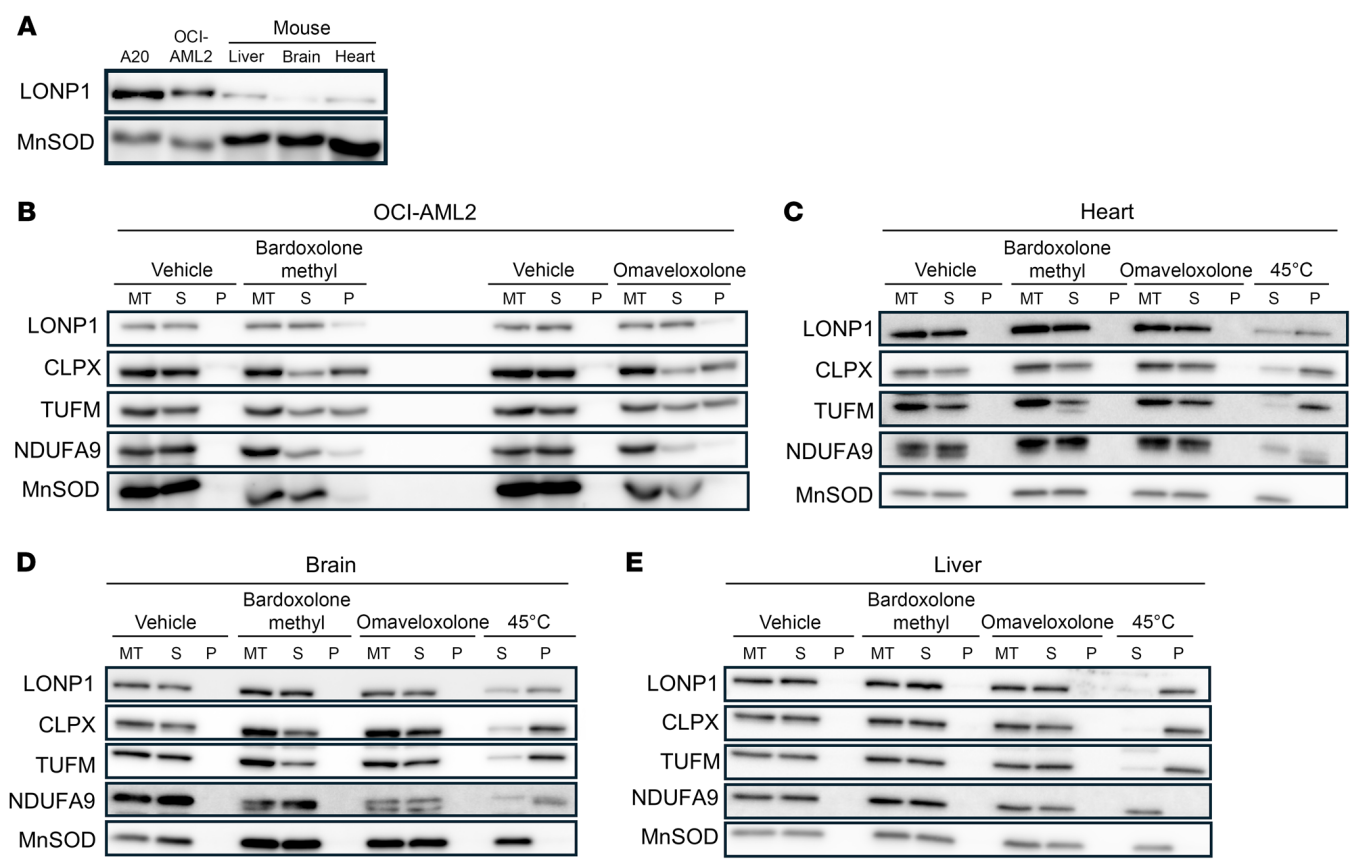
Systemic administration of LONP1 inhibitors reduces growth and increases mitochondrial protein aggregation in malignant cells while sparing normal cells. (**A**) LONP1 and Manganese Superoxide Dismutase (MnSOD) expression in mitochondria isolated from A20 and OCI-AML2 cells, and cells from liver, brain, and heart of SCID mice. (**B**–**E**) OCI-AML2 cells were injected s.c. into the flanks of SCID mice. One week after injection, mice were treated with 7.5 mg/kg omaveloxolone, bardoxolone methyl, or vehicle control daily for 6 consecutive days. Mitochondria were isolated from (**B**) OCI-AML2 tumors and murine (**C**) heart, (**D**) brain, and (**E**) liver. Levels of LONP1, CLPX, TUFM, NDUFA9, and MnSOD were measured in total (MT), soluble (S), and insoluble pellet (P) fractions of mitochondrial lysates. Data are presented as mean ± SD.

Finally, to understand why normal tissues were not dependent on LONP1, we measured LONP1 levels in normal murine tissues. LONP1 expression was lower in normal mitochondria-rich murine tissues (liver, brain, heart) compared with OCI-AML2 and A20 murine leukemia/lymphoma cells (Figure 6A). Of note, human and murine LONP1 share 81% LONP1 sequence homology (35), and we found that omaveloxolone increased mitochondrial protein aggregation and decreased the growth and viability of A20 murine leukemia cells, similar to human AML cells, confirming cross-reactivity with murine LONP1 (Supplemental Figure 22).

We also measured changes in mitochondrial protein aggregation in murine tissue after treatment with omaveloxolone and bardoxolone methyl. Mice xenografted with OCI-AML2 cells were treated with omaveloxolone or bardoxolone methyl daily for 6 days. After treatment, mitochondria from the leukemic cells as well as mouse livers, brains, and hearts were harvested. We found that both drugs increased aggregation of LONP1 substrates in the leukemic cells (Figure 6B). In contrast, mitochondrial protein aggregates did not accumulate in murine tissues (Figure 6, C–E). Thus, taken together, LONP1 inhibition led to mitochondrial protein aggregation in AML cells with high LONP1 expression. In contrast, normal tissues expressed lower levels of LONP1 and did not accumulate mitochondrial protein aggregates upon LONP1 inhibition, thus explaining the therapeutic window for this target.

In conclusion, cells from a subgroup of patients with AML had increased mitochondrial protein import and upregulation of the UPR^mt^ to counter the stress of newly imported proteins. Targeting components of the UPR^mt^, such as LONP1, led to increased mitochondrial protein aggregation and cell death in this subset of cells.

## Discussion

Once imported into the mitochondria, proteins are processed into their mature forms with the aid of chaperones and proteases (1–4). Failure to process these newly imported proteins leads to protein aggregation, which can be toxic to the mitochondria and the cell (2–4). Cells therefore developed the UPR^mt^ to cleave, fold, and degrade newly imported, as well as damaged, mitochondrial proteins (5–8, 22). Here, we discovered that the UPR^mt^ was upregulated in AML cells as a response to increased mitochondrial protein import. Targeting the UPR^mt^ at the level of LONP1 selectively induced mitochondrial protein aggregation and cell death in AML samples with high LONP1 but spared AML cells and normal tissues with lower rates of mitochondrial protein import and LONP1 levels.

Our group and others have shown that AML and AML stem cells exhibit increased mitochondrial biogenesis and a heightened reliance on mitochondrial metabolism for survival and proliferation (11–13, 15). In line with previous reports (11–13, 15), we confirmed that a subgroup of patients with AML had increased mitochondrial gene expression. To import proteins into the mitochondria, precursors with mitochondrial localization sequences enter through the translocase TOMM40 and are then sorted into different subcompartments (1, 2). We developed an assay to assess protein import. Compared with normal hematopoietic cells, we discovered that mitochondrial protein import was increased in AML cells, probably to support their heightened reliance on mitochondrial metabolism. This increased rate of import may also reflect higher turnover of mitochondrial proteins damaged by factors such as ROS (11, 16, 36, 37).

Protein precursors are imported into the mitochondria in an unfolded state (3, 4). If not properly folded, mitochondrial precursors will aggregate, which is toxic to the mitochondria and the cell (21, 38). To protect the mitochondria from the stress of newly imported proteins and damage to mature proteins, cells developed the evolutionarily conserved UPR^mt^ (5–8, 22). Although many of the UPR^mt^ components have been defined, the importance of the UPR^mt^ in cancer and leukemia remains uncertain. To our knowledge, ours is the first study to show that the UPR^mt^ is increased in a subgroup of leukemic cells as a response to increased mitochondrial protein import and is necessary to maintain mitochondrial protein solubility. Of note, while the UPR^mt^ and mitochondrial protein import were increased in AML, they were equally upregulated across the cytogenetic and molecular subtypes of AML. Thus, an increase in protein import and the UPR^mt^ are potentially downstream consequences of multiple molecular mutations. Future studies will determine whether the mitochondria in AML are intrinsically abnormal or adapt to the malignant state of the cells.

It is unknown whether the increased protein import and UPR^mt^ are early or late events in the pathogenesis of AML. However, recent studies have reported mitochondrial dysfunction in preleukemia and clonal hematopoiesis, suggesting that increased mitochondrial stress may also be an early event (39). For example, murine cells with isolated mutations in DMNT3A, the most common mutation in preleukemia/clonal hematopoiesis, display increased mitochondrial oxidative metabolism and rewire 1 carbon metabolic pathway (39). Thus, these results suggest that altered mitochondrial function is an early event in the pathogenesis of AML (39). Further studies will determine whether increased protein import and UPR^mt^ are also upregulated in the early stages of leukemia and how their upregulation may contribute to malignant transformation.

Leading-edge analysis of the UPR^mt^ signature identified LONP1 as a top gene in this pathway. We discovered that LONP1 was variably essential in AML samples. Genetic or chemical inhibition of LONP1 induced mitochondrial protein aggregation and cell death in samples from patients with primary AML with the highest levels of LONP1, whereas AML patient samples with low levels of LONP1 and normal hematopoietic cells were insensitive to LONP1 inhibition. Likewise, in mouse models, systemic administration of LONP1 inhibitors induced mitochondrial protein aggregation in leukemic cells but not normal cells, including in mitochondria-rich organs such as the liver and heart. Despite being mitochondria-rich, LONP1 expression in these cells was lower than the expression in leukemic cells, potentially reflecting lower rates of mitochondrial protein import and turnover in these normal tissues. Thus, these results support a therapeutic window for LONP1 inhibition and the development of LONP1 inhibitors for the subset of patients with AML with high expression of the target.

Unfolded protein precursors must be properly folded lest they aggregate, which is toxic to the mitochondria and the cell. The accumulation of aggregated proteins in the mitochondria is toxic to normal and malignant cells. Toxicity occurs as a result of loss of soluble mitochondrial proteins such as respiratory chain proteins, leading to impaired mitochondrial function. In addition, aggregated mitochondrial proteins are directly toxic to the mitochondria and cell. For example, overexpression of aggregation-prone ornithine transcarbamylase in *Drosophila* leads to mitochondrial protein aggregation, reduced respiratory chain function, and death (38).

While, to our knowledge, we are the first to study LONP1 in hematologic malignancies and report its effects on maintaining protein solubility in AML, prior studies indicate LONP1 to be an anticancer target in solid tumors. For example, inhibition of LONP1 kills colon (40, 41), pancreatic (42), and prostate (43) cancer cells in vitro and in vivo.

We demonstrated that the triterpenoids omaveloxolone and bardoxolone methyl inhibited LONP1 to induce mitochondrial protein aggregation and cell death in AML cells with high LONP1 expression. Omaveloxolone was recently approved by the FDA for the treatment of Friedreich’s ataxia (30), and bardoxolone methyl advanced to a phase III trial for the treatment of renal failure, although it did not meet its efficacy endpoints (44). Prior studies evaluated bardoxolone methyl in phase I clinical trials for patients with advanced cancers (45). However, the doses used were probably too low to inhibit LONP1, and the trials did not select for tumors with high LONP1 expression. Nonetheless, at the higher doses used in the treatment of renal failure (44) and Friedreich’s ataxia (30), plasma concentrations that inhibit LONP1 are likely achievable, and the drugs have a relatively favorable safety profile. Bardoxolone methyl is associated with an increased risk of congestive heart failure (absolute risk 3%) (44, 46), but the increased risk was completely mitigated by excluding patients with cardiac histories (47). No increased incidence of cardiac events was reported for omaveloxolone, probably because the trials specifically excluded patients with existing cardiac conditions (48). Thus, these drugs could be rapidly advanced to clinical trial for patients with AML and high LONP1 expression.

We showed that bardoxolone methyl inhibited LONP1 ATP hydrolysis, in line with a recent study (29), and, to our knowledge, we are the first to report that omaveloxolone also binds and inhibits LONP1 ATPase and proteolytic activity. At concentrations at or below the IC_75_, omaveloxolone and bardoxolone methyl specifically inhibited LONP1. However, we concede that these drugs have additional targets, because at higher doses, these drugs had effects beyond inhibition of LONP1 and killed cells lacking LONP1 (30, 44). Notably, prior studies reported multiple cellular effects of triterpenoids including activation of Nrf-2 (49) and inhibition of NF-κB (50). Thus, while useful as chemical probes, LONP1 inhibitors with greater specificity would be important to fully understand the biology and therapeutic potential of this protein.

Developing more specific inhibitors would be aided by physical structures of omaveloxolone and bardoxolone methyl in complex with LONP1. A recent in silico study proposed a binding site for bardoxolone methyl between the ATPase domains of adjacent subunits, thereby providing an explanation for how this drug can allosterically inhibit the enzyme (29). However, mutating residues Cys576 and Cys637 at the putative binding site paradoxically enhanced the ability of bardoxolone methyl to inhibit LONP1 (29). Thus, determining the experimental high-resolution structures of these drugs in complex with LONP1 remains important.

Our work reveals a therapeutic window for LONP1 inhibition in AML cells compared with normal cells. Through gene expression and functional studies, we demonstrated that normal cells had lower levels of mitochondrial import compared with AML cells. As such, these normal cells were under less mitochondrial stress from the load of newly imported proteins, as evidenced by lower levels of the UPR^mt^ and LONP1. As a result, partially and/or transiently inhibiting LONP1 did not lead to mitochondrial protein aggregation or cell death in these normal cells. In contrast, we found that a subgroup of AML cells had higher rates of mitochondrial protein import and were under increased mitochondrial stress with higher UPR^mt^ and LONP1 levels. In these cells, inhibition of LONP1 led to mitochondrial protein aggregation and cell death. As such, we propose that the preferential sensitivity of AML cells to LONP1 inhibition compared with normal cells relates to the higher rates of mitochondrial protein import and mitochondrial stress in these AML cells.

In further support of a preferential effect of LONP1 inhibition on malignant cells over normal cells, we note a prior study by Quiros et al., who discovered that LONP1^+/–^ heterozygous–knockout mice are viable with no substantial phenotype, despite a 50% reduction in LONP1 protein and mRNA levels (41). Yet, these mice are protected from chemically induced colon and skin tumors (41). However, we also concede that sustained and total LONP1 inhibition may be toxic to normal cells, as LONP1^–/–^ homozygous–knockout mice are not viable and die in the embryonic phase at postcoitum day 8.5 (41).

In conclusion, we discovered that a subgroup of patients with AML had increased mitochondrial protein import, resulting in upregulation of the UPR^mt^. In these cells, the UPR^mt^ was necessary to maintain mitochondrial protein solubility, mitochondrial function, and cell viability. Targeting the UPR^mt^ at the level of the LONP1 AAA+ domain selectively disrupted proteostasis within AML cells while sparing normal hematopoietic cells. Thus, our work highlights the biological importance of the UPR^mt^ in AML and advances strategies to target mitochondrial stress in this disease.

## Methods

### Sex as a biological variable.

Our animal studies with SCID and NS-GF mice used male and female mice in comparable ratios and produced similar results between sexes. For experiments with NSG mice, only female mice were used, as primary AML cells engraft better in NSG female mice.

### Experimental design.

The purpose of this study was to determine the role of mitochondrial protein import and UPR^mt^ components in maintaining the unique mitochondrial reliance in AML. The expression of genes associated with protein import and the UPR^mt^ in AML and normal hematopoietic cells was determined by analysis of publicly available datasets. Protein import was assessed with confocal microscopy. The essentiality of UPR^mt^ genes was ranked in the DepMap datasets. LONP1 expression in AML and normal cells was determined by analysis of publicly available datasets and immunoblotting. The role of LONP1 was assessed by genetic (shRNA and CRISPR) and chemical (omaveloxolone and bardoxolone methyl) inhibition in vitro and in vivo. Mitochondrial protein solubility was assessed with confocal microscopy and immunoblotting. For in vivo studies, mice were randomly assigned to treatment arms. For patient-derived xenograft murine models, the primary endpoint was leukemia burden in bone marrow at the end of the study.

Additional materials and methods are provided in Supplemental Methods.

### Analysis of gene expression in AML and normal hematopoietic cells.

For the gene sets, human genes encoding proteins with strong support of mitochondria localization were obtained from MitoCarta 3.0. The 13 mitochondrial encoded genes were removed from the list, resulting in 1,144 mitochondrial nuclear coded genes used for the analysis.

The mitochondrial protein import gene set GO 0072655, named “establishment of protein localization to mitochondrion,” was retrieved from the GO Biological Process (GO BP) database.

For the Haferlach AML cohort (51), Affymetrix gene expression data on AML (*n* = 542 samples) and normal bone marrow mononuclear samples (*n* = 73 samples) from the Haferlach dataset (GSE13159) was downloaded from the Gene Expression Omnibus (GEO) database. The platform used was the Affymetrix Human Genome U133 Plus 2.0 Array, and the data values corresponded to the robust multichip average (RMA) expression measure. Official gene symbols from the Hugo Gene Nomenclature Committee were retrieved from the Affymetrix probe identifiers using the R package biomaRt. Data were reduced at the gene level by selecting the probe with the highest median absolute deviation (MAD) across samples per gene.

The median expression of the mitochondrial genes, protein import, and UPR^mt^ pathways was calculated for each AML and healthy bone marrow sample. For the protein import and UPR^mt^ pathways, the median expression was divided into quartile expression groups, and the 25th and 75th percentiles were used to define low and high expression groups, respectively. The 25th and 75th percentiles were also used to divide AML samples into low and high expression groups using *LONP1* gene expression. To estimate the correlation between the different gene sets and between *LONP1* expression, a linear regression model was fitted to the data using ggplot2_3.5.1 [geom_smooth(method=lm)]. The Pearson correlation coefficient was calculated separately using cor.test().

Differential expression analysis between AML and normal and high and low expression groups was performed using a moderated *t* test in limma_3.58.1. Genes were ranked from top upregulated to downregulated genes using the *t* values, and the rank files were used in the gene set enrichment analysis (GSEA) called from clusterProfiler_4.10.1 against the different gene sets using 2,000 permutations.

The clusterProfiler_4.10.1 package was used to generate the GSEA plots to visualize the results. Results were also visualized via box plots, scatter plots and dot plots using ggplot2_3.5.1, and heatmaps were generated using pheatmap_1.0.12.

For the BeatAML2 cohort, batch-corrected and normalized expression data on BeatAML2 RNA-seq samples were downloaded from https://biodev.github.io/BeatAML2/ (52). Mean expression of the mitochondrial genes, protein import, and the UPR^mt^ pathways was calculated for each AML and healthy bone marrow mononuclear cell sample.

The list of AMLs with mean expression of mitochondrial protein import, the UPR^mt^ and, LONP1 expression above the 90th percentile of the normal samples was retrieved. These AML samples were associated with clinical/laboratory data (FAB classification, ELN2017 risk frequent mutations and fusion proteins), and the distribution profile of each category was compared with the profile of total AML cell samples using a Fisher’s s exact test. Tests were corrected for multiple hypothesis testing using the Benjamini-Hochberg method. Data were visualized by pie chart using ggplot2, version 4.0.1.

The median expression values for mitochondrial protein import, the UPR^mt^, and LONP1 expression were used to split AML samples into 2 categories. Kaplan-Meier curves depicting overall survival were generated, and differences between groups were assessed using a log-rank test, with analyses performed using the survminer (version 0.5.0) and survival 393 (version 3.8.3) R packages.

For the AML proteomics dataset, imputed normalized data from Kramer et al. (53) were used to split samples into 2 categories according to the median expression of mitochondrial protein import, the UPR^mt^, and LONP1 expression. Kaplan-Meier curves depicting overall survival were generated, and differences between groups were assessed using a log-rank test, with analyses performed using the survminer (version 0.5.0) and survival (version 3.8.3) R packages.

The proteomics dataset (53) was also used to visualize the risk (ELN2017), FAB, and mutations categories in correlation with the mean expression of mitochondrial protein import and UPR^mt^ as well as LONP1 gene expression. A Wilcoxon rank-sum test was performed to assess whether each category was associated with significantly higher or lower gene expression levels. Tests were corrected for multiple hypothesis testing using the Benjamini-Hochberg method. Ridge plots were created using ggplot2, version 3.5.2.

Bioinformatics analyses were conducted in R, version 3.4.2.

### Statistics.

GraphPad Prism 8.0 (GraphPad Software) was used to perform data plotting and statistical analysis. Data are presented as the mean ± SD unless otherwise stated. An unpaired, 2-tailed Student’s *t* test was used to calculate significance between 2 groups. A Fisher’s exact test was used to estimate count difference in categories of AMLs. A 1-way ANOVA followed by Dunnett’s or Kruskal Wallis post hoc testing was used to compare the means of more than 2 groups. The log-rank test was used to measure significance in Kaplan-Meier plots for survival curves. A *P* value of less than 0.05 was considered significant.

### Study approval.

All animal experiments were performed in accordance with institutional guidelines approved by the University Health Network Animal Care Committee under Animal Use Protocol no. 1251 (Toronto, Ontario, Canada). The University Health Network IRBs approved the collection and use of human tissue for this study (REB no. 13-7163).

### Data and materials availability.

Raw data for this work are provided in the Supporting Data Values file. Contact the corresponding author to request additional data and materials.

## Author contributions

MT, VV, and ADS designed the study, analyzed the results, and wrote the manuscript. VV, AC, and CS analyzed the bioinformatics data. MDM and AA provided clinical annotated samples from the Princess Margaret Cancer Center AML Biobank. RSM and MAR synthesized the bortezomib used in this study. MT, VV, GET, AAP, MG, RH, DL, YY, LXZ, YF, AC, ND, ZM, BS, SK, YJ, EA, and PYM performed the experiments and analyzed the data. MT, VV, GET, AAP, MG, RH, DL, YY, LXZ, YF, AC, ND, RSM, SQWC, ZM, BS, SK, YJ, CS, EA, PYM, AA, TK, MAR, BZC, MA, SMK, MDM, SV, and ADS reviewed the manuscript. ADS, MDM, SV, SMK, MA, BZC, MAR, and TK provided funding and supervised the study.

## Conflict of interest

ADS has received research funding from Takeda Pharmaceuticals, BMS, and Medivir AB, and has received consulting fees/honoraria from Takeda, Astra-Zeneca, BMS, and Novartis, Pharmaceuticals. ADS is on the medical and scientific board of the Leukemia and Lymphoma Society of Canada. ADS is an inventor of DNT cell technology related patents and intellectual properties for the treatment of AML (“Methods For Enhancing T Cells Using Venetoclax. United States Provisional Application” No. 62/971,534).

## Funding support

- Canadian Institutes of Health Research (CIHR Foundation grant FDN-154282).
- Princess Margaret Cancer Centre Foundation.
- ADS holds the Ronald N. Buick Chair in Oncology Research.
- EA is a TRIUMPH Fellow in the CPRIT Training Program (RP210028).

## Supplementary Material

Supplemental data

Unedited blot and gel images

Supporting data values

## Version 1 06/16/2026

In-Press Preview

## Version 2 08/03/2026

Electronic publication

## References

[1] Wiedemann N, Pfanner N (2017). Mitochondrial machineries for protein import and assembly. Annu Rev Biochem.

[2] Ruan L (2020). Mitochondria-associated proteostasis. Annu Rev Biophys.

[3] Matouschek A (1997). Active unfolding of precursor proteins during mitochondrial protein import. EMBO J.

[4] Wilcox AJ (2005). Effect of protein structure on mitochondrial import. Proc Natl Acad Sci U S A.

[5] Fiorese CJ (2016). The transcription factor ATF5 mediates a mammalian mitochondrial UPR. Curr Biol.

[6] Melber A, Haynes CM (2018). UPR^mt^ regulation and output: a stress response mediated by mitochondrial-nuclear communication. Cell Res.

[7] Horibe T, Hoogenraad NJ (2007). The chop gene contains an element for the positive regulation of the mitochondrial unfolded protein response. PLoS One.

[8] Aldridge JE (2007). Discovery of genes activated by the mitochondrial unfolded protein response (mtUPR) and cognate promoter elements. PLoS One.

[9] Frakes AE, Dillin A (2017). The UPR^ER^: sensor and coordinator of organismal homeostasis. Mol Cell.

[10] Kantarjian H (2021). Acute myeloid leukemia: current progress and future directions. Blood Cancer J.

[11] Sriskanthadevan S (2015). AML cells have low spare reserve capacity in their respiratory chain that renders them susceptible to oxidative metabolic stress. Blood.

[12] Pollyea DA (2018). Venetoclax with azacitidine disrupts energy metabolism and targets leukemia stem cells in patients with acute myeloid leukemia. Nat Med.

[13] Jones CL (2018). Inhibition of amino acid metabolism selectively targets human leukemia stem cells. Cancer Cell.

[14] Jones CL (2020). Nicotinamide metabolism mediates resistance to venetoclax in relapsed acute myeloid leukemia stem cells. Cell Stem Cell.

[15] Lagadinou ED (2013). BCL-2 inhibition targets oxidative phosphorylation and selectively eradicates quiescent human leukemia stem cells. Cell Stem Cell.

[16] Farge T (2017). Chemotherapy-resistant human acute myeloid leukemia cells are not enriched for leukemic stem cells but require oxidative metabolism. Cancer Discov.

[17] Döhner H (2017). Diagnosis and management of AML in adults: 2017 ELN recommendations from an international expert panel. Blood.

[18] Dabir DV (2013). A small molecule inhibitor of redox-regulated protein translocation into mitochondria. Dev Cell.

[19] Schafer JA (2022). Global mitochondrial protein import proteomics reveal distinct regulation by translation and translocation machinery. Mol Cell.

[20] Singh RP (2020). Disrupting mitochondrial copper distribution inhibits leukemic stem cell self-renewal. Cell Stem Cell.

[21] Balchin D (2016). In vivo aspects of protein folding and quality control. Science.

[22] Zhao Q (2002). A mitochondrial specific stress response in mammalian cells. EMBO J.

[23] Xin N (2022). The UPRmt preserves mitochondrial import to extend lifespan. J Cell Biol.

[24] Poveda-Huertes D (2021). Increased mitochondrial protein import and cardiolipin remodelling upon early mtUPR. PLoS Genet.

[25] Uoselis L (2023). Temporal landscape of mitochondrial proteostasis governed by the UPR^mt^. Sci Adv.

[26] Tsherniak A (2017). Defining a cancer dependency map. Cell.

[27] Deshwal S (2020). Mitochondrial proteases: multifaceted regulators of mitochondrial plasticity. Annu Rev Biochem.

[28] Gibellini L (2020). The biology of Lonp1: More than a mitochondrial protease. Int Rev Cell Mol Biol.

[29] Lee J (2022). Inhibition of mitochondrial LonP1 protease by allosteric blockade of ATP binding and hydrolysis via CDDO and its derivatives. J Biol Chem.

[30] Lynch DR (2021). Safety and efficacy of omaveloxolone in friedreich ataxia (MOXIe Study). Ann Neurol.

[31] Liby K (2007). The synthetic triterpenoids CDDO-methyl ester and CDDO-ethyl amide prevent lung cancer induced by vinyl carbamate in A/J mice. Cancer Res.

[32] Abeti R (2018). Novel Nrf2-inducer prevents mitochondrial defects and oxidative stress in friedreich’s ataxia models. Front Cell Neurosci.

[33] Shin CS (2021). LONP1 and mtHSP70 cooperate to promote mitochondrial protein folding. Nat Commun.

[34] Matsushima Y (2021). Mitochondrial Lon protease is a gatekeeper for proteins newly imported into the matrix. Commun Biol.

[35] Camacho C (2009). BLAST+: architecture and applications. BMC Bioinformatics.

[36] Hole PS (2013). Overproduction of NOX-derived ROS in AML promotes proliferation and is associated with defective oxidative stress signaling. Blood.

[37] Pei S (2013). Targeting aberrant glutathione metabolism to eradicate human acute myelogenous leukemia cells. J Biol Chem.

[38] Pimenta De Castro I (2012). Genetic analysis of mitochondrial protein misfolding in Drosophila melanogaster. Cell Death Differ.

[39] Hosseini M (2025). Metformin reduces the competitive advantage of Dnmt3a^R878H^ HSPCs. Nature.

[40] Gibellini L (2018). LonP1 differently modulates mitochondrial function and bioenergetics of primary versus metastatic colon cancer cells. Front Oncol.

[41] Quirós PM (2014). ATP-dependent Lon protease controls tumor bioenergetics by reprogramming mitochondrial activity. Cell Rep.

[42] Liu C (2019). Inhibition of LONP1 suppresses pancreatic cancer progression Via c-Jun N-terminal kinase pathway-meditated epithelial-mesenchymal transition. Pancreas.

[43] Yao M (2025). Lon protease 1-mediated metabolic reprogramming promotes the progression of prostate cancer. Cell Death Dis.

[44] de Zeeuw D (2013). Bardoxolone methyl in type 2 diabetes and stage 4 chronic kidney disease. N Engl J Med.

[45] Hong DS (2012). A phase I first-in-human trial of bardoxolone methyl in patients with advanced solid tumors and lymphomas. Clin Cancer Res.

[46] Chin MP (2014). Risk factors for heart failure in patients with type 2 diabetes mellitus and stage 4 chronic kidney disease treated with bardoxolone methyl. J Card Fail.

[47] Chertow GM (2021). Study Design and Baseline Characteristics of the CARDINAL trial: a phase 3 study of bardoxolone methyl in patients with alport syndrome. Am J Nephrol.

[48] Lynch DR (2023). Efficacy of omaveloxolone in friedreich’s ataxia: delayed-start analysis of the MOXIe extension. Mov Disord.

[49] Tian C (2019). Therapeutic effects of Nrf2 activation by bardoxolone methyl in chronic heart failure. J Pharmacol Exp Ther.

[50] Ahmad R (2006). Triterpenoid CDDO-Me blocks the NF-kappaB pathway by direct inhibition of IKKbeta on Cys-179. J Biol Chem.

[51] Haferlach T (2010). Clinical utility of microarray-based gene expression profiling in the diagnosis and subclassification of leukemia: report from the International Microarray Innovations in Leukemia Study Group. J Clin Oncol.

[52] Bottomly D (2022). Integrative analysis of drug response and clinical outcome in acute myeloid leukemia. Cancer Cell.

[53] Kramer MH (2022). Proteomic and phosphoproteomic landscapes of acute myeloid leukemia. Blood.

