# Supplementary material for: Elevated mitochondrial protein import in acute myeloid leukemia increases reliance on mitochondrial protease LONP1
Source: J Clin Invest. 2026 Jun 16;136(15):e196687. doi: 10.1172/JCI196687 (PMC13430017; doi:10.1172/JCI196687)
Supplement: Supplemental data [file jci-136-196687-s085.pdf]

Supplementary Materials

Supplementary materials and methods

Supplementary figures S1-S22

Supplementary tables 1 - 3

Supplementary materials and methods

Cell Lines

OCI-AML2 and OCI-M2 cells were grown in Iscove's modified Dulbecco's medium (IMDM; Wisent), supplemented with 10% fetal bovine serum (FBS; Hyclone), 100 U/ml penicillin (Wisent) and 100 µg/ml streptomycin (Wisent). NB4 cells and A20 cells were grown in Roswell Park Memorial Institute 1640 medium (RPMI; Wisent) supplemented with 10% FBS, 100 U/ml penicillin and 100 µg/ml streptomycin. TEX cells were grown in IMDM supplemented with 20% FBS, 2 mM L-glutamine (Thermo Fisher Scientific), 100 U/mL penicillin and 100 µg/mL streptomycin, 20 ng/mL stem cell factor (SCF; Peprotech), and 2 ng/mL interleukin-3 (IL-3; Peprotech). All cell lines were grown in a 37°C, 5% CO_2 incubator. All cell lines tested negative for mycoplasma using the MycoAlert mycoplasma detection kit (Lonza). Information regarding the AML cell lines and the source of the cells is provided in Supplementary Table 3.

Primary AML and normal hematopoietic cells

Primary human AML samples with a malignant cell frequency of 80% among mononuclear cells were obtained from peripheral blood of both male and female patients with AML, following informed consent. Normal hematopoietic cells were obtained from healthy consenting male or female adult volunteers donating cells for allogenic stem cell transplantation. Cells were isolated by G-CSF stimulation followed by leukapheresis, and obtained from the Leukemia Tissue Bank at the Princess Margaret Cancer Centre. The CD34+ subpopulation of normal hematopoietic cells was isolated by EasySep™ Human CD34 Positive Selection Kit II (Stem Cell Technologies) prior to western blot analysis. Human CD34+ cord blood cells were obtained from StemCell Technologies (Cat#

70007.1). Approval for the collection and use of the primary samples was obtained from the UHN Research Ethics Board. AML cells were isolated using Ficoll-Paque differential density centrifugation and subsequently frozen in a solution comprising of 50% FBS, 40% alpha-MEM (Wisent), and 10% dimethylsulfoxide (DMSO; Sigma Aldrich). Prior to receiving cells from the tissue bank, specimens were de-identified. For all experiments, primary AML and normal hematopoietic cells were maintained in X-VIVO 10 medium (Lonza) supplemented with 20% BIT 9500 Serum Substitute (BIT; StemCell Technologies), 50 ng/ml Fms-like tyrosine kinase 3 (Flt3-L; Peprotech), 10 ng/mL interleukin 6 (IL6; Peptrotech), 50 ng/mL SCF, 25 ng/mL thrombopoietin (TPO; Peprotech), 10 ng/mL IL3, 10 ng/mL granulocyte colony stimulating factor (G-CSF; Peprotech). Information regarding the patients who were the source of the cells is provided in Supplementary Table 2.

Mitochondrial protein import assay

Fresh primary AML and normal hematopoietic cells derived from consenting individuals donating peripheral blood GCSF mobilized hematopoietic stem and progenitor cells for allotransplant (2x10^6 cells) were treated with either vehicle control, 50 μM cycloheximide (Sigma Aldrich), or a combination of 5 μM FCCP and 50 μM Mitoblock-6 (MedChemExpress) for 6 hours. 1 μg/mL puromycin was added for 10 min before washing and fixation. The Proximity Ligation Assay (PLA; Sigma) was then performed according to the manufacturer’s protocol. Cells were cytospun onto glass slides before fixation with 4% paraformaldehyde in PBS. Cells were then blocked and permeabilized with 3% BSA and 0.1% Triton X-100 for 1 hour before the addition of primary antibodies. Primary antibodies included: 1:100 anti-TOMM40 (GTX133780, Genetex) and 1:10000 mouse

anti-puromycin (MABE343, EMD Millipore). Images were acquired on a Leica SP8 confocal microscope at a magnification of 63X. Data were quantified as punctate per cell on HALO (v3.0311; Indica Labs).

Whole Cell Lysate Preparation

Lysates were prepared as previously described (55). $5x10^6$ cells were washed once with PBS and lysed in radioimmunoprecipitation assay buffer (Sigma Aldrich) on ice for 20 min. The lysate was centrifuged at 12500 rpm for 20 min at 4°C. Protein concentration of the supernatant was quantified using the Bradford Protein Assay (Bio-Rad).

Immunoblotting

As previously described (55), equal amounts of protein (whole cell lysate or isolated mitochondria) were separated on 10% SDS-PAGE gels and transferred to polyvinylidene difluoride membranes (Bio-Rad). Membranes were then blocked with 5% milk in Tris-buffered saline (Wisent) with Tween 20 (Sigma-Aldrich) (TBS-T) for 1 hour. The primary antibody in 5% milk in TBS-T was incubated with the blocked membrane overnight at 4°C. Primary antibodies included: 1:1000 LONP1 (15440-1-AP, ProteinTech), 1:2000 CLPX (ab168338, Abcam), 1:15000 TUFM (26730-1-AP, ProteinTech), 1:1000 NDUFA9 (20312-1-AP, ProteinTech), 1:1000 FLAG (F4042, Sigma Aldrich), 1:4000 β-actin (#4967, Cell Signalling), and 1:4000 MnSOD (ADI-SOD-110, Enzo). The membranes were washed and incubated with either 1:2000 secondary horseradish-conjugated donkey anti-rabbit (Cytiva, Cat# NA934) or sheep anti-mouse (Cytiva, Cat# NA931) antibodies in 5% milk in TBS-T for 1 hour at room temperature. Bio-Rad ImageLab software was used to calculate densitometry values.

Reverse Phase Protein Array

Reverse Phase Protein Array (RPPA) data sets were assembled using diagnostic samples from adult AML patients. The MD Anderson Cancer Center Institutional Review Board approved the collection protocol, research usage profile and clinical protocols that these patients were treated with. LONP1 protein expression levels of samples from patients with AML were determined using RPPA analysis. Methods and antibody validation techniques have been described previously (54).

LONP1 knockdown

Target knockdown with shRNA was conducted as previously described (55–57). The hairpin-pLKO.1 vector with scramble or LONP1 targeting shRNA sequences were isolated from glycerol stocks (Sigma Aldrich) using the E.N.Z.A. Plasmid Midi Kit (Omega Bio-tek), quantified with a NanoDrop spectrophotometer (Thermo Fisher Scientific), and validated by Sanger sequencing prior to use. Lentiviruses were produced by transfecting HEK293T cells with a three-plasmid system (pLKO.1, packaging plasmid with gag, pol, and rev genes and envelope plasmid). The target sequences of shRNAs targeting LONP1 (Accession no. NM_ 004793.4) are as follows: shLONP1 1755 5′-CCAGTGTTTGAAGAAGACCAA-3′; shLONP1 3’UTR 5′-GGACCTCAGTCGGCTTAATCA-3′ (Sigma Aldrich). $5x10^6$ cells were seeded in T25 flasks (Sarstedt) in 5 mL growth media. 5 µg/mL protamine sulfate (MP Biomedicals) and 2 mL of viral stock containing a plasmid expressing LONP1 targeting shRNA or a non-targeting control sequence were added to each flask. Following an overnight incubation, cells were resuspended in 1-5 µg/mL puromycin (Sigma Aldrich). Cells were collected for downstream experiments after 3 days. For proliferation curves of human AML cell lines,

equal numbers of live cells were plated for growth assays and counted with trypan blue exclusion staining to measure total viable cells for a period of 8 days. Growth and viability of primary AML or normal hematopoietic cells were assessed with Cell Titer Fluor 7 days after transduction as described under "Cell growth and viability assays".

For transduction of primary AML and normal cord blood cells, the shLONP1 3'UTR and scramble sequences were first cloned into hairpin-pRS19-U6-(shRNA)-UbiC-TagGFP-2A-Puro vector (Genscript) and validated by Sanger sequencing. Lentivirus were prepared as above and concentrated using Lenti-X concentrated (Takara) and resuspended in Hanks' balanced salt solution (HBSS; Gibco) supplemented with 25 mL HEPES (Fisher Scientific).

The transduction of primary AML and normal hematopoietic cord blood cells were performed as described previously (20). 6-well, non-tissue culture plates (Sarstedt) were coated with 20 µg/ml retronectin (TakaraBio) for 2 hours at room temperature. Plates were then blocked with 2% BSA in PBS (w/v) for 30 min at room temperature. After the BSA was removed, 2 mL of concentrated virus particles in HBSS supplemented with 25mM HEPES was added to each well and plates were centrifuged at 3000 rpm for 2 hours at 32°C. After centrifugation, 2x10^{6} primary AML or normal cord blood cells were added to each well in X-VIVO 10 medium supplemented with BIT, 50 ng/ml Flt3-L, 10 ng/mL IL6, 50 ng/mL SCF, 25 ng/mL TPO, 10 ng/mL IL3, 10 ng/mL G-CSF, and 5 µg/uL protamine sulfate. Plates were centrifuged at 1800 rpm at room temperature for 10 minutes to facilitate interactions between the cells and the lentivirus. Cells were then transferred to a 37°C incubator for 24 hours. Cells were resuspended in fresh X-VIVO media at a concentration of 1x10^{6} cells/ml in 6-well, non-tissue culture plates. 24 hours later,

transduction efficiency was assessed by flow cytometry and used in engraftment experiments.

LONP1 Clustered regularly interspaced short palindromic repeats (CRISPR) Knockout

LONP1 knockout with CRISPR was conducted as previously described (55). CRISPR guide oligomers were first cloned into the pLCKO vector (Addgene) and transformed into DH5α E. coli (Thermo Fisher Scientific). Plasmids containing the guides were verified by Sanger sequencing prior to lentivirus production as described previously (55). The target sequences of gRNAs targeting LONP1 (Accession no. NM_ 004793.4) are as gLONP1 #1, 5′-ACCCACAGGCGGACCTACGT-3′; gLONP1 #2 5′-TACAGGGACACTCACCGCAA-3′. OCI-AML2 cells expressing Cas9 were generated by transduction of Lenti-Cas9-2A-Blast (Addgene)-containing lentivirus and single colony expansion. $5x10^6$ OCI-AML2 expressing Cas9 cells were seeded in T25 flasks in 5 mL growth media. 5 μg/mL protamine sulfate and 2 mL of viral stock containing either a plasmid expressing a LONP1 targeting gRNA or a scramble sequence was added to each flask. Following an overnight incubation, cells were resuspended in 1.5 μg/mL puromycin. Cells were collected for downstream experiments after 14 days. For proliferation curves, equal numbers of live cells were plated for growth assays and counted with trypan blue exclusion staining to measure total viable cells for a period of 8 days.

Over-expression of wild type and mutant LONP1

Overexpression of exogenous cDNA protein was performed as previously described (56). Human wild type LONP1 cDNA ($LONP1^{wt}$) (Genscript) was subcloned into

the pLenti-EF1Fα-IRES-Blasticidin vector. FLAG-tagged mutant constructs were generated from the vector carrying the wild type LONP1 by introducing either a E591A ($LONP1^{E591A}$)or S855A ($LONP1^{S855A}$) mutation (Genscript). Plasmids were verified by Sanger sequencing prior to lentivirus production as described previously (55).

$2.5x10^6$ cells were seeded in T25 flasks in 5 mL growth media supplemented with 5 µg/mL protamine sulfate and 2 mL of viral stock containing a plasmid expressing LONP1 cDNA or a scramble control were added to each flask. Following a 48 hour incubation, cells were resuspended in growth media supplemented with 7.5 µg/mL blasticidin (Thermo Fisher) for 7 days. $5x10^6$ recovered cells were seeded in T25 flasks in 5 mL growth media. 5 µg/mL protamine sulfate and 2 mL of viral stock containing either a plasmid expressing LONP1 targeting shRNA or a scramble sequence were added to each flask. Following an overnight incubation, cells were resuspended in 1.5 µg/mL puromycin. Cells were collected for downstream experiments after 3 days. For proliferation curves, equal numbers of live cells were plated for growth assays and counted with trypan blue exclusion staining to measure total viable cells for a period of 8 days.

Cell growth and viability assays

In 96-well plates (Sarstedt), AML cell lines were plated at 12,500 cells/well while primary AML and normal hematopoietic cells were plated at 50,000 cells/well. Cells were then treated with either venetoclax (MedChemExpress), omaveloxolone (MedChemExpress) and bardoxolone methyl (MedChemExpress) in a total volume of 100 µl. After 3 days of treatment, 10 µl Alamar blue (Thermo Fisher Scientific) or 100 µl of CellTiter-Fluor (Promega) was added to each well. Plates were incubated at 37°C for 3 hours before measuring absorbance at 570 nm for Alamar blue or an excitation wavelength of 380 to

400 nm and an emission wavelength of 505 nm for Cell Titer Fluor with a SpectraMax M3 plate reader (Molecular Devices). Background fluorescence of medium-only wells was then subtracted from each reading. Viability was determined after normalizing to the DMSO control treatment.

Evaluation of cell viability by Annexin/ Propidium iodide staining

As previously described (58-59), $1x10^5$ cells were resuspended in Annexin V fluorescein isothiocyanate (FITC; Sigma) and Propidium Iodide (Biovision). Cells were analyzed by flow cytometry on a Fortessa HTS cytometer (BD Biosciences) and data was analyzed post-acquisition with FlowJo Software Version 7.7.1 (BD Biosciences).

Drug combination studies

Combinations of omaveloxolone and venetoclax were evaluated using the Excess-over-Bliss additivism (EOBA) model, as previously described (20). EOBA values were calculated using the following equation:

EOBA=C-[A+B-(A*B)]

Where A corresponds to the fractional inhibition caused by a specific concentration of drug A, B corresponds to the fractional inhibition caused by a specific concentration of drug B, and C corresponds to the fractional inhibition caused by the combination of A and B at these concentrations. Fractional inhibition was calculated using the following equation:

Fractional inhibition = 1-viability (expressed as a fraction of 1)

Positive EOBA scores represent synergistic combinations, negative EOBA scores represent antagonistic combinations, and EOBA scores of zero represent additive combinations (20).

Animal Studies

Eight- to 12-week-old male or female immunodeficient Prkdcscid 1140 /J (SCID, Jackson Laboratory) mice were used for OCI-AML2 tumor formation studies and were obtained from the University Health Network. Eight- to 12-week-old immunodeficient male or female NOD/SCID-3/GM/SF (NS-GF) were used for TEX engraftment studies or for engraftment of normal human cord blood cells, obtained from Dr. C.J. Eaves, and bred in our facility. Eight- to 12-week-old female immunodeficient NOD.Cg-Prkdc^{scid}-Il2rg^{tm1Wjl}/SzJ (NSG, Jackson Laboratory) mice were used for engraftment of primary human AML cells and were obtained from the University Health Network.

Mice were assigned randomly to each experimental group. During all experiments, the weights of the mice were approximately 18 to 30 g, with no animals losing more than 10% body weight. All animals were housed in microisolator cages with temperature-controlled conditions under a 12-hour light/dark cycle with access to drinking water and food. Only one experimental procedure was performed on each mouse, and all mice were drug naïve prior to experiments. All animal studies were performed in accordance with the University Health Network Animal Use Protocol No. 1251 for NS-GF, SCID, and NSG.

As previously described (55), $1x10^6$ OCI-AML2 cells were transduced with shRNA in lentiviral vectors targeting LONP1 or a control sequence were injected subcutaneously into the right flanks of SCID mice. Mice were euthanized after approximately 3 weeks

when the diameter of the tumor reached 1.5 cm. To assess survival, mice were sacrificed once tumors reached 1.5 cm or on d40, whichever event occurred first.

Alternatively, $1x10^6$ OCI-AML2 cells were injected subcutaneously into the right flanks of SCID mice, as previously mentioned (58). Once tumors were palpable, the mice were treated with either 7.5 mg/kg omaveloxolone or barodoxolone methyl or vehicle (5% DMSO, 12% Cremophor (Sigma Aldrich), 8% ethanol (Sigma Aldrich), 75% saline (Thermo Fisher Scientific)) intraperitoneally three times weekly. Mice were euthanized after approximately 3 weeks when the diameter of the tumor reached 1.5 cm.

Tumor volumes were measured three times per week based on caliper measurements of tumor length, width, and height (volume = tumor length × width × height × 0.52). After euthanasia, tumor volumes and mass were measured from excised tumors.

As previously described (20), $2x10^5$ TEX cells transduced with shRNA in lentiviral vectors targeting LONP1 or a control sequence were injected into the right femur of sub-lethally irradiated NS-GF mice. After 6 weeks, mice were euthanized, and the percentage of human CD45+ (BD Biosciences) in the left femur was enumerated in TEX cells by flow cytometry.

Alternatively, $2x10^5$ TEX cells were injected into the right femur of sub-lethally irradiated NS-GF mice. One week after injection, mice were treated with either 7.5 mg/kg omaveloxolone or barodoxolone methyl or vehicle intraperitoneally three times weekly (n = 5 per group). After 6 weeks, mice were euthanized, and the percentage of human CD45+ in the left femur was enumerated in TEX cells by flow cytometry.

To assess omaveloxolone in murine models of engraftment, $1.8x10^6$ primary AML cells were injected into the right femur of 10-week-old female NSG mice which have previously been irradiated with 2 Gy of a 137-Cs source. As described previously (20), $1.6x10^6$ normal human cord blood cells (Stem Cell Technologies) were injected into the right femur of NS-GF mice. Mice were treated with a vehicle control or 7.5 mg/kg omaveloxolone (by intraperitoneal injection). Mice were euthanized, and cells were flushed from the femurs with a 26-gauge needle. Engraftment of human cells into the marrow of the non-injected left femur was assessed by enumerating the percentage of cells stained for CD45+ CD33+ (BD Biosciences).

To assess the effects of omaveloxolone on protein aggregation in AML cells xenografted in mice, mice were treated with omaveloxolone daily for 6 days. After treatment, human CD45 cells were isolated from the mouse marrow using sorted using EasySep™ Mouse/Human Chimera Isolation Kit # 19849 (StemCell Technologies). Isolated cells were then stained with proteostat and imaged with confocal microscopy.

The engraftment of transduced primary AML and normal hematopoietic cord blood cells were performed as described previously (20). $2.6x10^6$ transduced primary AML cells or $1.6x10^6$ normal cord blood cells were injected into the right femur of NSG mice. After 12 weeks, engraftment of human cells into the marrow of the non-injected left femur was assessed by enumerating the percentage of GFP+CD45+CD33+ cells.

Cell enumeration for all engraftment experiments used the Cytoflex flow cytometer (Beckman Coulter) and data was analyzed with FlowJo version 7.7.1. Engraftment potential was measured as described previously (20). The transduction efficiency of primary AML samples and normal cord blood was measured by assessing the percentage

of GFP positive cells by flow cytometry. After sacrificing the mice, the percentage of CD33+ CD45+ cells were measured and relative engraftment was calculated by the formula published earlier. All *in vivo* studies were carried out adhering to the regulations of the Canadian Council on Animal Care and with the approval of the University Health Network Ethics Review Board.

Recombinant LONP1 Production

Wild type, E591A, or S855A LONP1 cDNA with an N-terminal His_6-tag was cloned into a pET28a-TEV expression vector (Genscript) and plasmids were verified by Sanger sequencing prior to recombinant protein production. Plasmids were transformed into BL21-CodonPlus (DE3)-RIPL E. coli (Agilent) for expression. E. coli were cultured in Terrific broth medium (Wisent) supplemented with 50 µg/mL kanamycin (Sigma Aldrich), 50 µg/mL streptomycin (Sigma Aldrich) and 25 µg/mL chloramphenicol (Sigma Aldrich) in a shaking incubator at 37 °C to an optical density of 0.6. Protein expression was induced with 0.5 mM isopropyl β-D-1-thiogalactopyranoside (Sigma Aldrich) for 20 hours at 18 ºC. Cells were harvested and stored at -70°C for later use. Cells were lysed in lysis buffer (50 mM Tris pH 7.5, 300 mM NaCl (Sigma Aldrich), 10% [v/v] glycerol (Sigma Aldrich), 20 mM imidazole (Sigma Aldrich), 2 mM 2-mercaptoethanol (Sigma Aldrich), and 1X Halt™ Protease Inhibitor Cocktail (Thermo Fisher Scientific). The lysate was loaded onto a pre-equilibrated Ni-NTA column (Cytiva) to allow His_6-LONP1 binding. Then, the column was washed sequentially by wash buffer (50 mM Tris pH 7.5, 700 mM NaCl, 10% [v/v] glycerol, 0.02% [v/v] Triton X-100, 2 mM 2-mercaptoethanol) containing 80 mM imidazole to remove non-specifically bounded proteins. LONP1 was eluted from the column with elution buffer (25 mM Tris, pH 7.5, 300 mM NaCl, 10% [v/v] glycerol, 500 mM imidazole,

2 mM 2-mercaptoethanol). Fractions containing LONP1 were pooled and dialyzed against 50 mM Tris pH 7.5, 200 mM KCl, 25 mM $MgCl_2$, 10% [v/v] glycerol, 1 mM DL-dithiothreitol (Sigma Aldrich) at 4ºC overnight using a dialysis membrane (Thermo Fisher Scientific). Purified LONP1 protein was concentrated, quantified by BCA protein assay, then stored at -70°C.

Measurement of LONP1 ATPase activity

LONP1 ATPase activity was determined by ATP hydrolysis using the Malachite Green Phosphate Assay Kit (BioAssay System) as per manufacturer instructions. 0.5 μM LONP1 was incubated in assay buffer (50 mM Tris pH 7.5, 200 mM KCl, 25 mM $MgCl_2$, 10% [v/v] glycerol, 1 mM DL-dithiothreitol) supplemented with either a DMSO control, ATPɣS (Sigma), bortezomib, omaveloxolone, or bardoxolone methyl for 1 hour at 37°C. 2.5 mM ATP (Sigma Aldrich) was added to initiate the reaction. After 1 hour, malachite green reagent was added to halt the reaction and absorbance was measured at 620nm to quantify the phosphate released from ATP hydrolysis with a SpectraMax M3 plate reader.

Measurement of LONP1 proteolytic activity

0.5 μM recombinant LONP1 was incubated for 1 hour at 37 °C in a reaction buffer (50 mM Tris pH 7.5, 200 mM KCl, 25 mM $MgCl_2$, 10% [v/v] glycerol, 1 mM DL-dithiothreitol) supplemented with 2.4 μM FITC-Casein (Sigma Aldrich) and either a DMSO control, ATPɣS, bortezomib, omaveloxolone, or bardoxolone methyl for 1 hour at 37°C. 2.5 mM ATP was added to initiate the reaction. The release of free FITC molecules was monitored over time at Ex/Em of 485/535 nm with a SpectraMax M3 plate reader pre-warmed to 37°C. The rate of LONP1 protease activity was determined by calculating the linear slope of the degradation curve.

Clonogenic growth assays

Clonogenic growth assays were performed as previously described (55). Primary AML or normal hematopoietic cells were plated in MethoCult H4434 medium (StemCell Technologies) in the presence of either a DMSO control or 250 nM omaveloxolone or 250 nM bardoxolone methyl at $1x10^5$ cells/ml on 35-mm dishes (Nunclon). After incubating for 10-14 days, the number of colonies containing 50 or more cells on normal samples or 8 or more cells on AML primary samples was counted on an inverted microscope. The mean of the triplicate plates for each condition is presented.

Mitochondrial protein aggregation assay

$2x10^6$ cells were spun down onto glass slides using a cytospin centrifuge. Cells on glass slides were fixed with 4% paraformaldehyde (Electron Microscopy) in PBS and then permeabilized with 3% BSA in PBS with 0.1% Triton X-100 for 1 hour. Cells were stained with 1:400 rabbit anti-TOMM20 antibody (42406, Cell Signalling Technologies) for 1 hour, 1:800 goat anti-rabbit IgG conjugated with Alexa Fluor® 488 (Jackson Immunoresearch) for 1 hour, proteostat (Enzo) for 30 min, and 5 µg/mL 4′,6-diamidino-2-phenylindole (DAPI; Thermo Fisher) for 5 min. Images were acquired on a Leica SP8 confocal microscope at 63X magnification. Image analyses were conducted with HALO (v3.0311; Indica Labs) or ImageJ.

Mitochondrial isolation from cultured cells

As previously described (60), $2\text{-}4x10^7$ cells were washed in phosphate-buffered saline (pH 7.4) (PBS, Gibco), resuspended in cold mitochondrial isolation buffer [10 mM Tris/MOPS (pH 7.2) (Sigma Aldrich), 200 mM sucrose (Sigma Aldrich), and 1 mM

EGTA/Tris (pH 7.2)(Sigma Alrich) with 1X protease inhibitors (Thermo Fisher Scientific) and transferred to a pre-chilled glass Dounce homogenizer. Cells were homogenized until 10% of cells were viable as assessed by trypan blue exclusion staining. The lysate was then centrifuged at 600g for 10 min at 4°C. The supernatant was centrifuged at 7000g for 10 min at 4°C. The pellet was washed twice in cold mitochondrial isolation buffer to reduce cytoplasmic contamination. The pellet was resuspended in cold mitochondrial isolation buffer for protein quantification using the Pierce BCA Protein Assay Kit (Thermo Fisher Scientific). Isolated mitochondria were then used for immunoblotting or the mitochondrial protein solubility assay.

Mitochondrial protein solubility assay

As previously described (55), isolated mitochondria were resuspended in cold mitochondrial isolation buffer supplemented with 1% Triton X-100 (Sigma Aldrich) for 30 minutes at 4°C. Permeabilized mitochondria were centrifuged for 10 min at 20,000 g at 4°C to separate insoluble pellet and soluble supernatant fractions. Fractions were resuspended in equal volumes and used for immunoblotting.

Seahorse metabolic flux analysis

Seahorse metabolic flux analysis was preformed as described previously (57). OCI-AML2 were washed and resuspended in unbuffered alpha-MEM medium (Agilent) and then seeded at $1x10^5$ cells/well in XF24 plates (Agilent) coated with 0.15 mg/well Cell-Tak (Corning). Cells were equilibrated in the unbuffered alpha-MEM medium supplemented with 2% FBS for 1 hour in a 37°C, CO_2-free incubator before being transferred to the XF24 analyzer. The oxygen consumption rate was measured with sequential injections of 0.25 μM oligomycin (Sigma Aldrich), and 0.75 μM carbonyl cyanide p-

trifluoromethoxyphenylhydrazone (FCCP; Sigma Aldrich), and 1.25 µM of Antimycin A and rotenone (Sigma Aldrich).

Mitochondrial superoxide measurement

As previously described (57), $1x10^5$ cells were stained with 5 µM MitoSOX (Thermo Fisher) and incubated in the dark for 30 min at 37°C. Cells were centrifuged to remove the dye, resuspended in 0.2 mL binding buffer supplemented with Annexin V–FITC (BioLegend), and analyzed by flow cytometry on a Fortessa HTS cytometer. The percentages of Annexin V–negative and MitoSOX-positive cells were determined, and the fold change in ROS production was calculated. Data was analyzed post-acquisition with FlowJo Software Version 7.7.1 (BD Biosciences).

Mitochondrial isolation from normal organs

As previously described (60), harvested organs were suspended in ice-cold mitochondrial isolation buffer with 1X Halt™ protease inhibitor cocktail. 0.5 mg tissue was minced on ice with a sharp razor and was then transferred to a pre-chilled glass mortar in 2 mL isolation buffer. The tissue was homogenized 3-4 times at 1600 rpm. The homogenate was then centrifuged at 600g for 10 min at 4°C. The supernatant was centrifuged at 7000g for 10 min at 4°C. The pellet was washed twice in cold mitochondrial isolation buffer to reduce cytoplasmic contamination. The pellet was resuspended in cold mitochondrial isolation buffer for the mitochondrial protein solubility assay.

Supplementary figures S1-S22

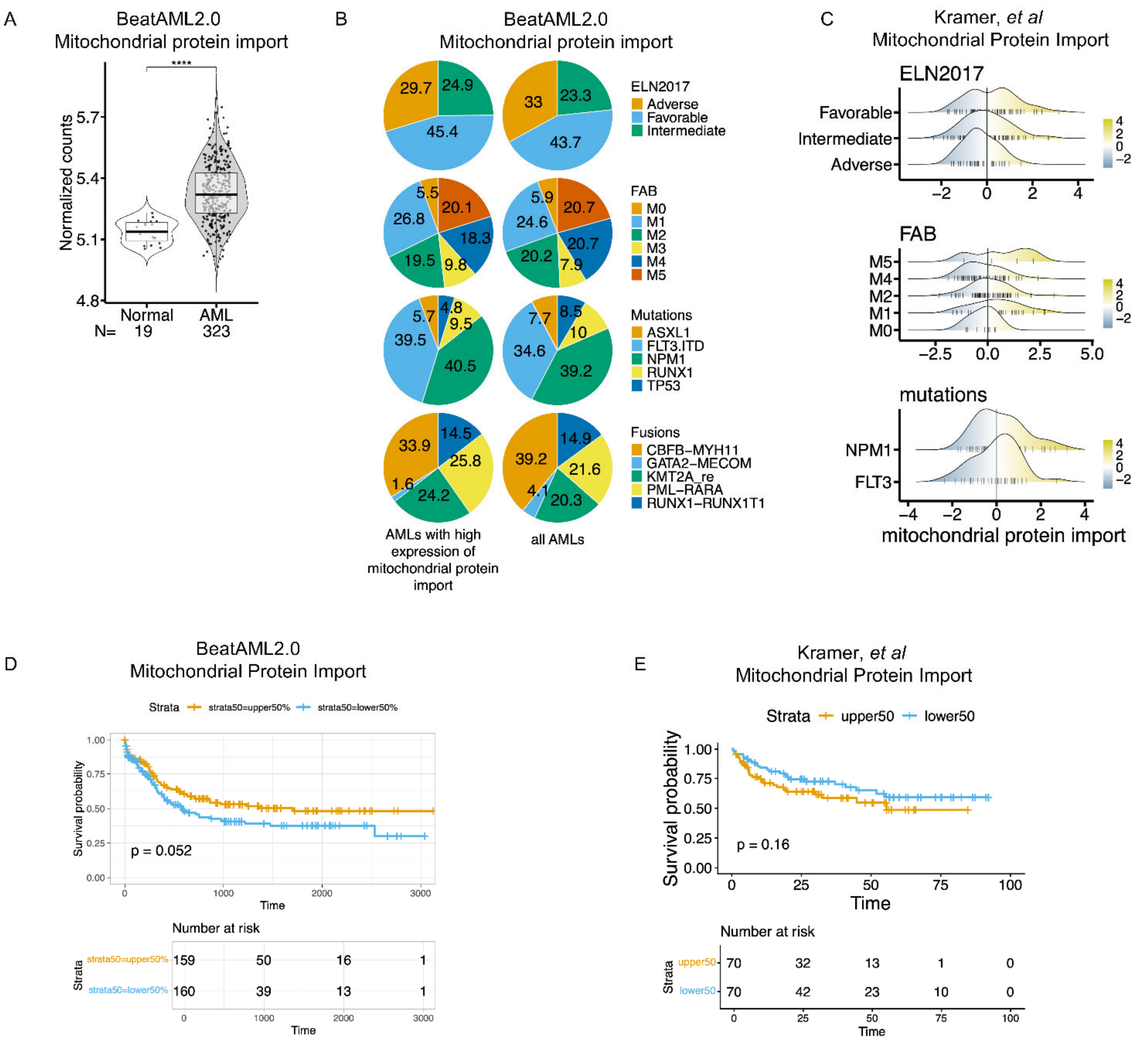

Figure S1

Figure S1. Mitochondrial protein import expression across AML ELN2017 risk groups, cytogenetic subtypes, and FAB subgroups.

(A) Violin plot of mitochondrial protein import gene expression (GO 0072655: establishment of protein localization to mitochondrion) in primary *de novo* AML and normal mononuclear bone marrow hematopoietic cells from BeatAML2.0 (52). The

midline represents the median value for each group. ****$p=7.6*10^{-15}$. Statistical analyses were performed using an unpaired, two-tailed Student's t-test.

(B) Profile of AMLs with higher expression of mitochondrial protein import (Beat AML2.0 (52)) in comparison with the profile of all AMLs stratified by ELN2017 risk, FAB classification, and recurrent fusions and mutations. No significant differences among groups using a Fisher's exact test.

(C) Density plots show normalized protein expression of mitochondrial protein import in the proteomic dataset Kramer (53) stratified by ELN2017 risk, FAB classification, and recurrent mutations. Color indicates z-score (blue = low, yellow = high). No significant differences among groups using a Wilcoxon rank sum test.

(D) Kaplan–Meier curves of overall survival in AML patients stratified by high (upper 50%) versus low (lower 50%) expression of mitochondrial protein import in BeatAML2.0 (52). Log-rank p-values shown; tables indicate numbers at risk. p= 0.052.

(E) Kaplan–Meier curves of overall survival in AML patients stratified by high (upper 50%) versus low (lower 50%) protein expression of mitochondrial protein import in the Kramer dataset (53). Log-rank p-values shown; tables indicate numbers at risk. p= 0.16.

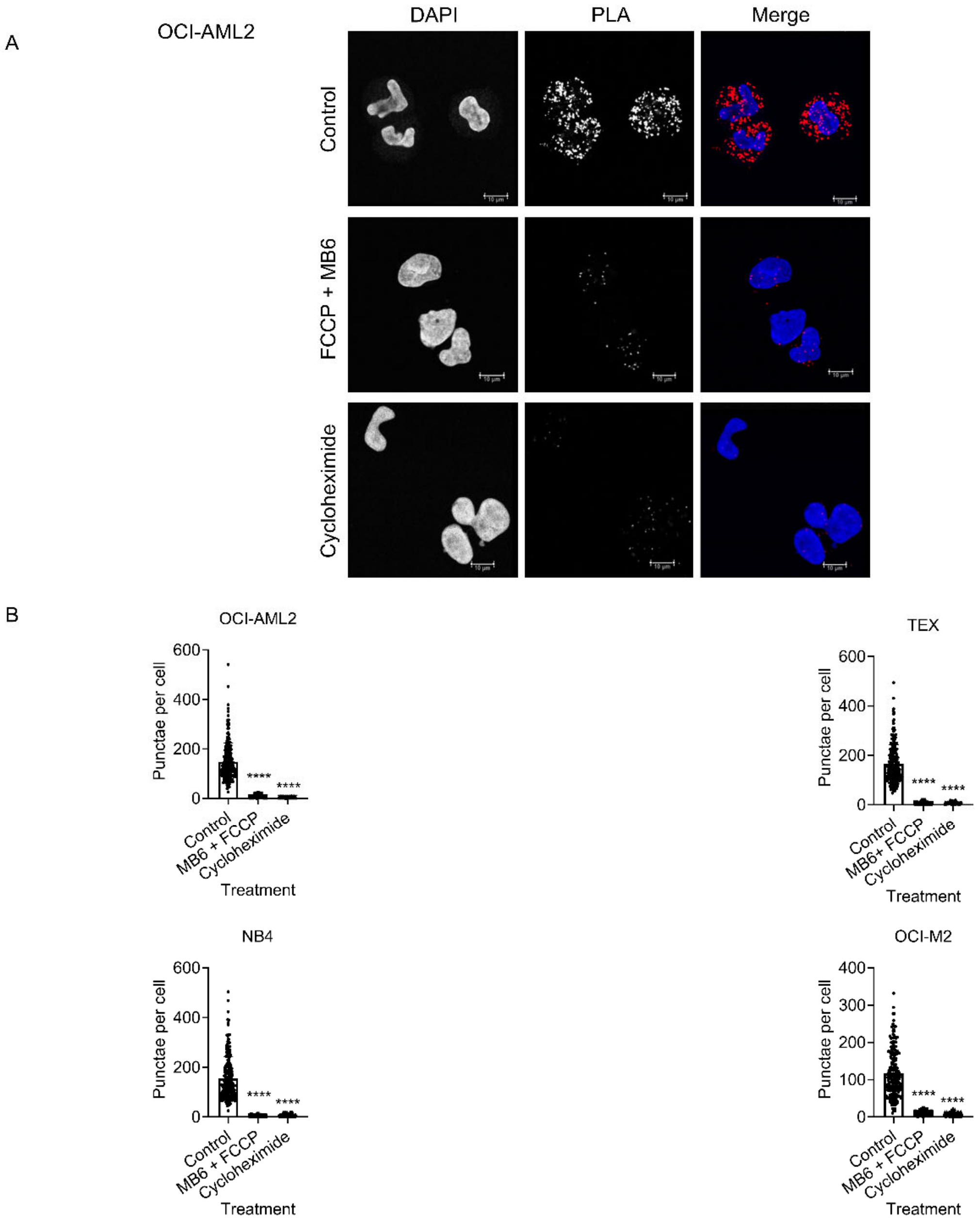

Figure S2

Figure S2. Mitochondrial protein import in OCI-AML2, TEX, NB4, OCI-M2.

(A) OCI-AML2 cells were treated with 50 μM Mitoblock-6 (MB6) and 5 μM carbonyl cyanide-p-trifluoromethoxyphenylhydrazone (FCCP) or 50 μM cycloheximide. After 5 hours, cells were treated with puromycin (1 μg/mL puromycin) for 7.5 minutes. Cells were washed, fixed and stained with anti-puromycin, anti-TOMM40 (mitochondria) antibodies

and DAPI (nucleus). Co-localization of puromycin and TOMM40 was detected by a PLA assay and cells were imaged by confocal microscopy. Representative cells are shown. (B) Mean ± SD PLA punctae per cell in OCI-AML2, TEX, NB4, and OCI-M2 cells treated in (A), quantified using HALO Image Analysis software (n=102-218 cells per sample). ****p<0.0001 by a one-way ANOVA with Dunnett's multiple comparisons test.

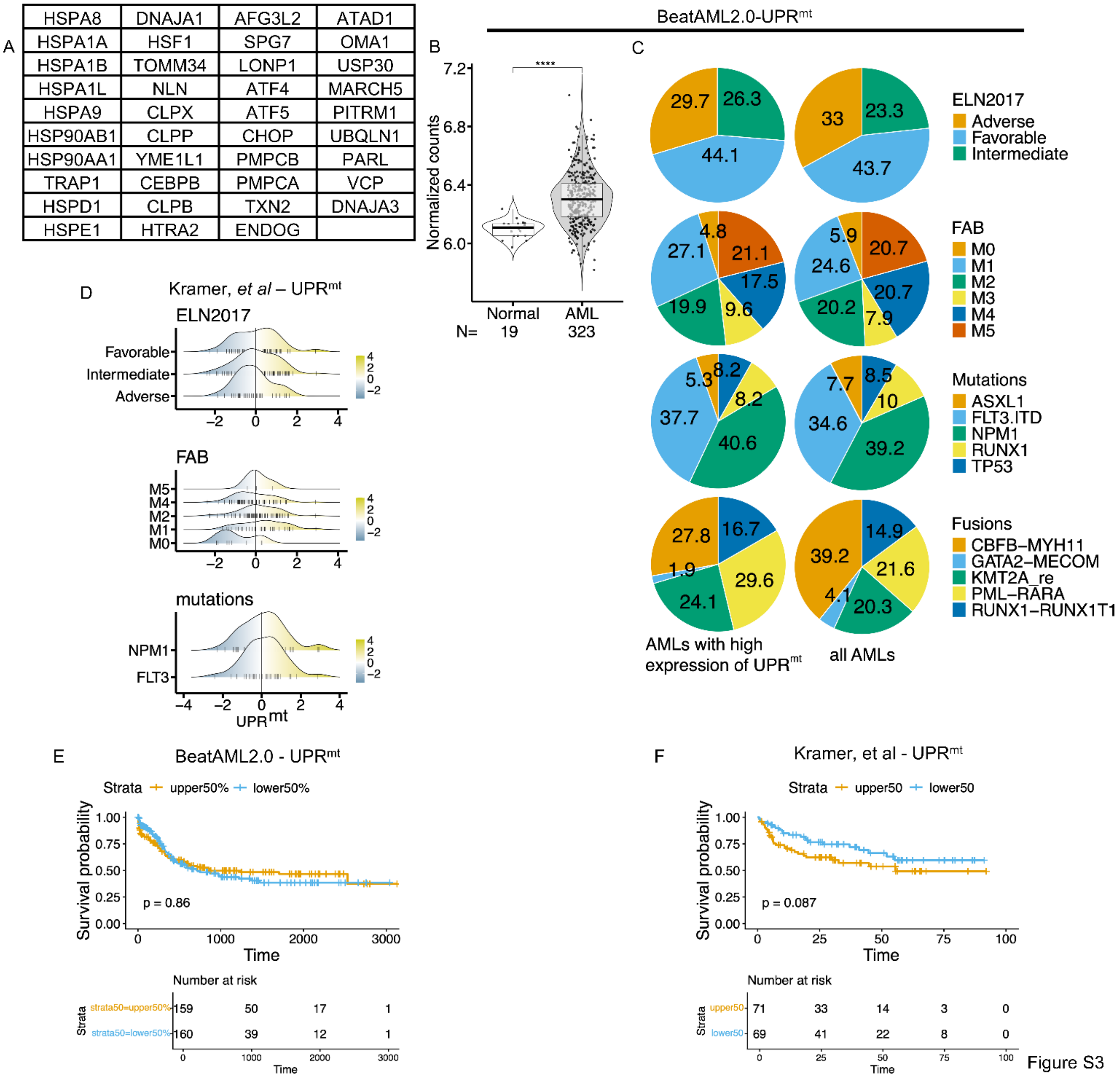

Figure S3

Figure S3. The human UPR^{mt} signature is increased in primary AML samples, compared to normal adult hematopoietic samples, and equally expressed across ELN2017 risk groups, AML cytogenetic subtypes and FAB subgroups.

(A) List of 39 genes comprising the human UPR^{mt}. Refer to Supplementary Table 1 for literature sources.

(B) Violin plot of UPR^{mt} expression in primary *de novo* AML and normal adult mononuclear bone marrow hematopoietic cells from BeatAML2.0 (52). The midline represents the median value for each group. ****$p<0.0001$. Statistical analyses were performed using an unpaired, two tailed Student's t-test.

(C) Profile of AMLs with higher UPR^{mt} expression (Beat AML2.0 (52)) in comparison with the profile of all AMLs stratified by ELN2017 risk, FAB classification, and recurrent fusions and mutations. No significant differences among groups using a Fisher's exact test.

(D) Density plots show normalized UPR^{mt} protein expression in the proteomic dataset Kramer stratified by ELN2017 risk, FAB classification, and recurrent mutations (53). Color indicates z-score (blue = low, yellow = high). No significant differences among groups using a Wilcoxon rank sum test.

(E) Kaplan–Meier curves of overall survival in AML patients stratified by high (upper 50%) versus low (lower 50%) expression of UPR^{mt} genes in BeatAML2.0 (52). Log-rank p-values shown; tables indicate numbers at risk. p= 0.86.

(F) Kaplan–Meier curves of overall survival in AML patients stratified by high (upper 50%) versus low (lower 50%) UPR^{mt} protein expression in the Kramer dataset (53). Log-rank p-values shown; tables indicate numbers at risk. p= 0.087.

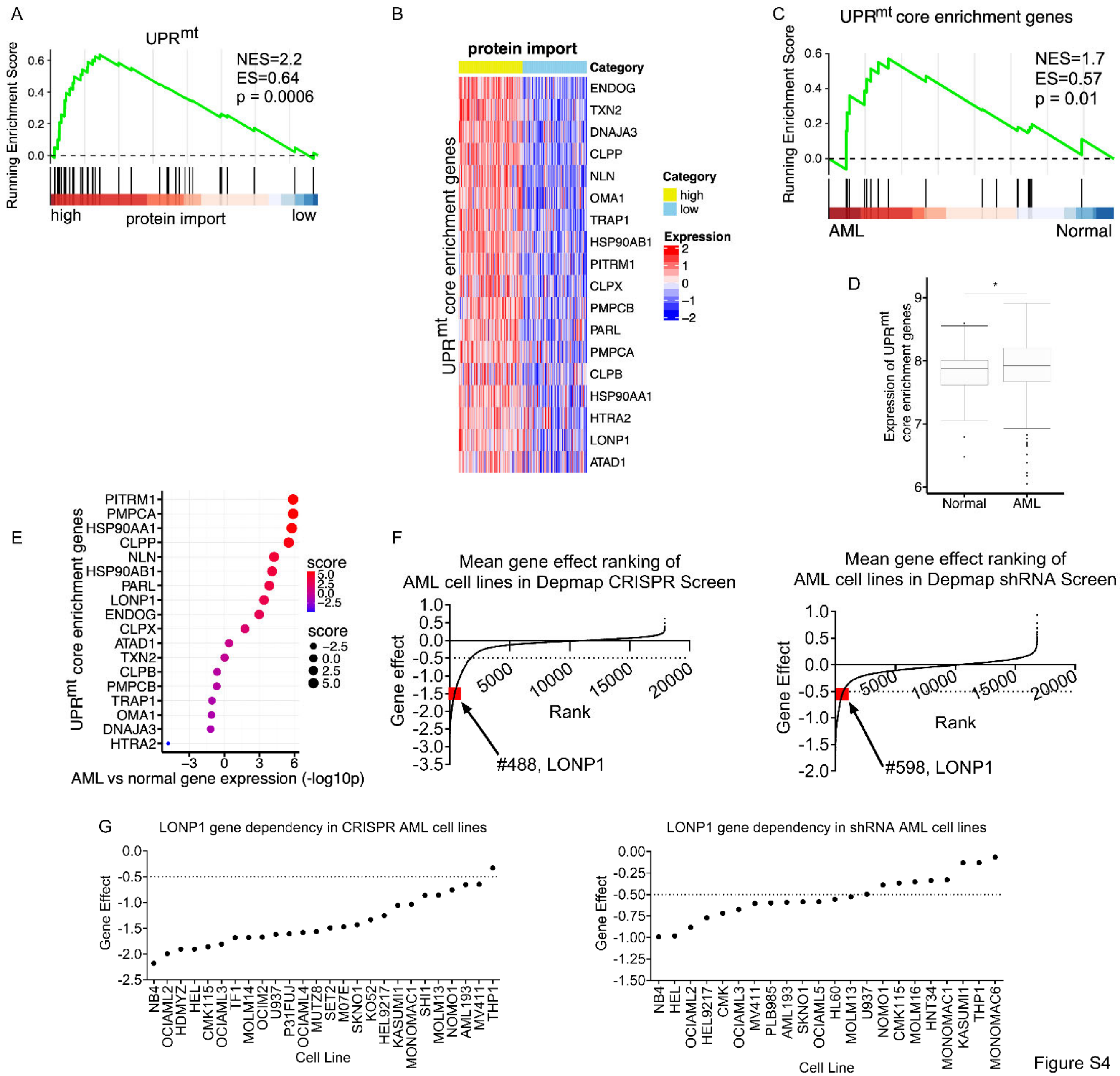

Figure S4. LONP1 is a top UPR^{mt} essential gene in CRISPR and shRNA Depmap screens of AML cell lines.

(A) Leading edge analysis was conducted on UPR^{mt} genes in primary AML cells stratified by protein import gene expression from GSE13159.

(B) Expression heat map of 18 UPR^{mt} core enrichment genes identified by leading edge analysis in (A) of primary AML samples (n=542) from GSE13159 stratified by mitochondrial protein import gene expression.

(C) Leading edge analysis was conducted on UPR^{mt} genes in primary AML and normal mononuclear bone marrow hematopoietic cells from GSE13159.

(D) Mean ± SD expression of the core set of 18 UPR^{mt} genes identified in (A) in primary AML and normal hematopoietic bone marrow samples from GSE13159. $^*p=1.9*10^{-2}$. Statistical analyses were performed using an unpaired, two-tailed Student's t-test.

(E) Expression of each of the 18 core UPR^{mt} genes identified in (A) in primary AML, compared to normal hematopoietic bone marrow samples from GSE13159.

(F) Ranked plot of average gene effect scores of all genes across 26 and 22 AML cell lines in CRISPR and shRNA DepMap screens, respectively. Gene effect values below -0.5 (dashed line) indicates gene essentiality in AML. (DepMap Public 24Q2+Score, Chronos, Achilles+DRIVE+Marcotte, DEMETER2).

(G) LONP1 gene effect scores across 26 and 22 AML cells lines in CRISPR and shRNA DepMap screens, respectively. Gene effect values below -0.5 (dashed line) indicates gene essentiality in AML. (DepMap Public 24Q2+Score, Chronos, Achilles+DRIVE+Marcotte, DEMETER2).

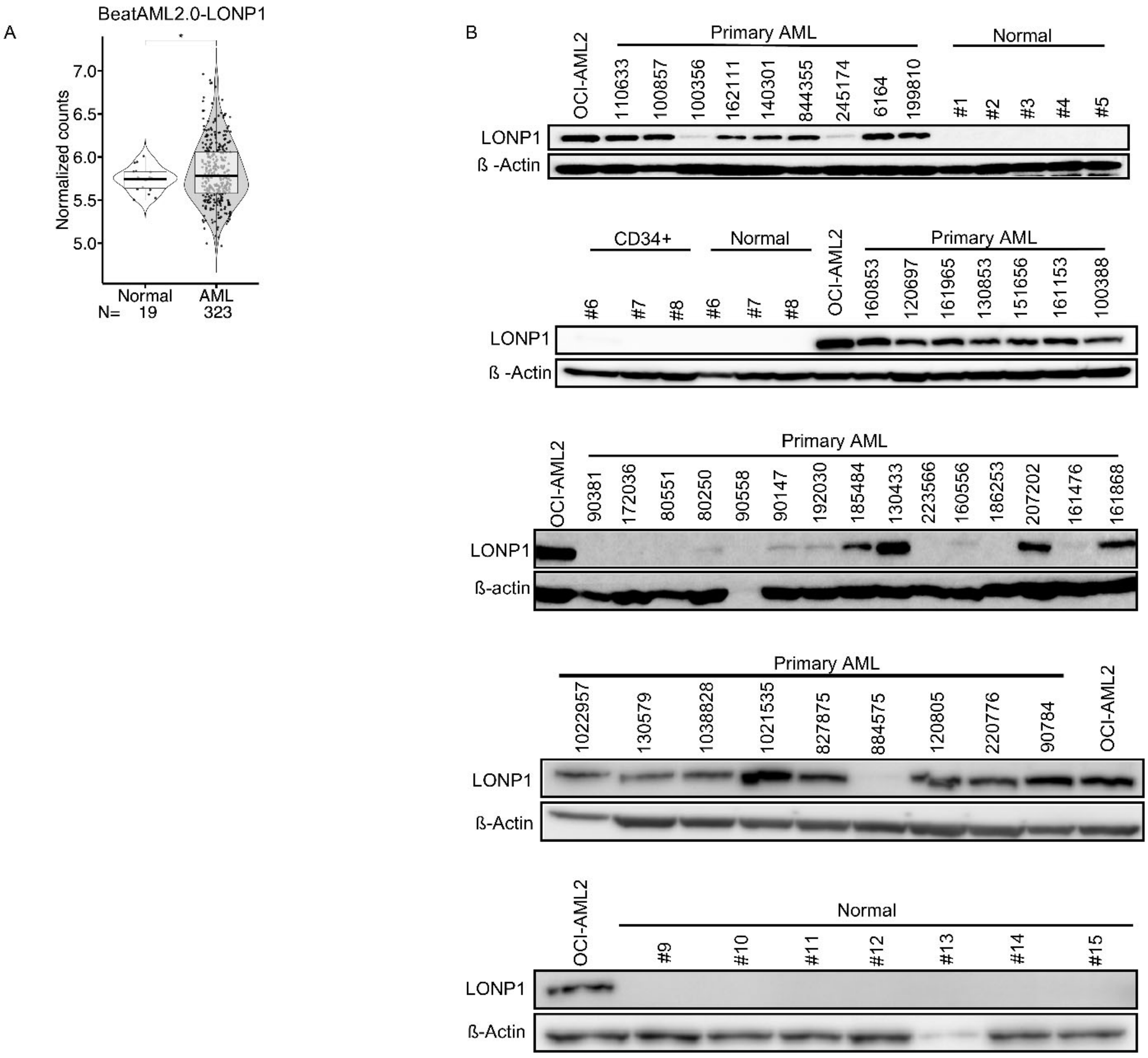

Figure S5

Figure S5. LONP1 mRNA and protein expression are increased in primary AML samples, compared to normal adult hematopoietic samples.

(A) Violin plot of LONP1 gene expression in primary *de novo* AML and normal mononuclear bone marrow hematopoietic cells from BeatAML2.0 (52). The midline represents the median value for each group. *p=0.039. Statistical analyses were performed using an unpaired, two-tailed Student's t-test.

(B) Expression of LONP1 protein in primary AML (n=39), normal adult hematopoietic cells (n=14) and CD34+ progenitors (n=3) was measured in cell lysates by immunoblotting. Expression was quantified by densitometry and displayed relative to LONP1 protein levels in OCI-AML2 cells. Primary AML 90558 and normal #13 were omitted from densitometry analysis due to low β-actin signal. See Supplementary table 2 (Patient cytogenetics).

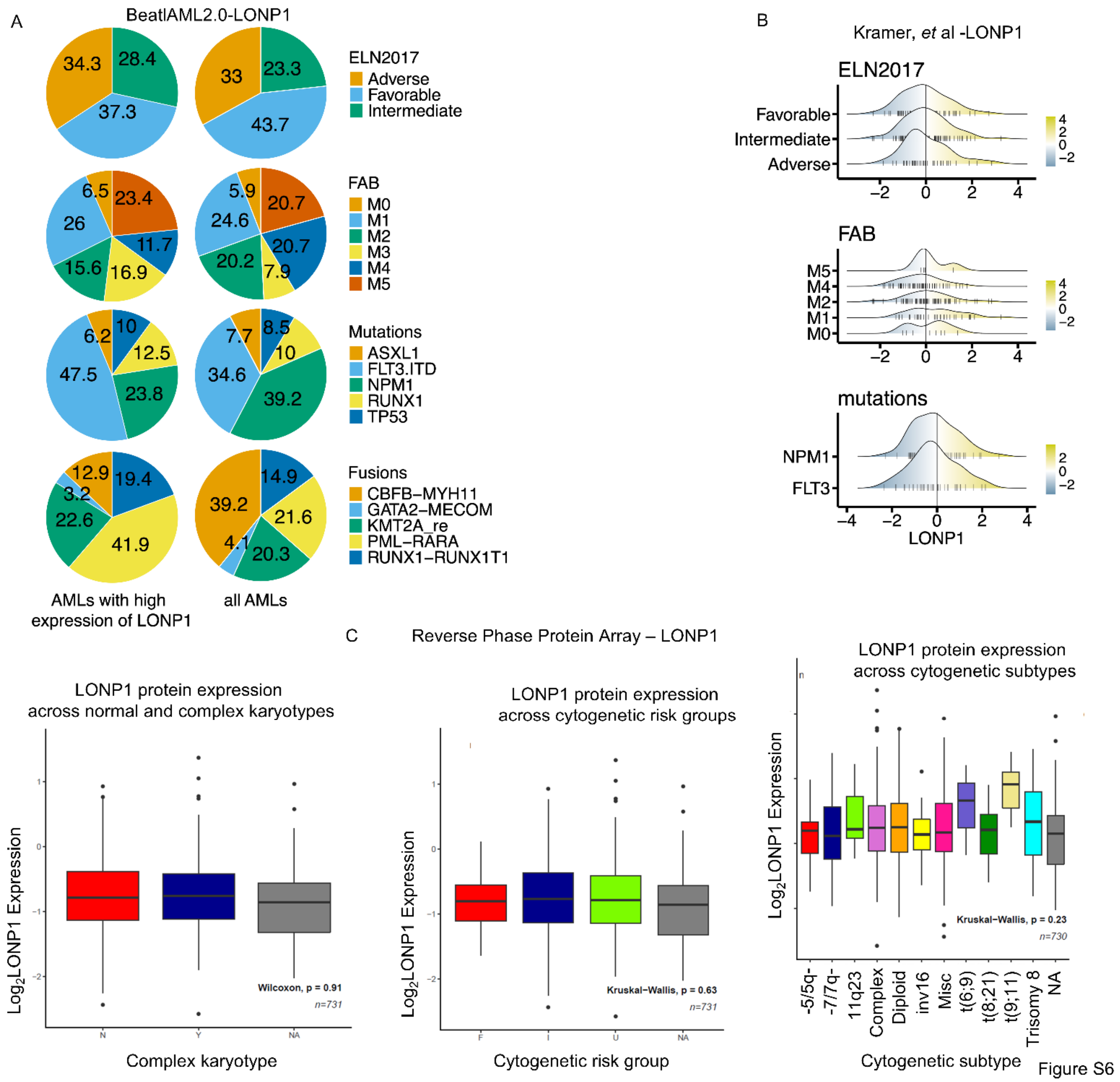

Figure S6

Figure S6. LONP1 mRNA and protein are equally expressed across AML risk groups, cytogenetic subtypes and karyotype subgroups.

(A) Profile of AMLs with higher expression of LONP1 (Beat AML2.0 (52)) in comparison with the profile of all AMLs stratified by ELN2017 risk, FAB classification, and recurrent

fusions and mutations. No significant differences among groups using a Fisher's exact test.

(B) Density plots show normalized protein expression of LONP1 in the proteomic dataset Kramer stratified by ELN2017 risk, FAB classification, and recurrent mutations (53). Color indicates z-score (blue = low, yellow = high). No significant differences among groups using a Wilcoxon rank sum test.

(C) Mean ± SD LONP1 protein expression across primary AML (n=818) stratified by karyotype status, cytogenetic risk groups, and cytogenetic subtypes as assessed by reverse phase protein array (RPPA). n.s $p>0.05$ by a one-way ANOVA with Kruskal-Wallis test.

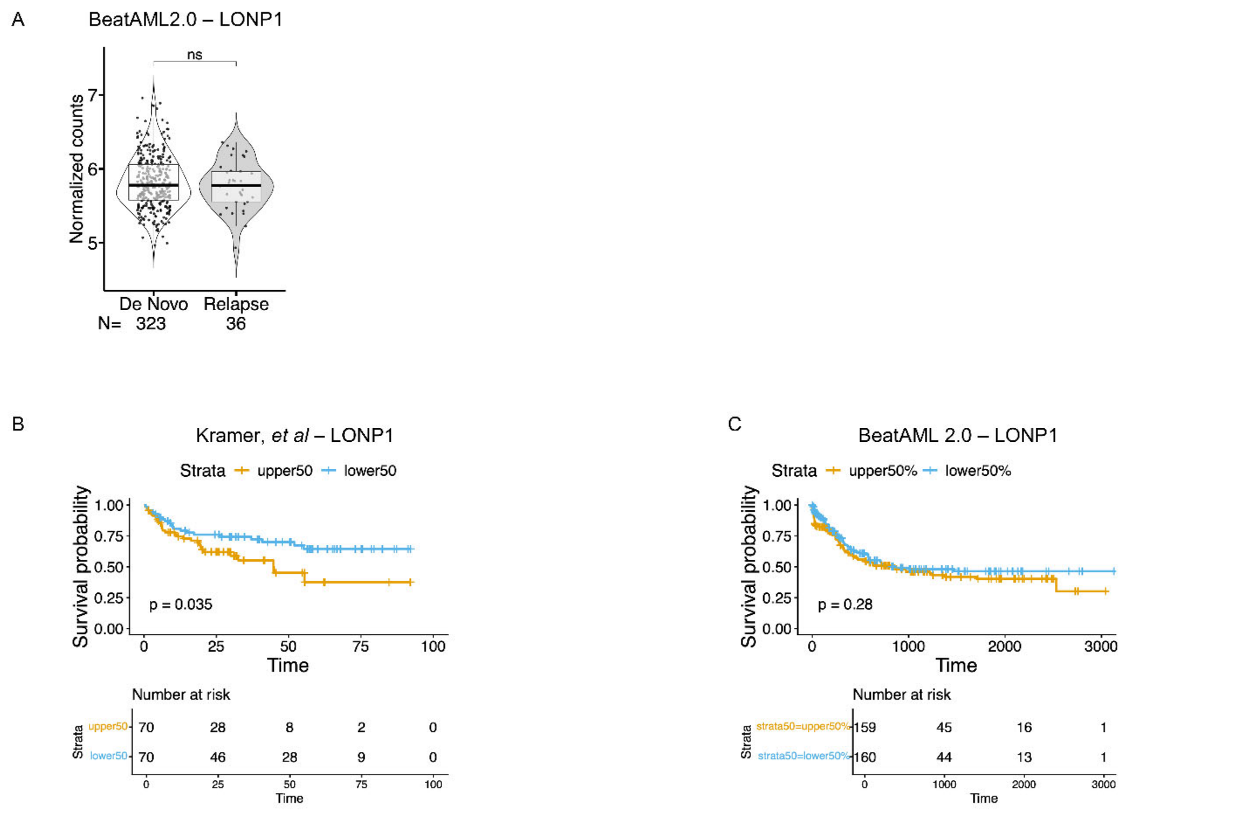

Figure S7

Figure S7. LONP1 mRNA is equally expressed across de novo and relapsed AML samples.

(A) Violin plot of LONP1 gene expression in relapsed and *de novo* AML from BeatAML2.0 (52). The midline represents the median value for each group. ns: p=0.43. Statistical analyses were performed using an unpaired, two-tailed Student's t-test.

(B) Kaplan–Meier curves of overall survival in AML patients stratified by high (upper 50%) versus low (lower 50%) protein expression of LONP1 in the proteomic dataset Kramer (53). Log-rank p-values shown; tables indicate numbers at risk. *p= 0.035.

(C) Kaplan–Meier curves of overall survival in AML patients stratified by high (upper 50%) versus low (lower 50%) mRNA expression of LONP1 in BeatAML2.0 (52). Log-rank p-values shown; tables indicate numbers at risk. ns p= 0.28.

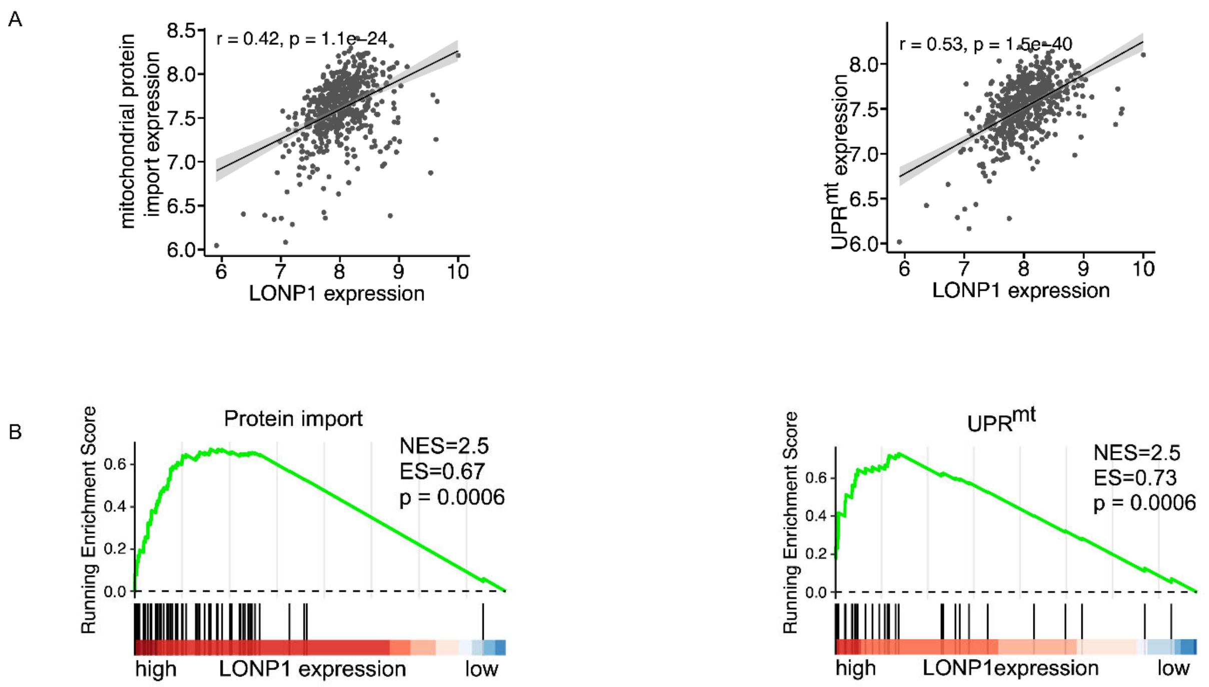

Figure S8

Figure S8. LONP1 mRNA expression is positively correlated with expression of mitochondrial protein import and UPR^{mt} genes.

(A) Correlation between LONP1 gene expression vs. mitochondrial protein import (GO 0072655: establishment of protein localization to mitochondrion) and the UPR^{mt} gene set expression in primary AML samples from GSE13159.

(B) Enrichment plots of mitochondrial protein import (GO 0072655: establishment of protein localization to mitochondrion) and UPR^{mt} genes in primary AML from GSE13159 stratified by LONP1 expression.

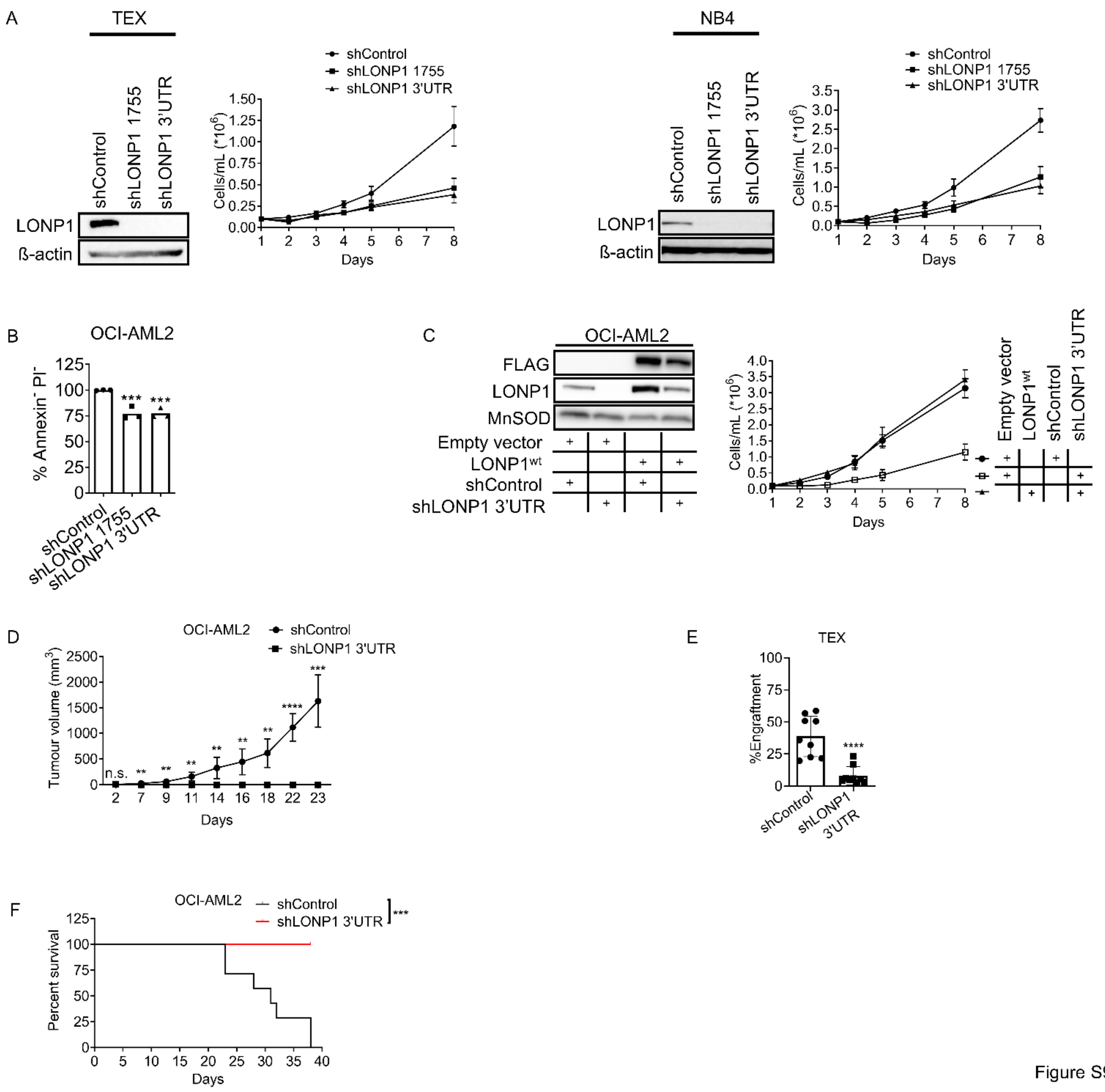

Figure S9

Figure S9. LONP1 knockdown is cytotoxic to AML cells.

(A) Mean ± SD growth and viability by trypan blue staining of TEX and NB4 leukemia cells transduced with shRNA targeting LONP1 or control sequences. Expression of LONP1 or β-actin 7 days after transduction was measured by immunoblotting. Representative data from n=3 biological replicates are shown.

(B) OCI-AML2 cells were transduced with shRNA targeting LONP1 or control sequences. 7 days after transduction, mean ± SD cell viability was assessed by annexin V/propidium iodide staining and flow cytometry (n=3 technical replicates). ***p=0.0001 by a one-way ANOVA with Dunnett's multiple comparison test.

(C) OCI-AML2 cells were transduced with empty vector or FLAG-tagged wild type LONP1 cDNA. 14 days later, cells were transduced with shRNA targeting the 3'UTR of endogenous LONP1 or control sequences. 7 days after transduction, levels of FLAG-tagged and total LONP1, as well as MnSOD protein were measured in mitochondrial lysates. Mean ± SD growth and viability was measured by trypan blue staining of transduced OCI-AML2 cells. Representative data from n=3 biological replicates are shown.

(D) OCI-AML2 cells were transduced with shRNA targeting LONP1 or control sequences. Equal cell numbers were injected subcutaneously into the flanks of SCID mice (n=5 per group). Mean ± SD tumor volumes were measured. Day #: 2 (p=0.5447), 7 (**p=0.0013), 9 (**p=0.0034), 11 (**p=0.0025), 14 (**p=0.0078), 16 (**p=0.0041), 18 (**p=0.0012), 22 (****p<0.0001), Day 23 (***p=0.0001) using unpaired, two-tailed Student's t-tests.

(E) TEX cells were transduced with shRNA targeting LONP1 or control sequences. Equal cell numbers were injected into the right femurs of NS-GF mice (n=9 per group). After 6 weeks, mice were sacrificed, cells flushed from left femurs and engraftment measured with anti-human CD45 antibodies. ****p<0.0001 by an unpaired, two-tailed Student's t-test.

(F) OCI-AML2 cells were transduced with shRNA targeting LONP1 or control sequences. Equal numbers of viable cells were injected subcutaneously into the flanks of SCID mice (n=5 per group). Survival was measured over time. ***p=0.0002 by a log-rank test.

A

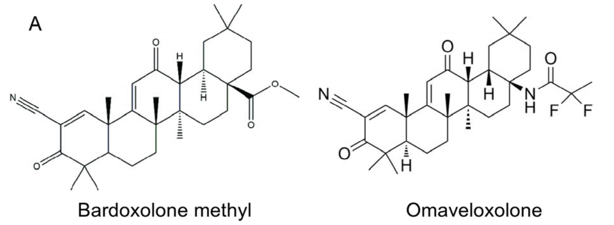

B

IC50 (μM)	Omaveloxolone	Bardoxolone Methyl
ATPase activity	0.79 ± 0.61	1.33 ± 0.70
Proteolytic activity	1.32 ± 0.62	1.08 ± 0.31

C

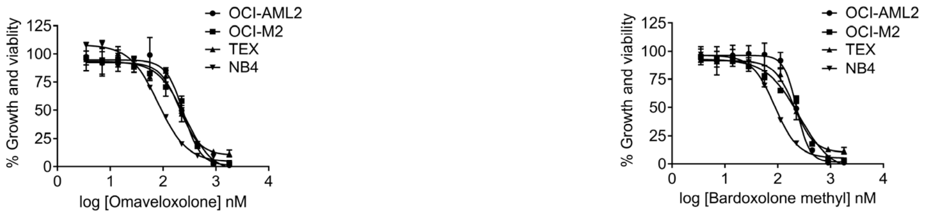

D

72 hr IC50 (nM)	OCI-AML2	OCI-M2	TEX	NB4
Omaveloxolone	243.3±78.7	259.5±81.3	206.2±76.8	80.77±19.4
Bardoxolone methyl	228.9± 71.8	253.1±79.6	204.9±71.4	88.52±19.1

E

Excess over bliss scores		Omaveloxolone (nM)			
		353.5	388.28	426.5	472.5
Venetoclax (μM)	2.47	0.35	0.24	0.21	0.19
	4.22	0.32	0.21	0.18	0.16
	7.22	0.19	0.12	0.10	0.09
	12.94	0.03	0.02	0.02	0.01

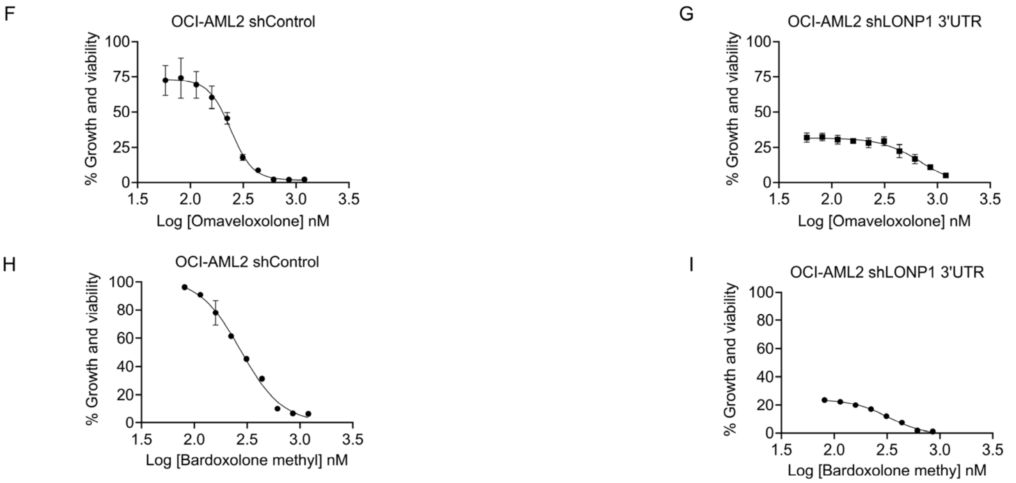

Figure S10

Figure S10. Omaveloxolone and bardoxolone methyl inhibit LONP1 ATPase and proteolytic activities and suppress the growth of OCI-AML2, OCI-M2, TEX, and NB4 cells.

(A) Chemical structures of bardoxolone methyl and omaveloxolone.

(B) Recombinant LONP1 protein was treated with increasing concentrations of omaveloxolone or bardoxolone methyl. ATPase and proteolytic activity were measured. Data represent the mean ± SD IC_{50} from n=3 replicates.

(C) OCI-AML2, OCI-M2, TEX, and NB4 cells were treated with increasing concentrations of omaveloxolone or bardoxolone methyl for 72 hours. Mean ± SD growth and viability was measured with Cell Titer Fluor from n=3 replicates.

(D) Table summarizing mean ± SD growth and viability values for OCI-AML2, OCI-M2, TEX, and NB4 from (C).

(E) OCI-AML2 cells were treated with increasing concentrations of omaveloxolone, or venetoclax, or a combination for 72 hours. Growth and viability was assessed by Alamar blue (n=3 replicates) and drug-drug interaction was calculated using Excess-over-Bliss additivism (EOBA) model. Positive EOBA scores indicate synergism and negative EOBA scores indicated antagonism.

(F) OCI-AML2 cells were transduced with shRNA targeting control sequences. 7 days post transduction, cells were treated with increasing concentrations of omaveloxolone for 72 hours. Mean ± SD growth and viability was assessed by Cell Titer Fluor (n=3 replicates).

(G) OCI-AML2 cells were transduced with a shRNA targeting the LONP1 3'UTR. 7 days post transduction, cells were treated with increasing concentrations of omaveloxolone for 72 hours. Mean ± SD growth and viability was assessed by Cell Titer Fluor (n=3 replicates).

(H) OCI-AML2 cells were transduced with shRNA targeting control sequences. 7 days post transduction, cells were treated with increasing concentrations of bardoxolone

methyl for 72 hours. Mean ± SD growth and viability was assessed by Cell Titer Fluor (n=3 replicates).

(I) OCI-AML2 cells were transduced with a shRNA targeting the LONP1 3'UTR. 7 days post transduction, cells were treated with increasing concentrations of bardoxolone methyl for 72 hours. Mean ± SD growth and viability was assessed by Cell Titer Fluor (n=3 replicates).

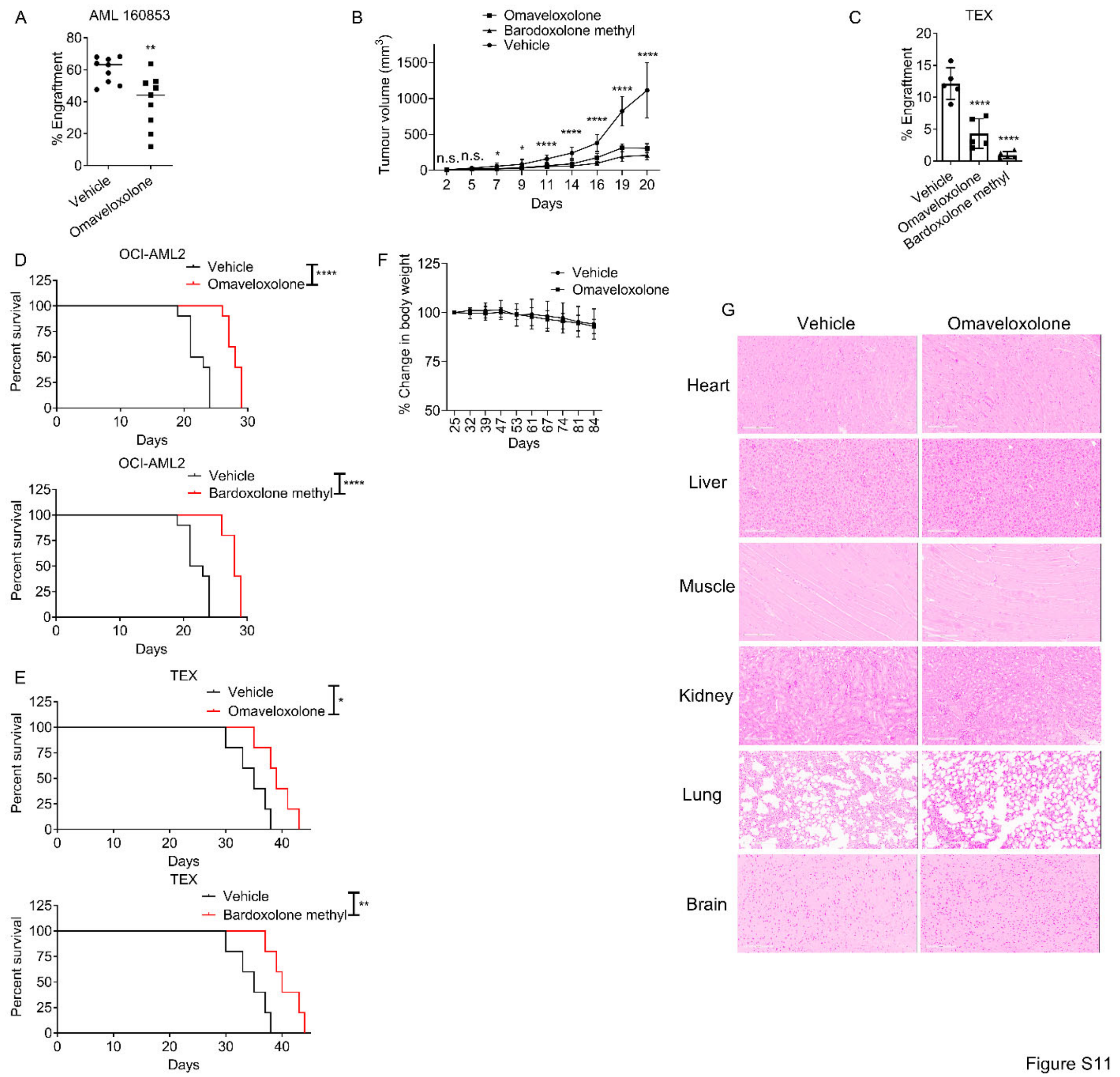

Figure S11. Omaveloxolone reduces leukemic burden and extends survival of mice with AML xenografts.

(A) Primary AML cells were injected into the right femurs of sub-lethally irradiated NSG mice. 4 week post injection, mice were treated with 7.5 mg/kg omaveloxolone or vehicle for 8 weeks (n=9 mice per group). Engraftment of primary AML cells in the un-injected left femur was assessed by flow cytometry with anti-human CD45 and CD33 antibodies.

**p=0.0058. Statistical analyses were performed using an unpaired, two-tailed Student's t-test. See Supplementary table 2 (Patient cytogenetics).

(B) OCI-AML2 cells were injected subcutaneously into the flanks of SCID mice. When tumors were palpable, mice were treated intraperitoneally with omaveloxolone or bardoxolone methyl (7.5 mg/kg 3x/week). Mean ± SD tumor growth was measured over time. Day 2 (omaveloxolone: n.s., p=0.3637; bardoxolone methyl: n.s., 0.7659), Day 5 (omaveloxolone: n.s., p=0.0518; bardoxolone methyl: *p=0.0168), Day 7 (omaveloxolone: *p=0.0343; bardoxolone methyl: *p=0.0116), Day 9 (omaveloxolone: *p=0.205; bardoxolone methyl: *p=0.0138), Day 11 (omaveloxolone: ****p<0.0001; bardoxolone methyl: ****p<0.0001), Day 14 (omaveloxolone: ****p<0.0001; bardoxolone methyl: ****p<0.0001), Day 16 (omaveloxolone: ****p<0.0001; bardoxolone methyl: ****p<0.0001), Day 19 (omaveloxolone: ****p<0.0001; bardoxolone methyl: ****p<0.0001), Day 20 (omaveloxolone: ****p<0.0001; bardoxolone methyl: ****p<0.0001). Statistical analyses were performed using a one-way ANOVA with Dunnett's multiple comparisons test.

(C) TEX cells were injected into the right femur of NS-GF mice. 1 week post injection, mice were treated intraperitoneally with omaveloxolone or bardoxolone methyl (7.5 mg/kg 3x/week). 6 weeks post injection, engraftment of TEX cells into the left femur was measured by flow cytometry with anti-human CD45 antibody. Vehicle vs omaveloxolone (****p<0.0001), vehicle vs. bardoxolone methyl (****p<0.0001). Statistical analyses were performed using a one-way ANOVA with Dunnett's multiple comparisons test.

(D) OCI-AML2 cells were injected subcutaneously into the flanks of SCID mice. When tumors became palpable, mice were treated intraperitoneally with omaveloxolone or

bardoxolone methyl (7.5 mg/kg 3x/week). Survival was measured over time. ****$p<0.0001$ by a log-rank test.

(E) TEX were injected into the right femurs of NS-GF mice. One week post injection, mice received omaveloxolone or bardoxolone methyl intraperitoneally (7.5 mg/kg 3x/week) (n=5 mice per group). Survival was measured over time. Vehicle vs omaveloxolone (*$p<0.0275$); Vehicle vs bardoxolone methyl (**$p<0.0088$). Statistical analyses were performed with a log-rank test.

(F) Normal hematopoietic cord blood cells were injected into the right femurs of sub-lethally irradiated NS-GF mice. 4 weeks post-injection, mice were treated intraperitoneally with omaveloxolone or vehicle (7.5 mg/kg 3x/week) and weight was measured over time (n=9-10 mice per group). ns $p>0.05$.

(G) Representative organ histological images of heart, liver, muscle kidney, lung, and brain from mice treated with omaveloxolone or vehicle in (C). Scale bars: 200 µm.

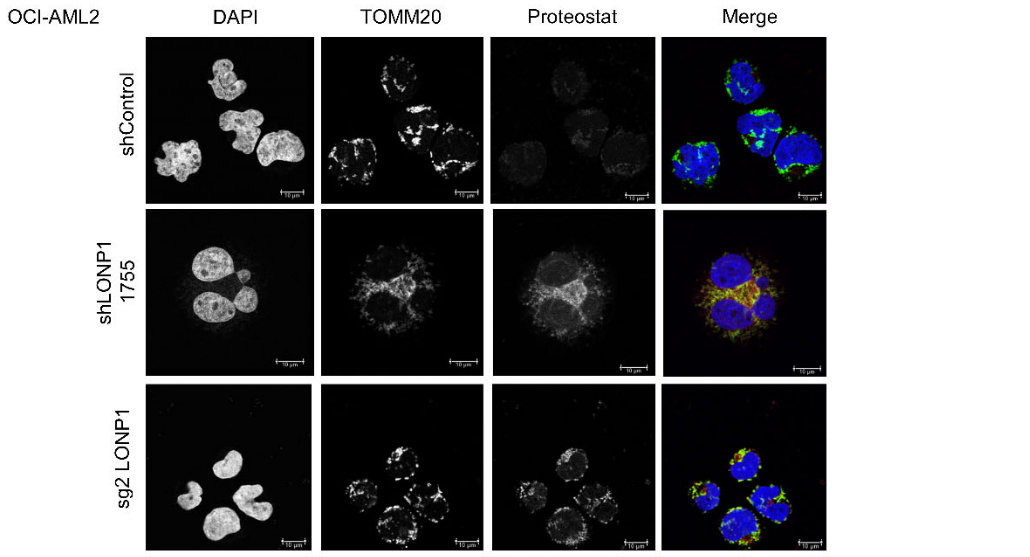

Figure S12. Genetic inhibition of LONP1 increased mitochondrial protein aggregation in OCI-AML2 cells.

OCI-AML2 cells were transduced with shRNA or gRNA targeting LONP1 or control sequences. 7 (shRNA) or 14 (gRNA) days after transduction cells were stained with proteostat to detect aggregated proteins, FITC-anti-TOMM20 (mitochondria) and DAPI (nucleus). Cells were imaged by confocal microscopy. Representative cells are shown.

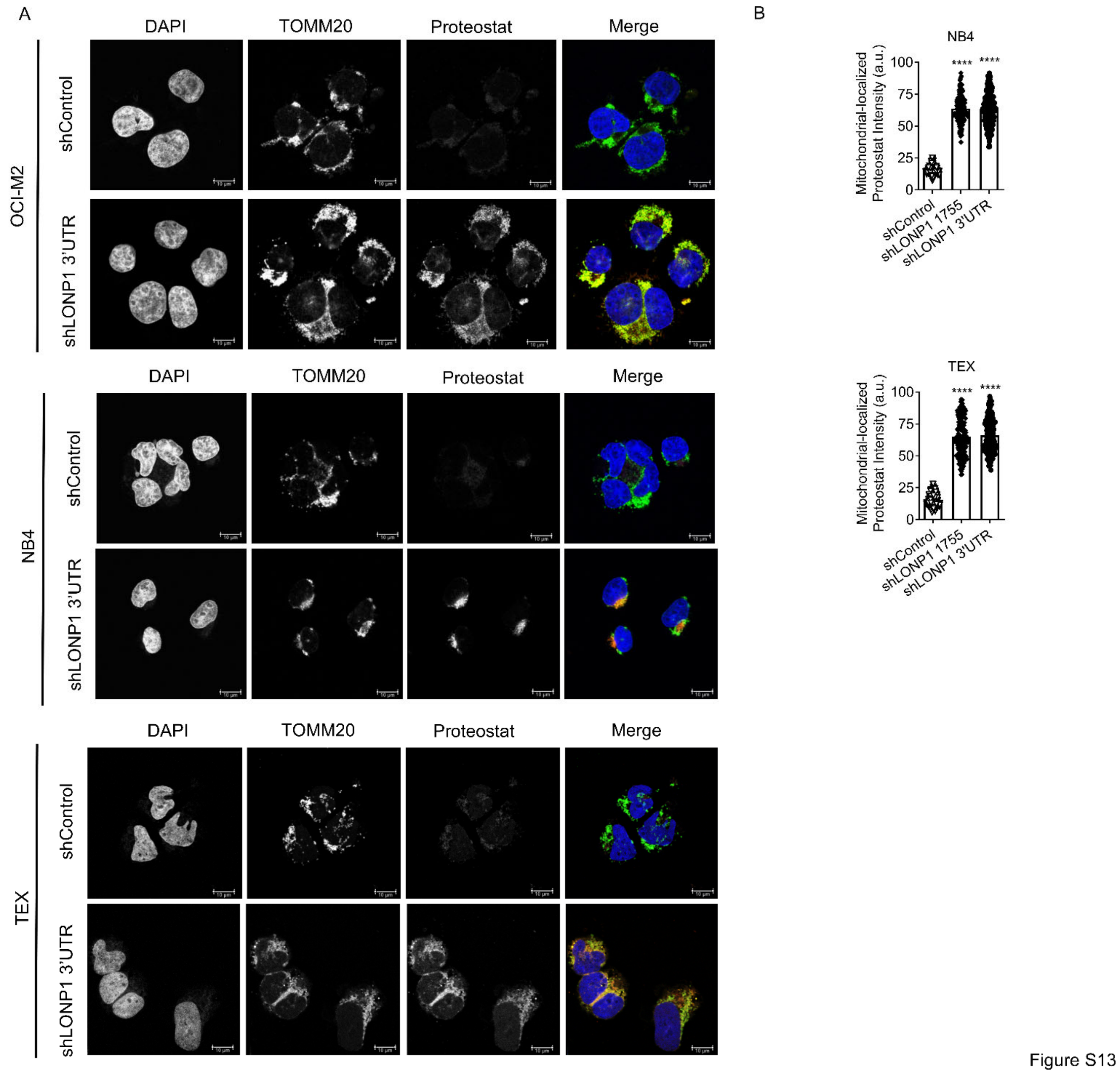

Figure S13. Genetic inhibition of LONP1 increases mitochondrial protein aggregation in OCI-M2, NB4, and TEX cells.

(A) OCI-M2, NB4, and TEX cells were transduced with shRNA targeting LONP1 or control sequences. 7 days after transduction, mitochondrial protein aggregation was measured as described in Figure S12. Representative cells are shown.

(B) NB4 and TEX cells were transduced with shRNA targeting LONP1 or control sequences. 7 days later, mitochondrial protein aggregation was measured as described in Figure S12. Colocalization of proteostat with TOMM20 was quantified by HALO Image analysis software (n=164-205 cells per group). ****p<0.0001 by a one-way ANOVA with Dunnett’s multiple comparison test.

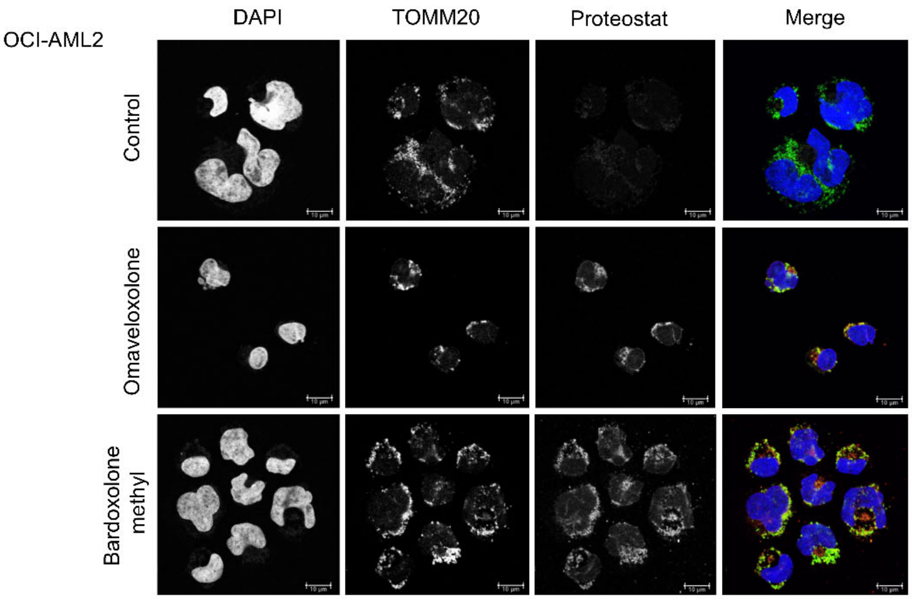

Figure S14

Figure S14. Omaveloxolone and bardoxolone methyl increase mitochondrial protein aggregation in OCI-AML2 cells.

OCI-AML2 cells were treated with 250 nM omaveloxolone or bardoxolone methyl for 30 hours. After treatment, mitochondrial protein aggregation was measured as described in Figure S12. Representative cells are shown.

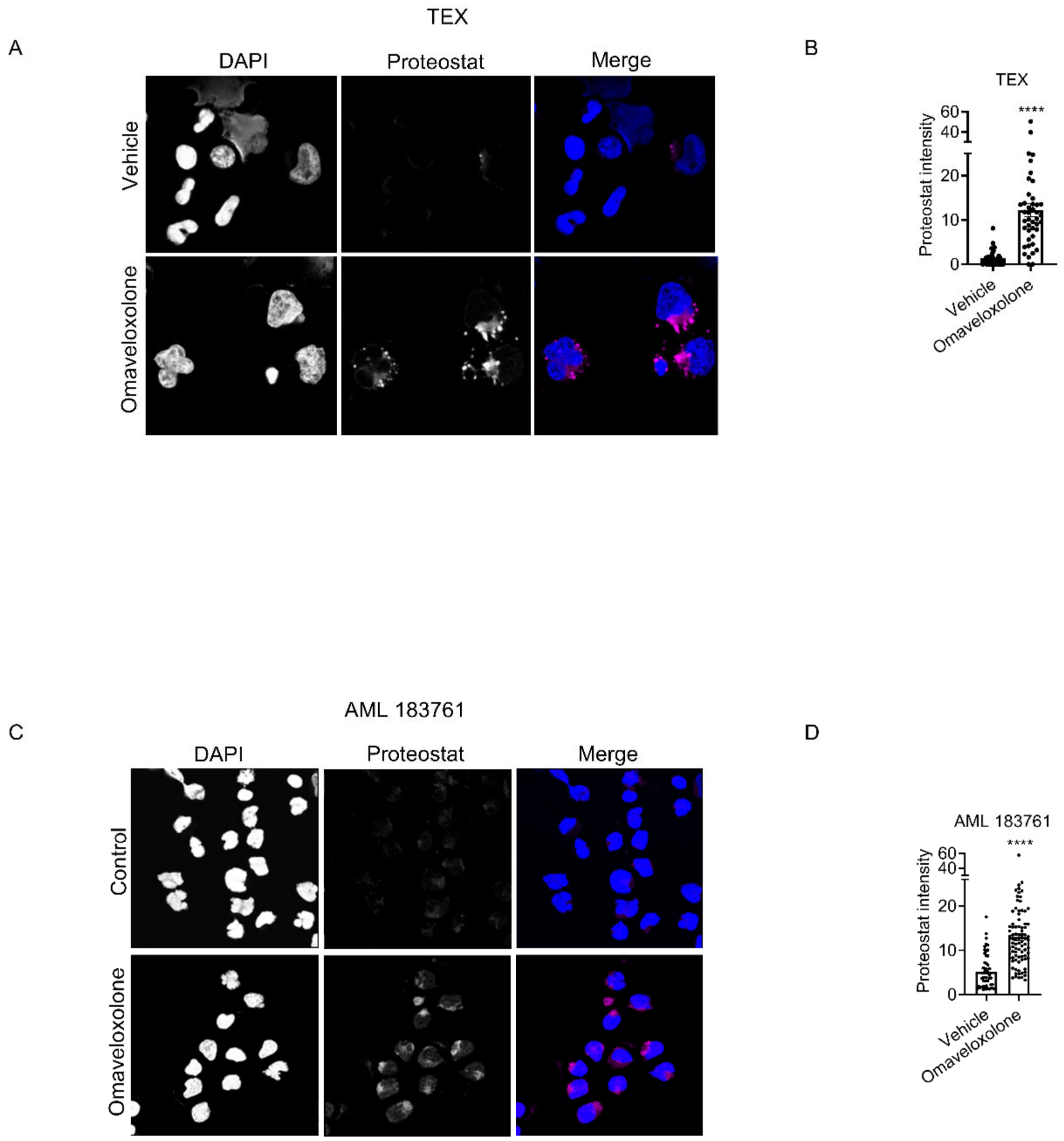

Figure S15

Figure S15. Systemic treatment with Omaveloxolone induces mitochondrial protein aggregation in engrafted TEX and primary AML cells.

(A) TEX cells were injected into the right femur of NSG-F mice. 1 week post injection, mice were treated intraperitoneally with omaveloxolone (7.5 mg/kg) daily for 6 days. Post

treatment bone marrow cells were harvested from femurs; sorted the human CD45 cells and stained with proteostat to detect aggregated proteins. Cells were imaged by confocal microscopy. Representative images are shown.

(B) Quantification of proteostat fluorescence intensity in samples from (A), calculated using ImageJ. Data are presented as mean ± SEM; statistical significance was determined by unpaired, two-tailed t-tests (****$p \leq 0.0001$).

(C) CD45+ cells from AML 183761 in Fig. 3F were sorted and stained with proteostat to detect aggregated proteins followed by confocal microscopy imaging. Representative images are shown.

(D) Quantification of proteostat fluorescence intensity in samples from (C), calculated using ImageJ. Data are presented as mean ± SEM; statistical significance was determined by unpaired, two-tailed t-tests (****$p \leq 0.0001$).

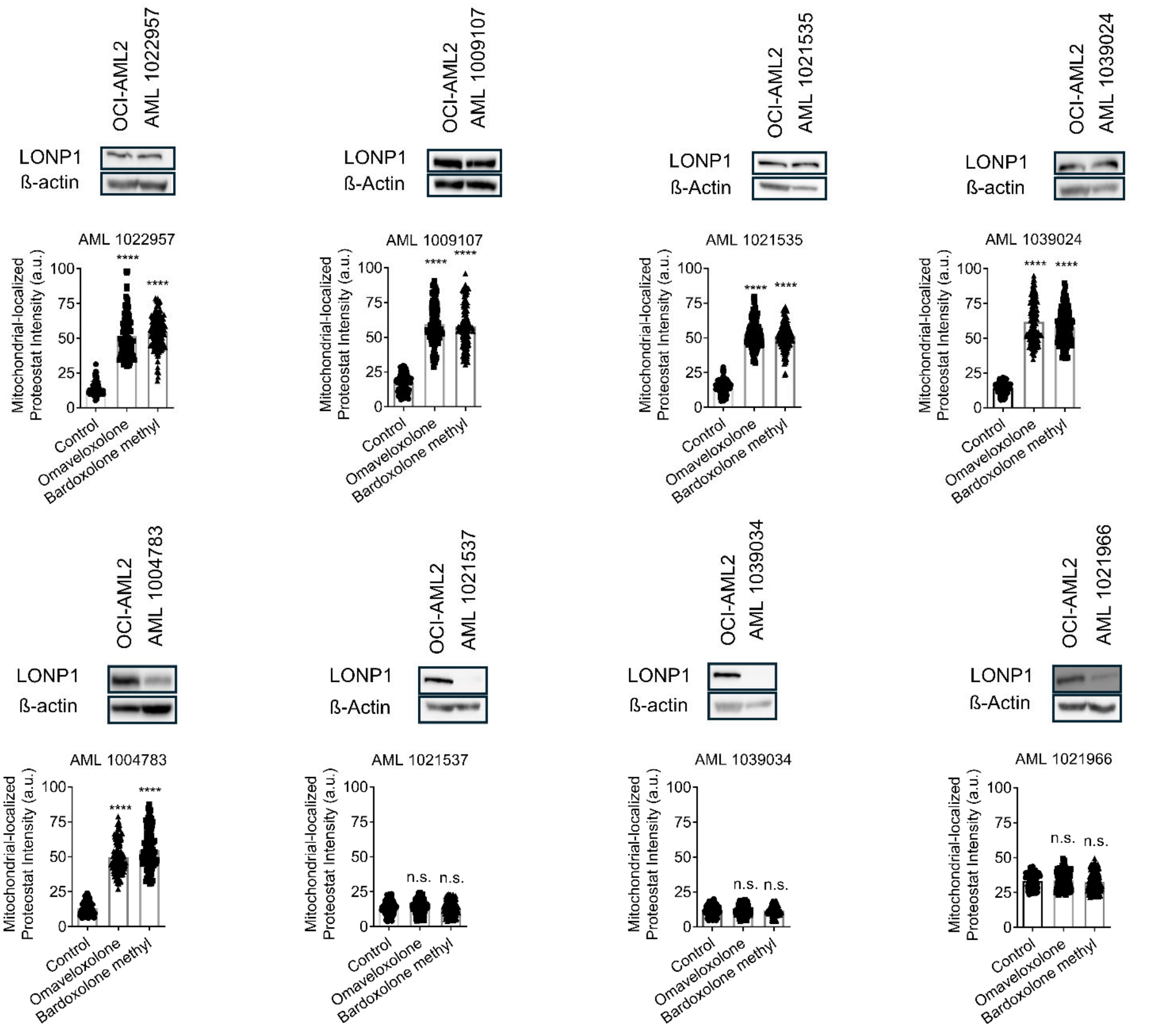

Figure S16

Figure S16. Mitochondrial protein aggregation is increased in primary AML cells treated with omaveloxolone or bardoxolone methyl.

Primary AML cells were treated with 250 nM omaveloxolone or bardoxolone methyl for 30 hours. After treatment, mitochondrial protein aggregation was measured as described in in Figure S12 and LONP1 expression relative to OCI-AML2 cells was measured by

immunoblotting. Colocalization of proteostat with TOMM20 was quantified by HALO Image analysis software (n=85-194). ****p<0.0001 (AML 1022957, AML 1009107, AML 1021535, AML 1039024, AML 1004783). n.s., p>0.05 (AML 1021537, AML 1039034, AML 1021966). Statistical analyses were conducted with a one-way ANOVA with Dunnett's multiple comparison test. See Supplementary table 2 (Patient cytogenetics).

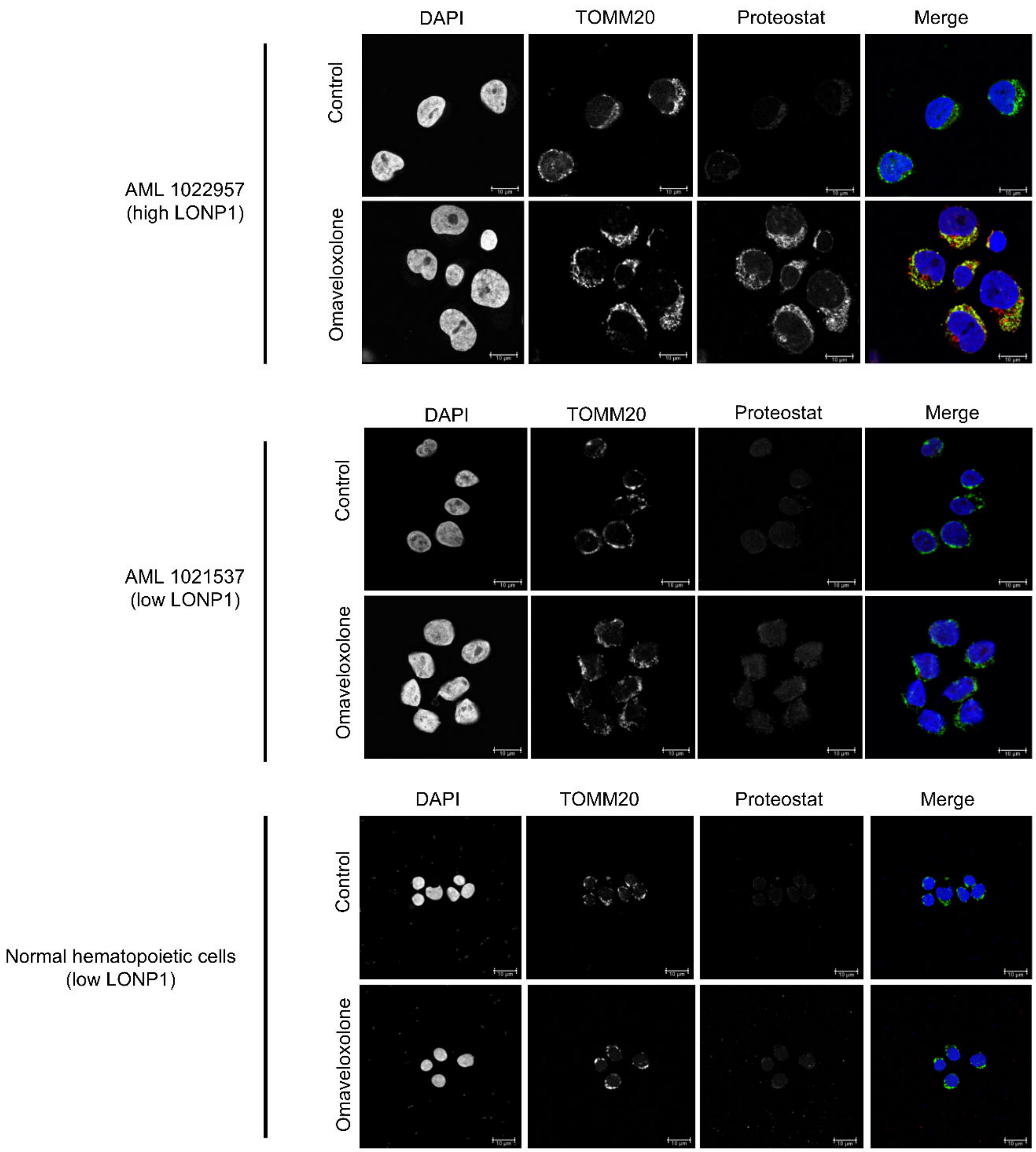

Figure S17

Figure S17. Mitochondrial protein aggregation in primary AML and normal hematopoietic cells treated with omaveloxolone.

Primary AML from Figure S16 and normal adult hematopoietic cells were treated with 250 nM omaveloxolone for 30 hours. After treatment, mitochondrial protein aggregation was

measured as described in Figure S12. Representative cells are shown. See Supplementary table 2 (Patient cytogenetics).

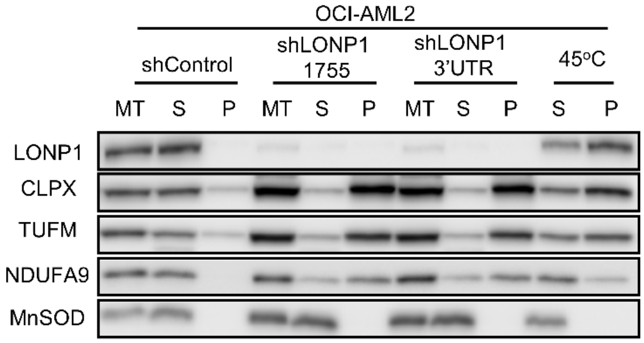

Figure S18

Figure S18. Knockdown of LONP1 in OCI-AML2 cells decreases mitochondrial protein solubility.

OCI-AML2 cells were transduced with shRNA targeting LONP1 or control sequences. 7 days after transduction, mitochondria were isolated. An aliquot of mitochondria from OCI-AML2 transduced with control sequences was heat shocked at 45°C for 30 minutes as a

positive control for aggregated protein. Levels of LONP1, CLPX, TUFM, NDUFA9, and MnSOD were measured in total (MT), soluble (S), and insoluble pellet (P) fractions of mitochondrial lysates. Representative immunoblot from n=3 replicates are shown.

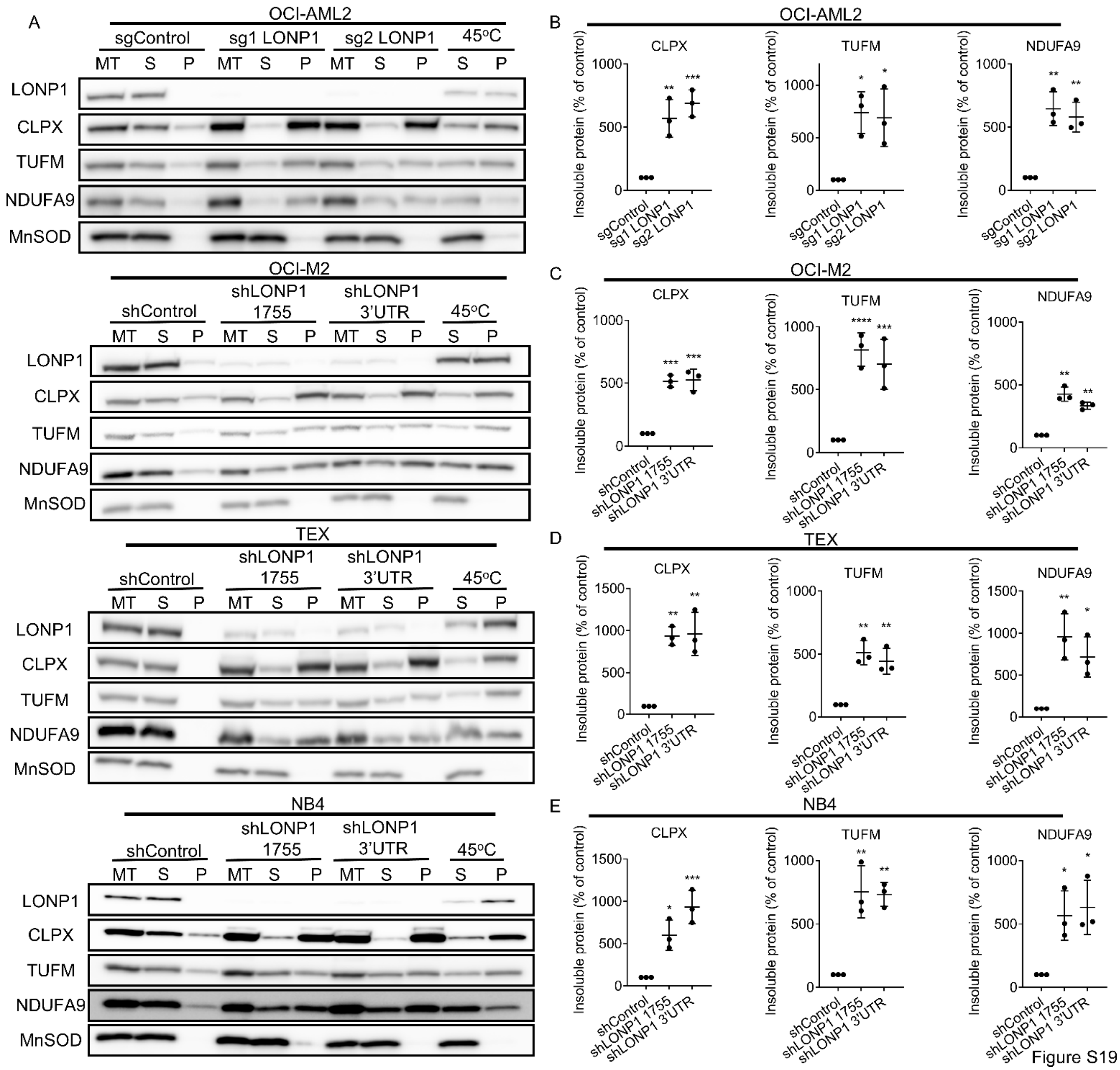

Figure S19. Genetic depletion of LONP1 in OCI-AML2, OCI-M2, TEX, and NB4 cells decreases mitochondrial protein solubility.

(A) OCI-AML2 expressing Cas9 or OCI-M2, TEX, and NB4 cells were transduced with gRNA or shRNA targeting LONP1 or control sequences, respectively. 7 (shRNA) or 14 (gRNA) days after transduction, mitochondria were isolated. Isolated mitochondria from

cells transduced with control sequences were heat shocked at 45°C for 30 minutes as a positive control for aggregated protein. Levels of LONP1, CLPX, TUFM, NDUFA9, and MnSOD were measured as described in Figure S18. Representative immunoblots are shown.

(B) Levels of CLPX, TUFM, NDUFA9 were measured in the detergent insoluble fraction of OCI-AML2 mitochondrial lysates from (A) as described in Figure S18. Expression was quantified by densitometry and data represent mean ± SD expression from 3 replicates. CLPX: sgControl vs sg1 LONP1 (**p=0.0029); sgControl vs sg2 LONP1 (***p=0.0009). TUFM: *$p<0.05$ (sgControl vs sg1 LONP1, sgControl vs sg2 LONP1). NDUFA9: **$p<0.01$ (sgControl vs sg1 LONP1, sgControl vs sg2 LONP1) by a one-way ANOVA with Dunnett's multiple comparison test.

(C) Levels of CLPX, TUFM, NDUFA9 were measured by immunoblotting in the detergent insoluble fraction of OCI-M2 mitochondrial lysates from (A) as described in Figure S18. Expression was quantified by densitometry and data represent mean ± SD expression from 3 replicates. CLPX: ***$p<0.001$ (shControl vs shLONP1 1755, shControl vs shLONP1 3'UTR). TUFM: shControl vs shLONP1 1755 (****$p<0.0001$); shControl vs shLONP1 3'UTR (***p=0.0004). NDUFA9: **$p<0.01$ (shControl vs shLONP1 1755, shControl vs shLONP1 3'UTR) by a one-way ANOVA with Dunnett's multiple comparison test.

(D) Levels of CLPX, TUFM, NDUFA9 were measured in the detergent insoluble fraction of TEX mitochondrial lysates by immunoblotting from (A) as described in Figure S18. Expression was quantified by densitometry and data represent mean ± SD expression from 3 replicates. CLPX: **$p<0.01$ (shControl vs shLONP1 1755, shControl vs shLONP1

3’UTR). TUFM: **p<0.01 (shControl vs shLONP1 1755, shControl vs shLONP1 3’UTR). NDUFA9: shControl vs shLONP1 1755 (**p=0.0045); shControl vs shLONP1 3’UTR (*p=0.0207) by a one-way ANOVA with Dunnett’s multiple comparison test.

(E) Levels of CLPX, TUFM, NDUFA9 were measured in the detergent insoluble fraction of NB4 mitochondrial lysates from (A) as described in Figure S18. Expression was quantified by densitometry and data represent mean ± SD expression from 3 replicates. CLPX: shControl vs shLONP1 1755 (*p=0.0127); shControl vs shLONP1 3’UTR (***p=0.0010). TUFM: **p<0.01 (shControl vs shLONP1 1755, shControl vs shLONP1 3’UTR). NDUFA9: *p<0.05 (shControl vs shLONP1 1755, shControl vs shLONP1 3’UTR) by a one-way ANOVA with Dunnett’s multiple comparison test.

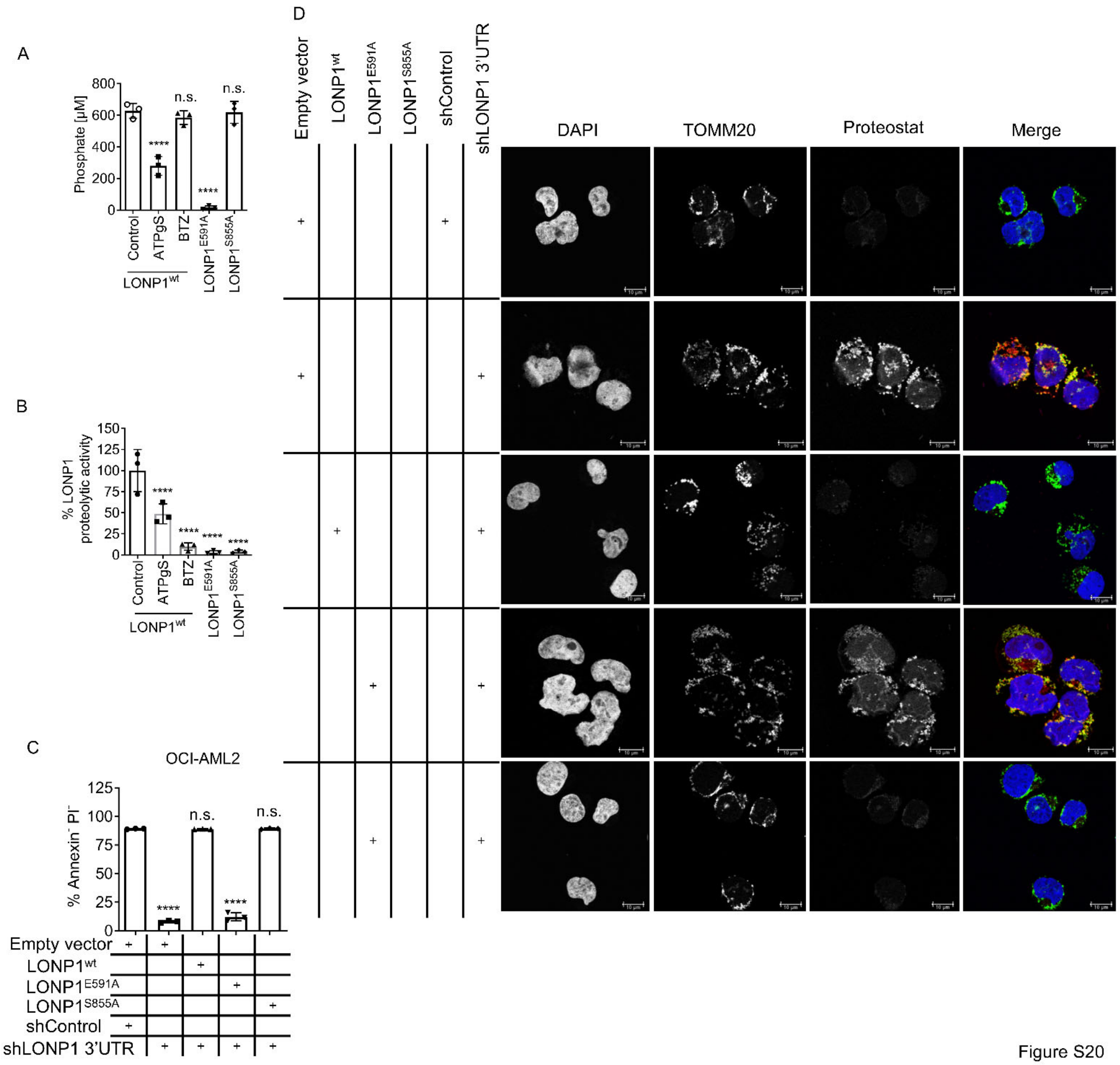

968

969 **Figure S20. LONP1 ATPase activity is essential for mitochondrial protein solubility**
970 **and viability in OCI-AML2 cells.**

971 (A) Recombinant wild type (LONP1^{wt}), ATPase-deficient (LONP1^{E591A}), or proteolytically-
972 deficient LONP1 (LONP1^{S855A}) were treated with 2 mM of the AAA+ domain inhibitor
973 ATPgS or 50 µM of the protease domain inhibitor bortezomib (BTZ), or vehicle control.

ATPase activity was measured as in Figure 3A. Data represent mean ± SD ATPase activity. ****$p<0.0001$ (LONP1^{wt} control vs LONP1^{wt} ATPgS, LONP1^{wt} control vs LONP1^{E591A}); n.s., $p>0.05$ (LONP1^{wt} control vs LONP1^{wt} BTZ, LONP1^{wt} control vs LONP1^{S855A}). Statistical analyses were conducted with a one-way ANOVA with Dunnett's multiple comparison test.

(B) Recombinant wild type (LONP1^{wt}), ATPase-deficient (LONP1^{E591A}), or proteolytically-deficient LONP1 (LONP1^{S855A}) LONP1 were treated with 2 mM of the AAA+ domain inhibitor ATPgS or 50 µM of the protease domain inhibitor bortezomib (BTZ), or vehicle control. Proteolytic activity was measured as in Figure 3B. Data represent mean ± SD proteolytic activity. ****$p<0.0001$. Statistical analyses were conducted with a one-way ANOVA with Dunnett's multiple comparison test.

(C) OCI-AML2 cells were transduced with FLAG-tagged wild type (LONP1^{wt}), ATPase-deficient (LONP1^{E591A}), proteolytically-deficient LONP1 (LONP1^{S855A}) cDNA or empty vector. 14 days later, cells were transduced with shRNA targeting the 3'UTR of endogenous LONP1 or control sequences. 7 days after transduction, mean ± SD viability was assessed by annexin V/propidium iodide staining and flow cytometry. ****$p<0.0001$ (Empty vector+shControl vs Empty vector+shLONP1 3'UTR, Empty vector+shControl vs LONP1^{E591A} LONP1+shLONP1 3'UTR); n.s., $p>0.05$ (Empty vector+shControl vs LONP1^{wt}+shLONP1 3'UTR, Empty vector+shControl vs LONP1^{S855A}+shLONP1 3'UTR). Statistical analyses were conducted with a one-way ANOVA with Dunnett's multiple comparison test.

(D) OCI-AML2 cells from (C) were stained as described in Figure S12 to detect mitochondrial protein aggregation. Representative cells are shown.

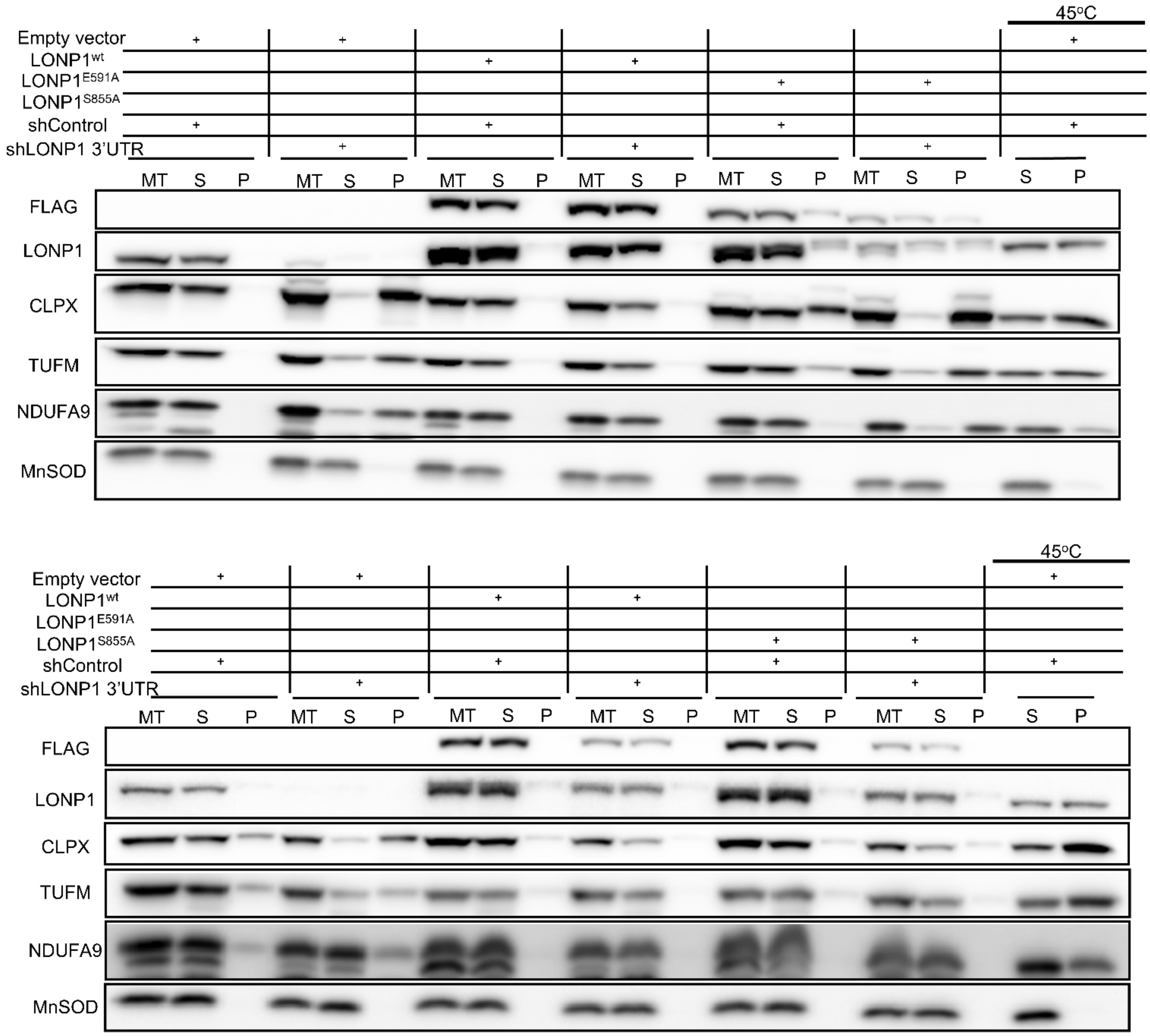

Figure S21

Figure S21. LONP1 ATPase activity is required for mitochondrial protein solubility. OCI-AML2 cells were transduced with FLAG-tagged wild type (LONP1^{wt}), ATPase-deficient (LONP1^{E591A}), proteolytically-deficient (LONP1^{S855A}) LONP1 cDNA or empty vector. 14 days later, cells were transduced with shRNA targeting the 3'UTR of endogenous LONP1 or control sequences. 7 days after transduction, levels of LONP1,

CLPX, TUFM, NDUFA9, and MnSOD were measured in total (MT), soluble (S), and insoluble pellet (P) fractions of mitochondrial lysates. Representative immunoblots from n=3 replicates are shown.

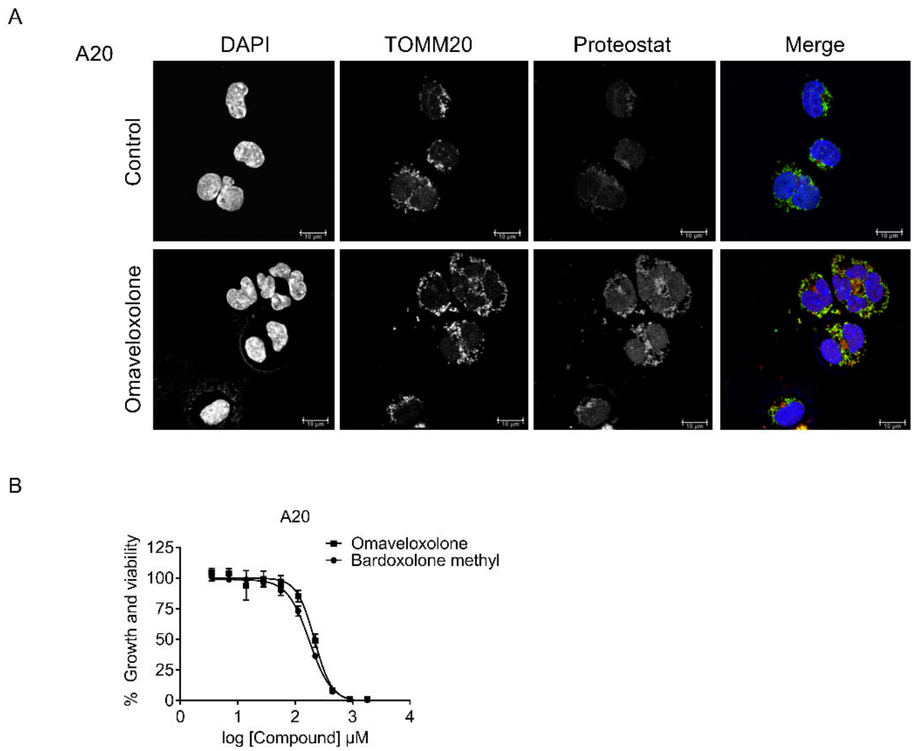

Figure S22

Figure S22. Omaveloxolone increases mitochondrial protein aggregation and kills A20 murine leukemia/lymphoma cells.

(A) A20 cells were treated with 250 nM omaveloxolone or vehicle control. After 30 hours of incubation, cells were stained as described in Figure S12 to detect mitochondrial protein aggregation and imaged by confocal microscopy. Representative cells are shown.

(B) A20 cells were treated with increasing concentrations of omaveloxolone and bardoxolone methyl for 72 hours. Mean ± SD growth and viability was measured with CellTiter-Fluor.

Supplementary Tables 1- 3

Supplemental Table 1. UPR^{mt} signature literature sources

Gene	Literature (PMID)
HSP8A	12526792, 29930205, 38384479, 30429537
HSPA1A, HSPA1B, HSPA1L	37286597, 28484090, 12526792, 16172114, 14752510, 33602268
HSPA9	33431889, 34400774, 35853457, 21811887, 24743735
HSP90AA1, HSP90AB1	12526792, 29394093, 27402847, 22178133, 17957250, 17956728, 27402847, 32236591
TRAP1	25088416, 27350246, 31987035, 17853063
HSPD1	2528694, 32060690, 37286597, 1359644, 38588611, 35653190, 12198143
HSPE1	17849004, 37286597, 31269451, 1348860, 32060690
DNAJA1	37286597, 9192730, 9382858
HSF1	39122169, 32302062, 25762445, 37286597
TOMM34	22178133, 36763002, 32371396, 11913975
NLN	32269163, 29183787, 38906862
CLPX, CLPP	12198143, 26058080, 31056398, 33637676, 26142927, 35653190
YME1L1	17849004, 25433032, 26923599, 30544562, 24176854
CEBPB	17848986, 17849004, 21364280, 34039602, 12198143
CLPB	35499078, 38270563, 32573439, 31048321, 34115842
HTRA2	19023330, 16968707, 37286597, 23542127
AFG3L2	26504172, 30544562, 35912435, 23041622
SPG7	14623864, 9635427, 31097542, 32973427
LONP1	33431889, 34461102, 33637676, 37286597, 27350246, 25574826, 37217599, 35895846, 34400774
ATF4	37738349, 28566324, 32132707, 36113464
ATF5	27426517, 37738349, 35895846
CHOP	17849004, 37738349, 17848986, 23761072, 12198143, 32132707
PMPCA and PMPCB	17849004, 35388015, 25176146, 32491259
TXN2	17849004, 26687188, 12032145, 12080052
ENDOG	17849004, 11452314, 24129513, 11753562
ATAD1	24843043, 36409067, 39024102
OMA1	32132707, 32132706, 36113464, 38530280, 24550258
USP30	32142685, 34653676, 32142684
MARCH5	20851218, 19741096, 22308378, 32142684
PITRM1	32632204, 25176146, 35388015, 26697887
UBQLN1	27345149, 28933694, 38902824
PARL	12774122, 33556373, 28178523, 29301859
VCP	21118995, 25062828,
DNAJA3	12198143, 33431889, 21811887

Supplementary Table 1. UPR^{mt} signature literature sources

A literature search identified 39 genes contributing to mitochondrial proteostasis.

Supplementary Table 2. Patient Demographics

Sample ID	Diagnosis (Dx)	Source	NPM 1	FLT3-ITD	FLT3-TKD	Cytogenetics	Age at Dx	Sex
1022957	Acute myeloid leukemia	PB	Undetectable	Undetectable	Undetectable	Complex Abnormal	57	Male
90558	AML	PB				46,XX,inv(3)(q21q26.2)[19]/46,XX[1]	51	Female
1038828	AML with mutated NPM1	PB	Positive	Undetectable	Undetectable	46,XY[20]	80	Male
161153	AML with myelodysplasia-related changes	PB	Undetectable	Positive		46,XX[20]	68	Female
151656	AML with NPM1 mutation	PB	Positive	Undetectable	Undetectable	46,XX[20]	78	Female
827875	AML with myelodysplasia-related changes	PB	Undetectable	Undetectable	Undetectable	45~46,XY,-3,del(5)(q31q35),-7,-9,+13,+22,+mar1,+mar2[cp29]	59	Male
100356	AML, M4 Myelomonocytic	PB				45,XY,-5,der(12)t(5;12)(q13;p12~13),+del(16)(q22),-17, i(17)(q10)[8]/46,XY,idem,+21[2]	55	Male
130853	AML, M5b Monocytic with maturation	PB				46,XX[20]	73	Female
100388	AML, M5a Monocytic without maturation	PB				48,XX.+8,+r[6]/48,XX,+1,der(1;22)(q10;q10),+8, +r[7]/48,XX,+1,der(1;21)(q10;q10),+8,+r[6]/48,XX,+1,add(1)(p11),+8[2]	72	Female
192030	AML	PB	Positive	Undetectable	Undetectable	46,XX[17]	74	Female
1039024	AML with mutated NPM1	PB	Positive	Undetectable	Undetectable	inconclusive	73	Female
160853	AML with myelodysplasia-related changes	PB				43~44,XX,add(2)(q33)[2],del(3)(p21)[2],-5[8],del(5)(q13q33)[2],add(6)(q21)[2],add(7)(q32)[6],del(12)(p11.2p13)[2],del(12)(q13q24.1)[6],-17[3],dic(17;21)(p13;p11.2)[5],-18[10], +1~3mar[8],dmin[2] [cp10]	76	Female
844355	AML with mutated NPM1	PB	Positive	Undetectable	Inconclusive	46,XX[20]	82	Female
245174	AML	PB	Positive	Undetectable		46,XY[20]	76	Male
140301	AML, M5b Monocytic with maturation	PB	Positive	Undetectable	Undetectable	46,XX[20]	60	Female
161868	AML	PB	Positive	Positive		46,XY[20]	63	Male
185484	AML	PB	Positive	Positive		unsuccessful	58	Female
884575	AML with myelodysplasia-related changes	PB	Undetectable	Undetectable	Undetectable	45~50,XY,del(5)(q14-21q32-34),add(7)(q11.2),add(7)(q21),+8,-11,-13,-17,-18,-21,-21,+22,+1~6mar[cp14]	65	Male
80250	AML, M2 Myeloblastic with maturation	PB				46,XX,del(7)(q22)[4]/46,XX[18]	71	Female
120805	AML	PB	Positive	Undetectable	Undetectable	46,XY[20]	29	Male

1039134	AML, NOS	PB	Undetectable	Undetectable	Undetectable	46,XX[24]	46	Female
186253	AML	PB	Undetectable	Undetectable		46,XY,-7,+r[3]/46,XY,add(4)(q12),-7,der(17)t(4;17)(q12;q23),+r[16]/46,XY[1]	82	Male
161476	AML	PB	Positive	Positive		unsuccessful	55	Female
207202	AML	PB	Positive	Undetectable		46,XX[23]	67	Female
161965	AML with myelodysplasia-related changes	BM	Undetectable	Undetectable		46,XX[20]	76	Female
100857	AML, M2 Myeloblastic with maturation	PB				46,XY,+8[9]	73	Male
110633	AML, M1 Myeloblastic without maturation	PB				47,XX,+10[7]/46,XX[13]	61	Female
1021966	Acute promyelocytic leukemia with PML-RARA	BM	Undetectable	Undetectable	Undetectable	ogm[GRCh38] inv/t(9;9)(p24.3;p21.2)(1,055,653_26,643,447)[0.96]?c,t(15;17)(q24.1;q21.2)(74,029,809;40,348,183)(PML::RARA)[0.52]	34	Male
1036067	Acute myeloid leukemia with MECOM rearrangement.	PB	Undetectable	Undetectable	Undetectable	46,XX,t(2;3)(p21;q26),del(5)(q22q35)[12]/46,XX[2]	54	Female
160556	AML with myelodysplasia-related changes	PB				46,XY,t(3;5)(q21;q35)[10]	54	Male
1021535	AML with myelodysplasia-related changes	PB	Undetectable	Undetectable	Undetectable		47	Male
1038679	AML with myelodysplasia-related changes	PB	Undetectable	Undetectable	Undetectable		47	Male
90381	AML with myelodysplasia-related changes	PB	Undetectable	Positive	Undetectable	47,XY,+X[20]	62	Male
6164	AML, M5a Monocytic without maturation	PB				47,XY,+8[17]/46,XY[3]	76	Male
90784	AML, M5a Monocytic without maturation	PB	Positive	Positive	Undetectable	46,XX[20]	61	Female
199810	AML	PB	Positive	Positive		46,XY[20]	84	Male
1004783	Acute myeloid leukemia, therapy related	PB	Undetectable	Undetectable	Undetectable	46,XX,t(9;11)(p21;q23)[18]/46,XX[3]	68	Female

162111	AML	PB	Positive	Undetectable		45,X,-Y[9]/46,XY[11]Result by interphase FISH: negative for KMT2A rearrangement, negative for monosomy 5/5q deletion; negative for monosomy 7/7q deletion.	18	Male
1009107	Acute myeloid leukemia with RUNX1:: RUNX1T1	PB	Undetectable	Undetectable	Undetectable	46,XY,t(8;21)(q22;q22)[20]	46	Male
1021537	Acute myeloid leukemia with MECOM rearrangement	PB	Undetectable	Undetectable	Undetectable	3,7,12 ins(3;12;7)(q26.2;p13.2p13.2;q31.1;p13.2)	34	Female
220776	AML with NPM1 mutation	PB	Positive	Undetectable		46,XY[20]	83	Male
203471	AML with inv(16)(p13.1q22) or t(16;16)(p13.1;q22); CBFB-MYH11	PB	Undetectable	Positive		ish t(16;16) (p13.1;q22) (3'CBFB+;3'CBFC-) [3]	68	Male
172036	AML	PB	Positive	Positive		46,XY[20]	83	Male
120697	AML	PB	Undetectable	Undetectable	Undetectable	46,XY[20]	52	Male
1007932	Acute myeloid leukemia with myelodysplasia-related changes	PB	Undetectable	Undetectable	Undetectable	45~46,XY,add(3)(q11.2),-4,-5,-7,add(10)(q22),-11,-16,-17,add(19)(p13.1),+5~6mar[10]/46,XY[4]	69	Male
130433	AML, M4 Myelomonocytic	PB	Undetectable	Undetectable	Undetectable	46,XY[20]	77	Male
80551	AML, M1 Myeloblastic without maturation	PB				46,XY,?inv(3)(q21q25)[3]/46,XY[40]	32	Male
90147	AML	PB				45,XX,-7[20]	60	Female
223566	AML with myelodysplasia-related changes	PB				46,XY[20]	75	Male
130579	AML, NOS	BM	Undetectable	Undetectable	Undetectable	46,XY[20]	62	Male
183671	AML with mutated NPM1	PB	Positive	Positive		46,XY[20]	40	Male

Supplementary Table 2. Primary AML IDs and cytogenetics

A compilation of primary AML IDs used in the study and their corresponding patient diagnosis, source of the cells used, presence of NPM1 and FLT3 mutations, cytogenetic profile, age of diagnosis and sex.

Cell line	Species	Tissue	Sex	Source
**OCI AML2**	*Homo sapiens*	Peripheral Blood	Male	Dr. Mark D. Minden
**OCI M2**	*Homo sapiens*	Peripheral Blood		Dr. Mark D. Minden
**NB4**	*Homo sapiens*	Bone marrow	Female	DSMZ
**TEX**	*Homo sapiens*	Cord Blood	N/A	Dr. John Dick
**A20**	*Mus musculus*	Peripheral blood	N/A	ATCC

Supplemental Table 3: Cell lines used in the study
