# Supplementary material for: Elevated mitochondrial protein import in acute myeloid leukemia increases reliance on mitochondrial protease LONP1
Source: J Clin Invest. 2026 Jun 16;136(15):e196687. doi: 10.1172/JCI196687 (PMC13430017; doi:10.1172/JCI196687)

Elevated mitochondrial protein import in acute myeloid leukemia increases reliance on mitochondrial protease LONP1

Uncropped images of western blots used in the manuscript

Figure 2A, S9A

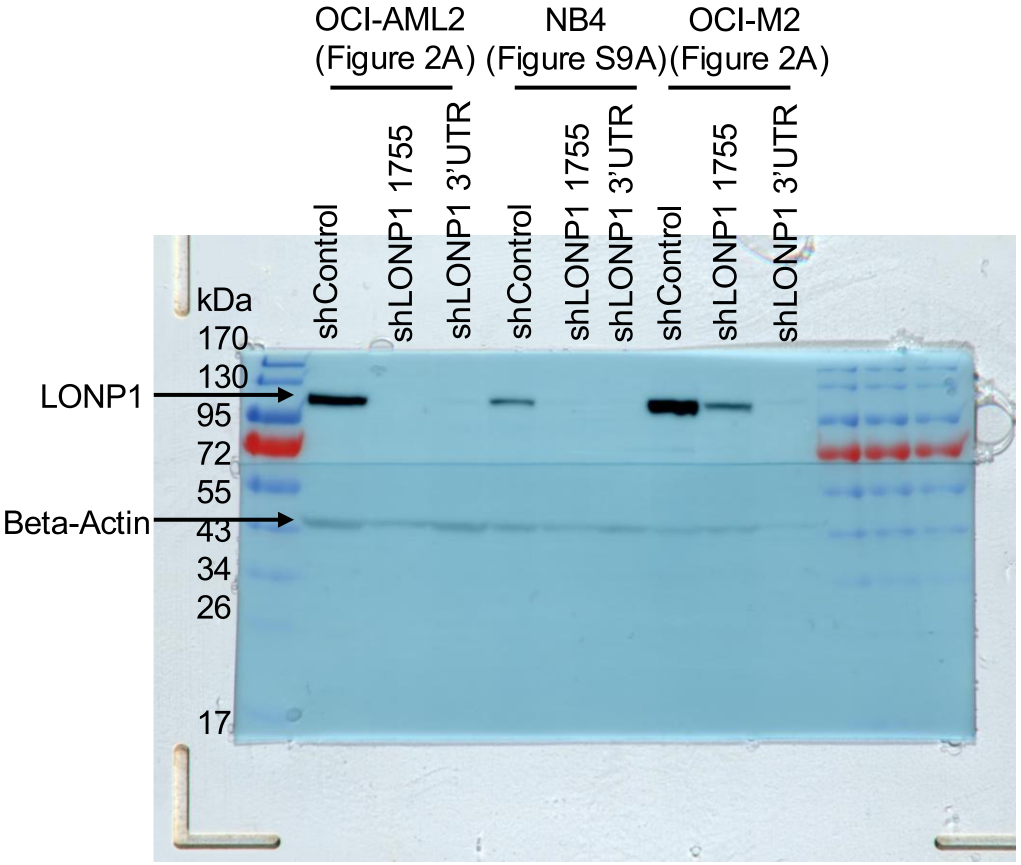

Figure 2A

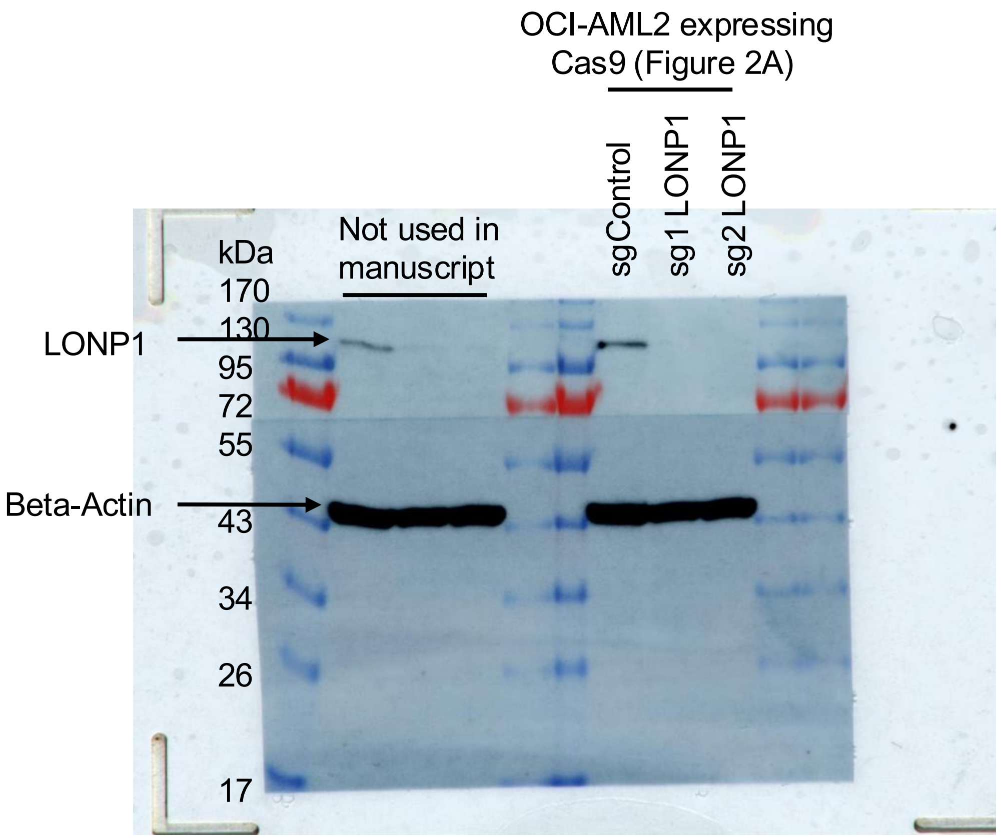

OCI-AML2 expressing
Cas9 (Figure 2A)
sgControl
sg1 LONP1
sg2 LONP1
Not used in
manuscript
kDa
170
130
95
72
55
43
34
26
17
LONP1
Beta-Actin

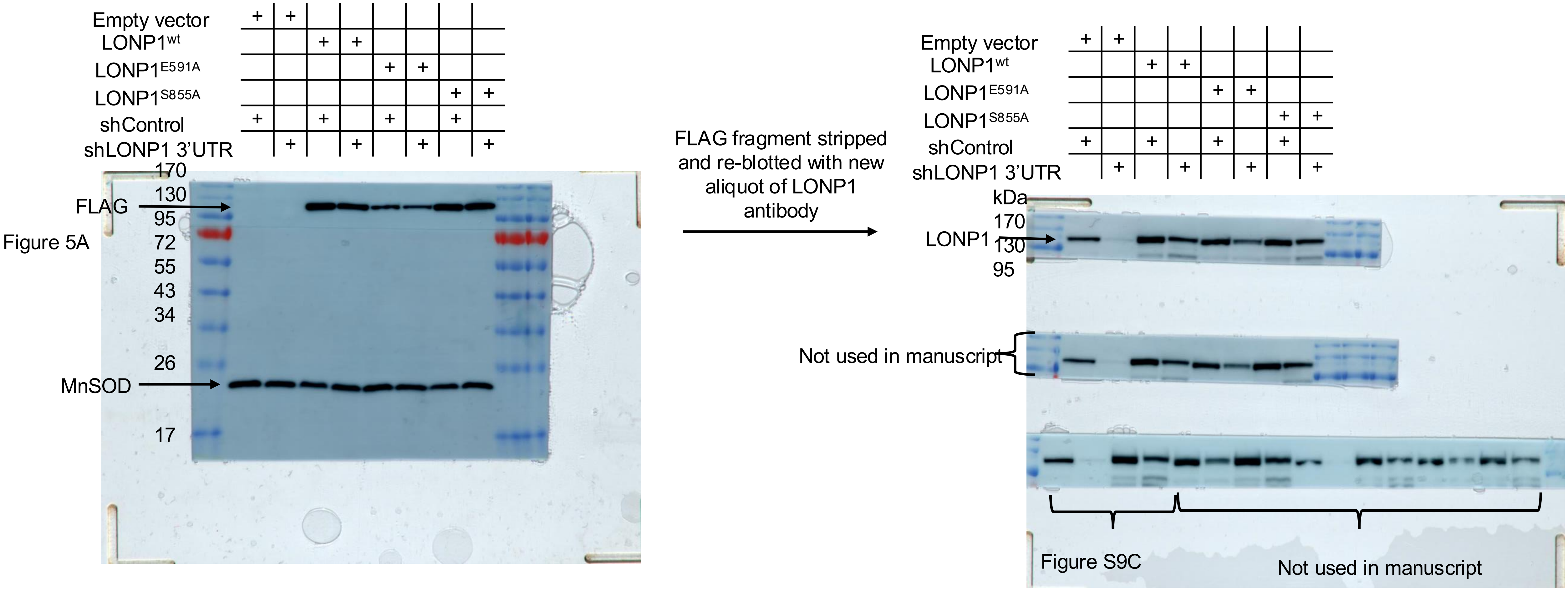
Empty vector
LONP1wt
LONP1E591A
LONP1S855A
shControl
shLONP1 3'UTR
170
130
95
72
55
43
34
26
17
FLAG
Figure 5A
MnSOD
FLAG fragment stripped and re-blotted with new aliquot of LONP1 antibody
Empty vector
LONP1wt
LONP1E591A
LONP1S855A
shControl
shLONP1 3'UTR
kDa
170
130
95
LONP1
Not used in manuscript
Figure S9C
Not used in manuscript

Figure 6A

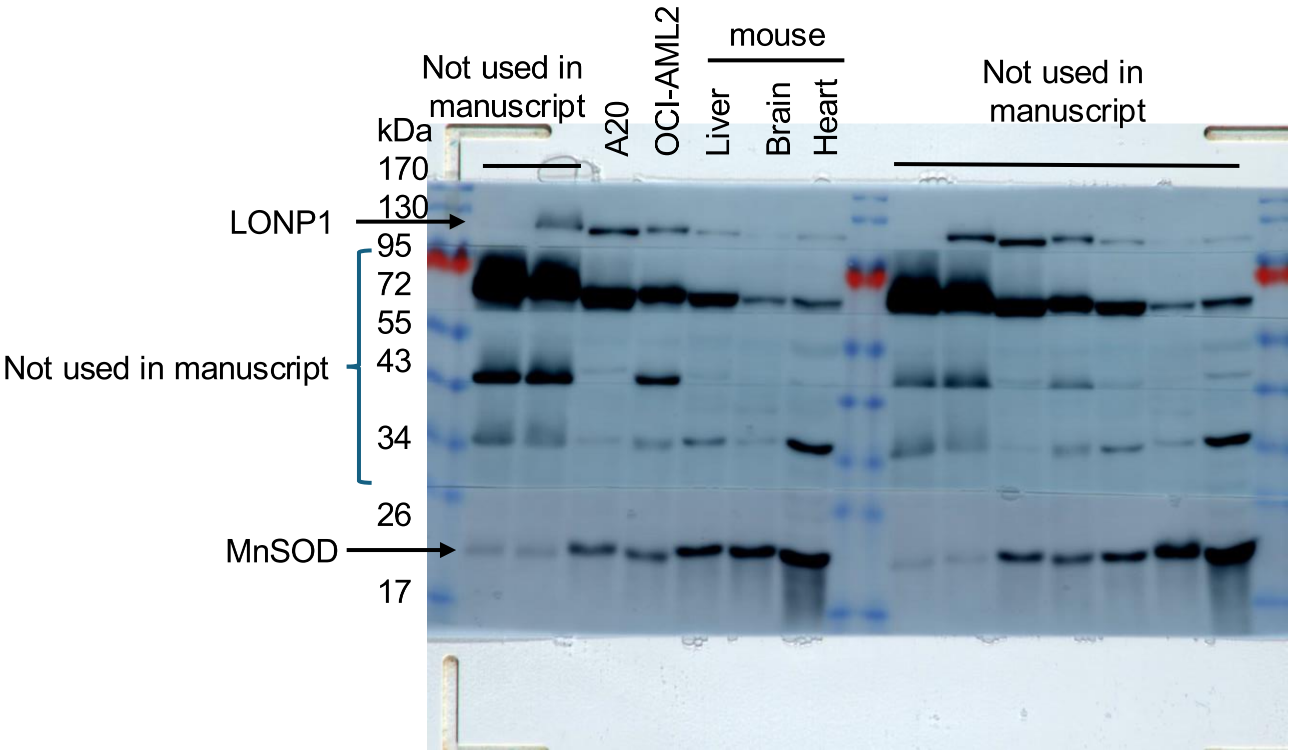
Not used in manuscript
A20
OCI-AML2
mouse
Liver
Brain
Heart
Not used in manuscript
kDa
170
130
95
72
55
43
34
26
17
LONP1
Not used in manuscript
MnSOD

Figure 6B

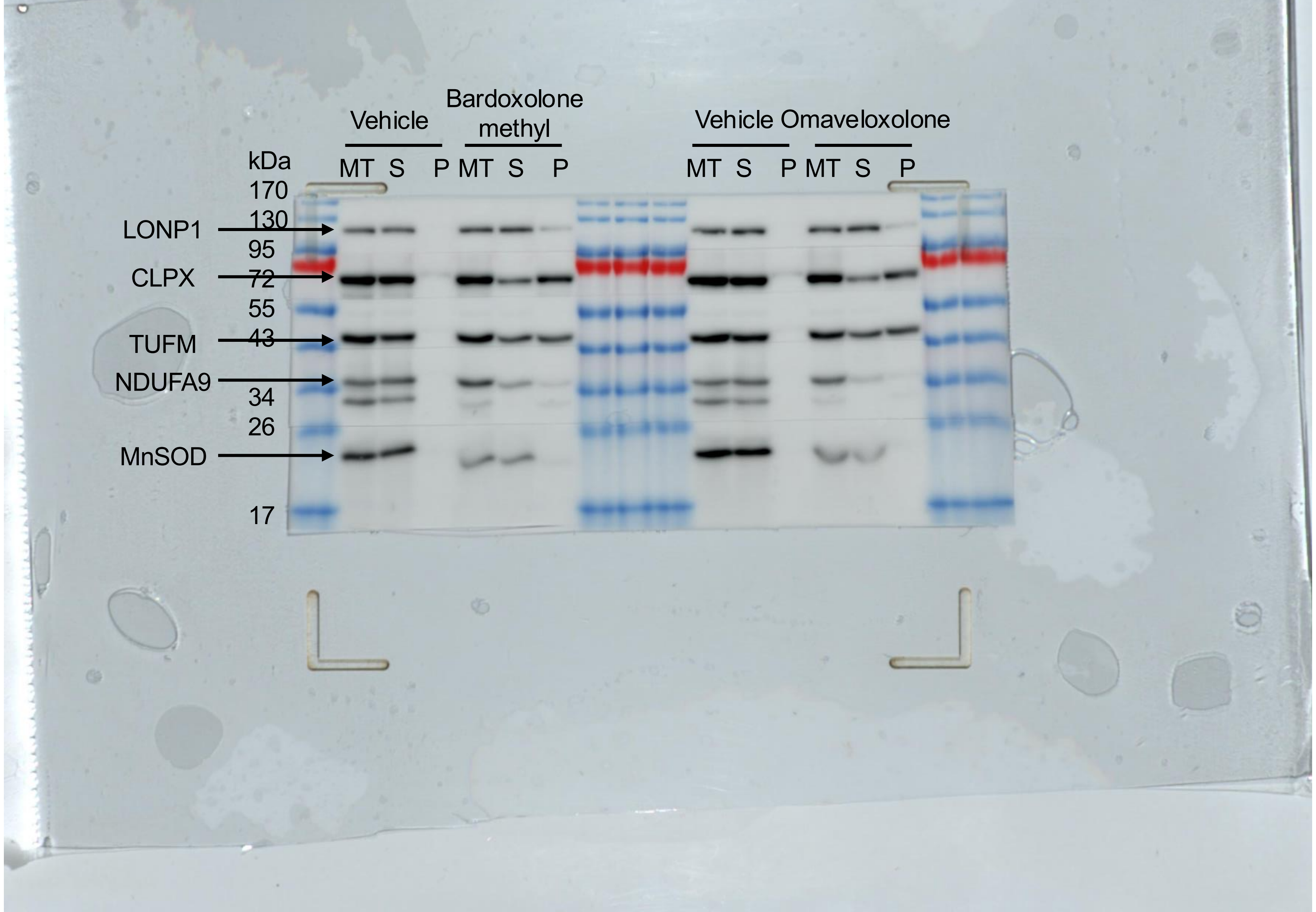
Vehicle
Bardoxolone methyl
Vehicle
Omaveloxolone
kDa
MT S P MT S P
MT S P MT S P
170
130
95
72
55
43
34
26
17
LONP1
CLPX
TUFM
NDUFA9
MnSOD

Figure 6C

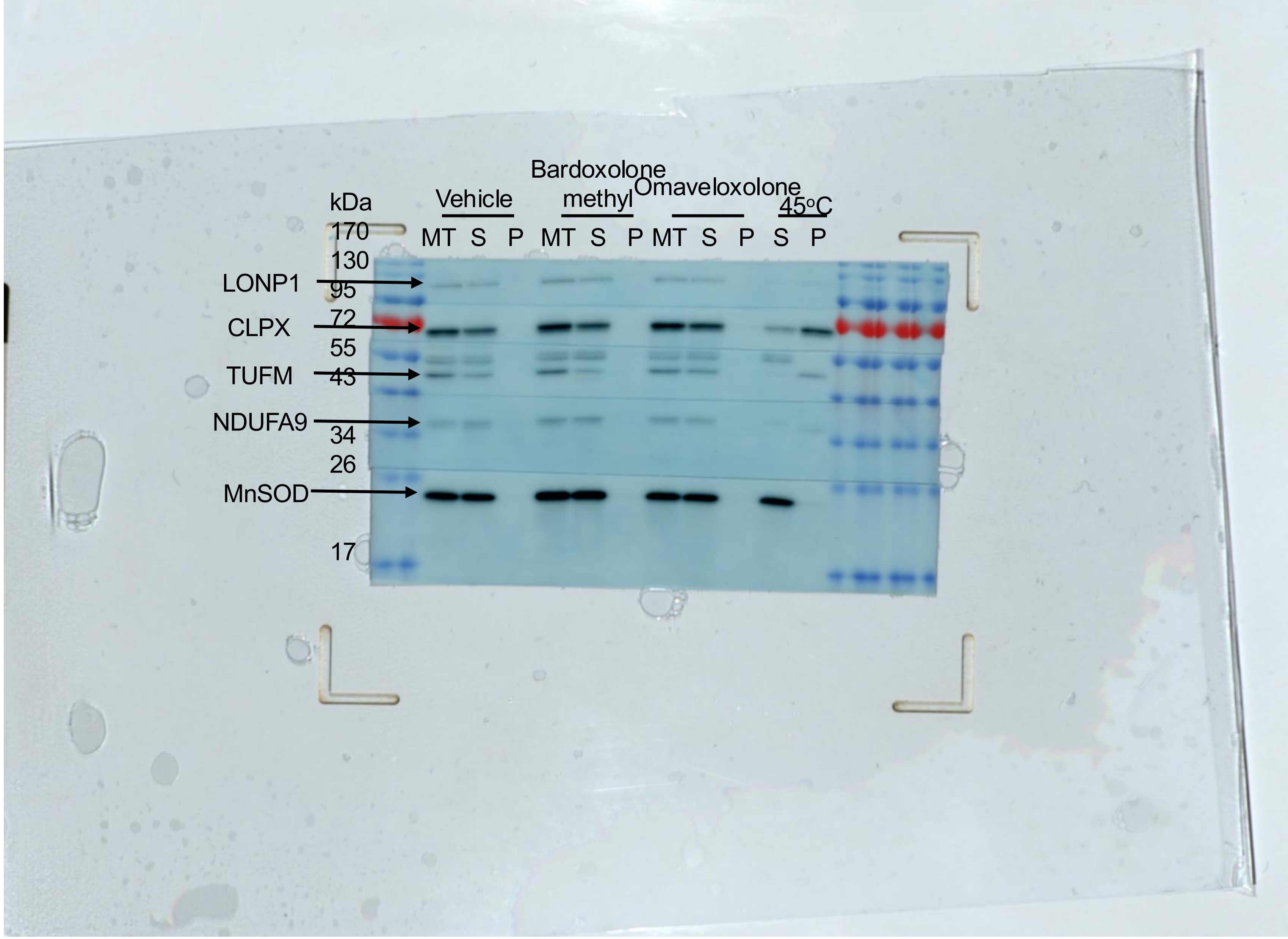

Bardoxolone methyl
Vehicle
Omaveloxolone
45oC
kDa
170
130
95
72
55
43
34
26
17
MT S P MT S P MT S P S P
LONP1
CLPX
TUFM
NDUFA9
MnSOD

Figure 6D

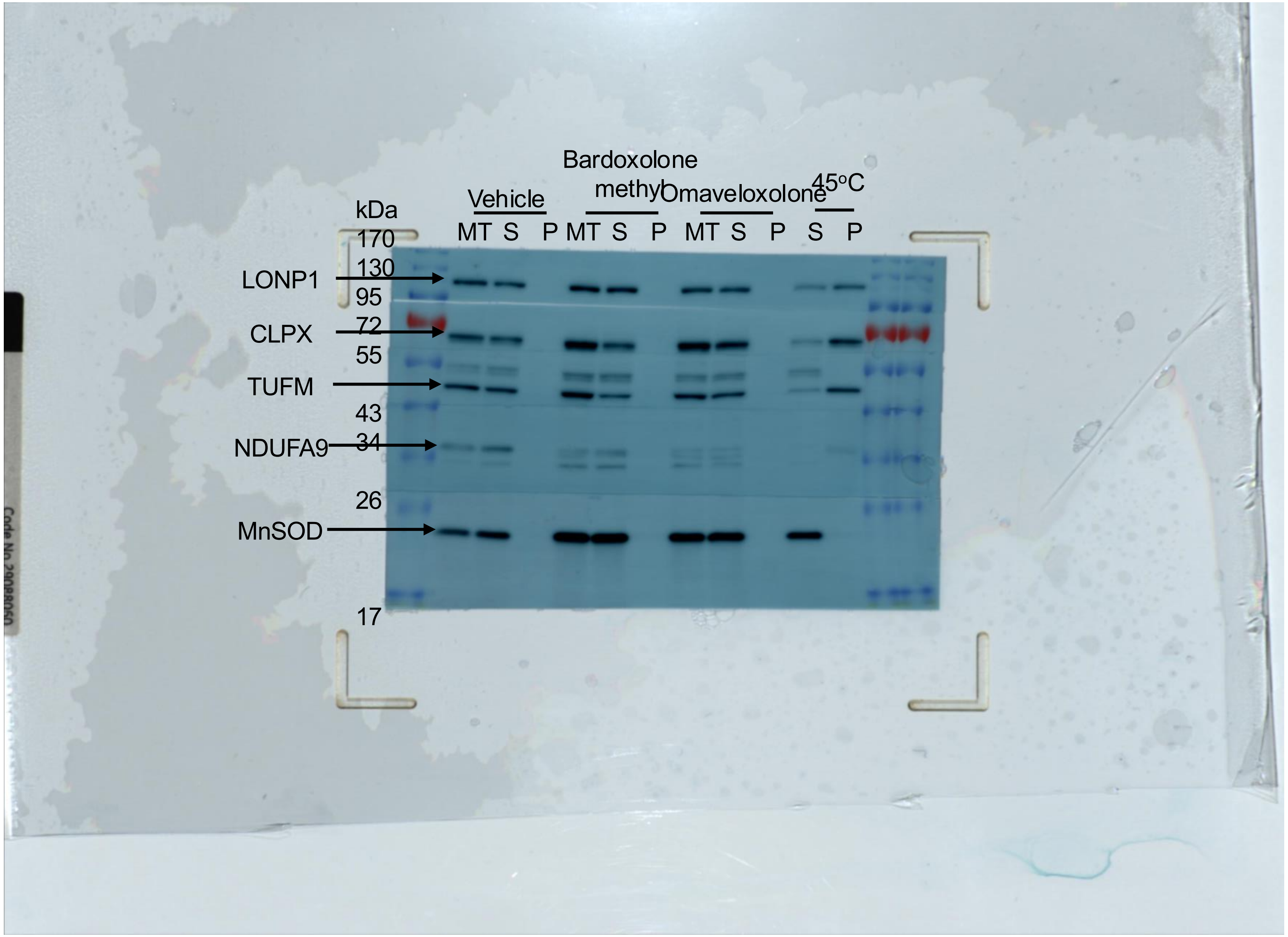

Bardoxolone methyl
Vehicle
Omaveloxolone
45°C
kDa
MT S P MT S P MT S P S P
170
130
95
72
55
43
34
26
17
LONP1
CLPX
TUFM
NDUFA9
MnSOD

Figure 6E

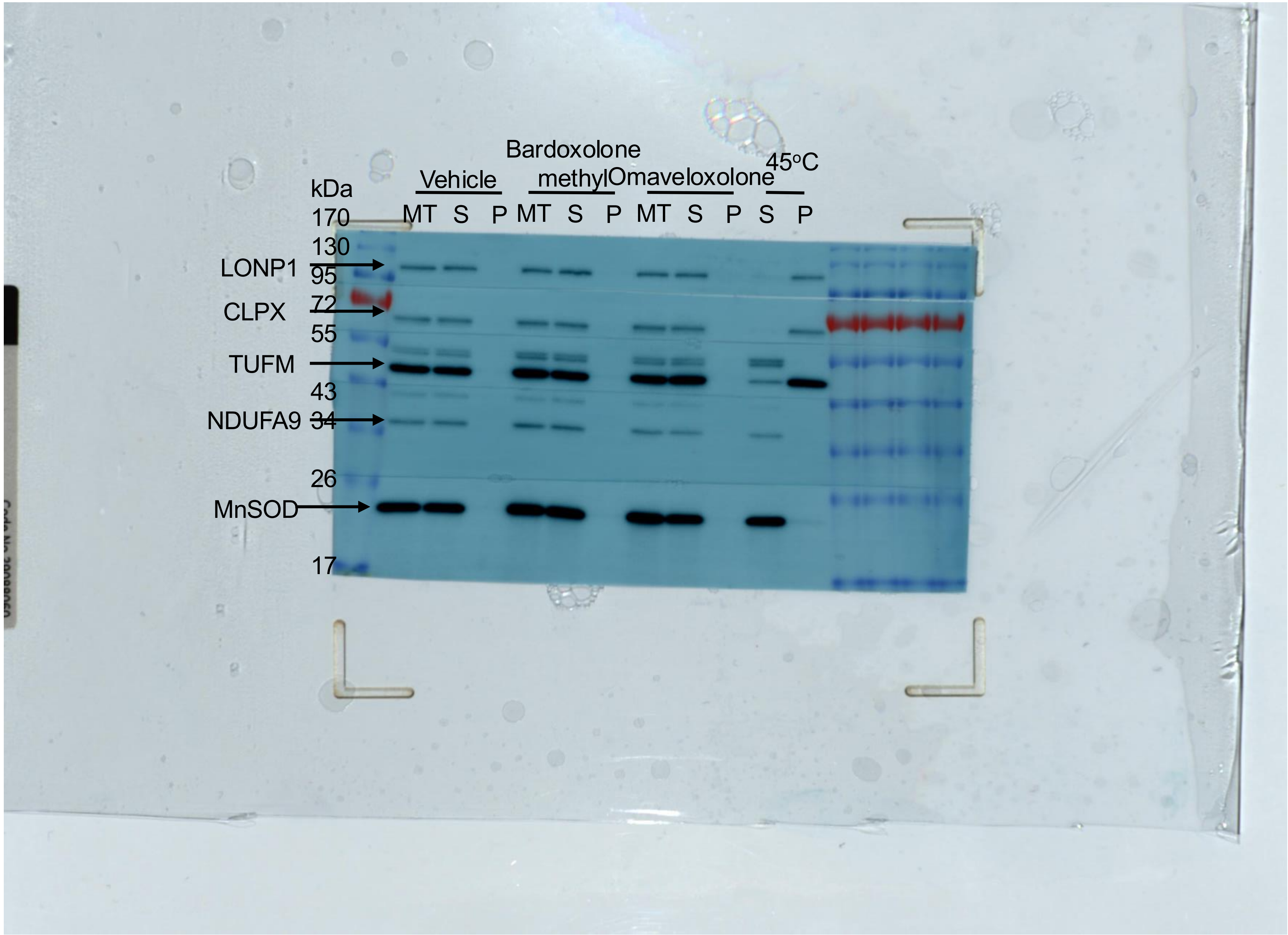

kDa
170
130
95
72
55
43
34
26
17
Vehicle
Bardoxolone methyl
Omaveloxolone
45°C
MT S P MT S P MT S P S P
LONP1
CLPX
TUFM
NDUFA9
MnSOD

Figure S5B

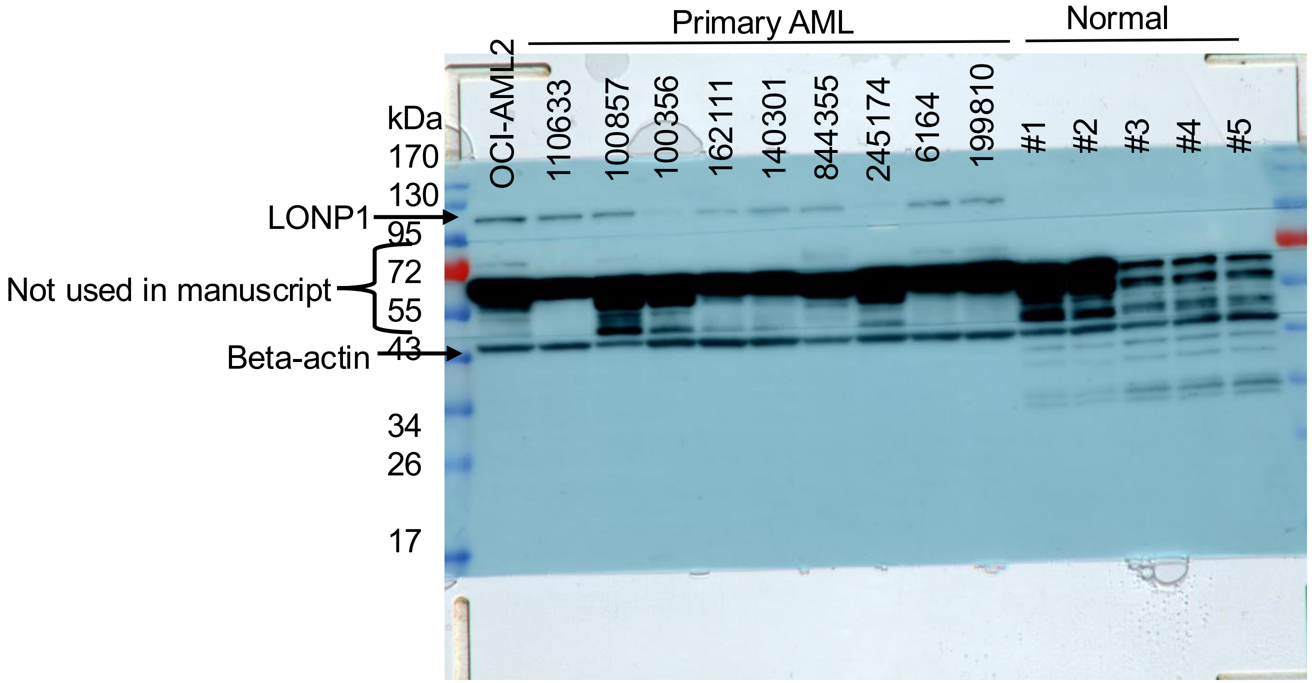

Primary AML
Normal
OCI-AML2
110633
100857
100356
162111
140301
844355
245174
6164
199810
#1
#2
#3
#4
#5
kDa
170
130
95
72
55
43
34
26
17
LONP1
Not used in manuscript
Beta-actin

Figure S5B

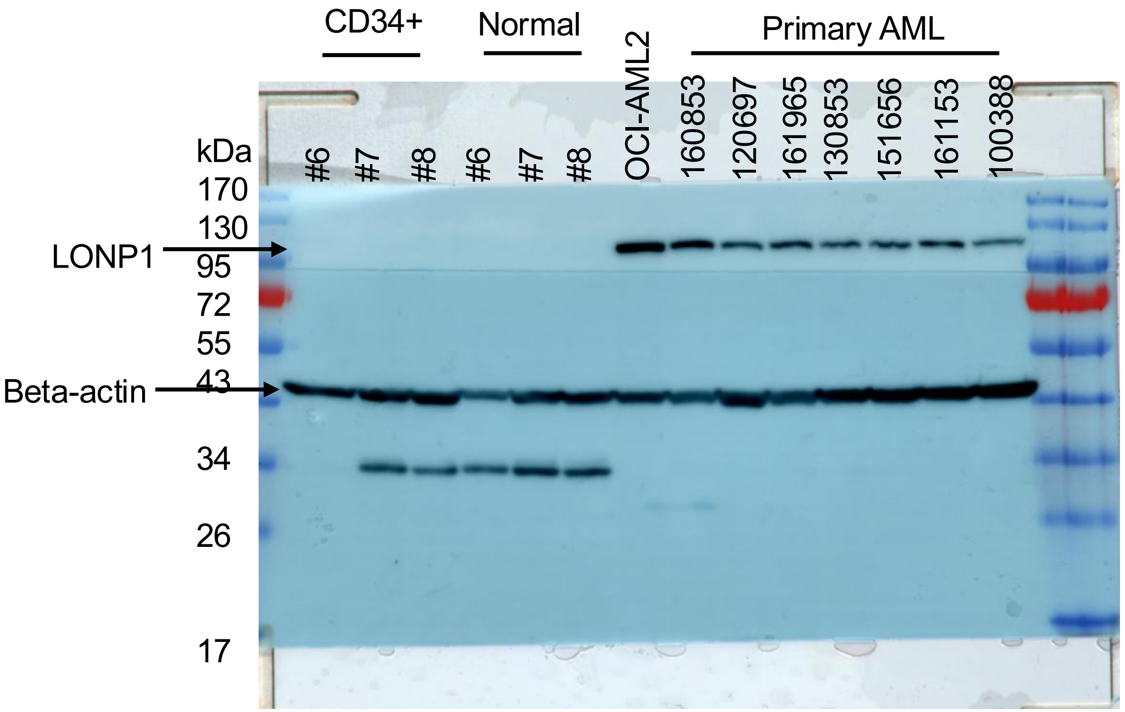
CD34+
Normal
Primary AML
OCI-AML2
160853
120697
161965
130853
151656
161153
100388
kDa
#6
#7
#8
#6
#7
#8
170
130
LONP1
95
72
55
Beta-actin
43
34
26
17

Figure S5B

Unused fragment on original blot with extremely high signal was removed and blot without this fragment was re-imaged to the left.

Not used in manuscript

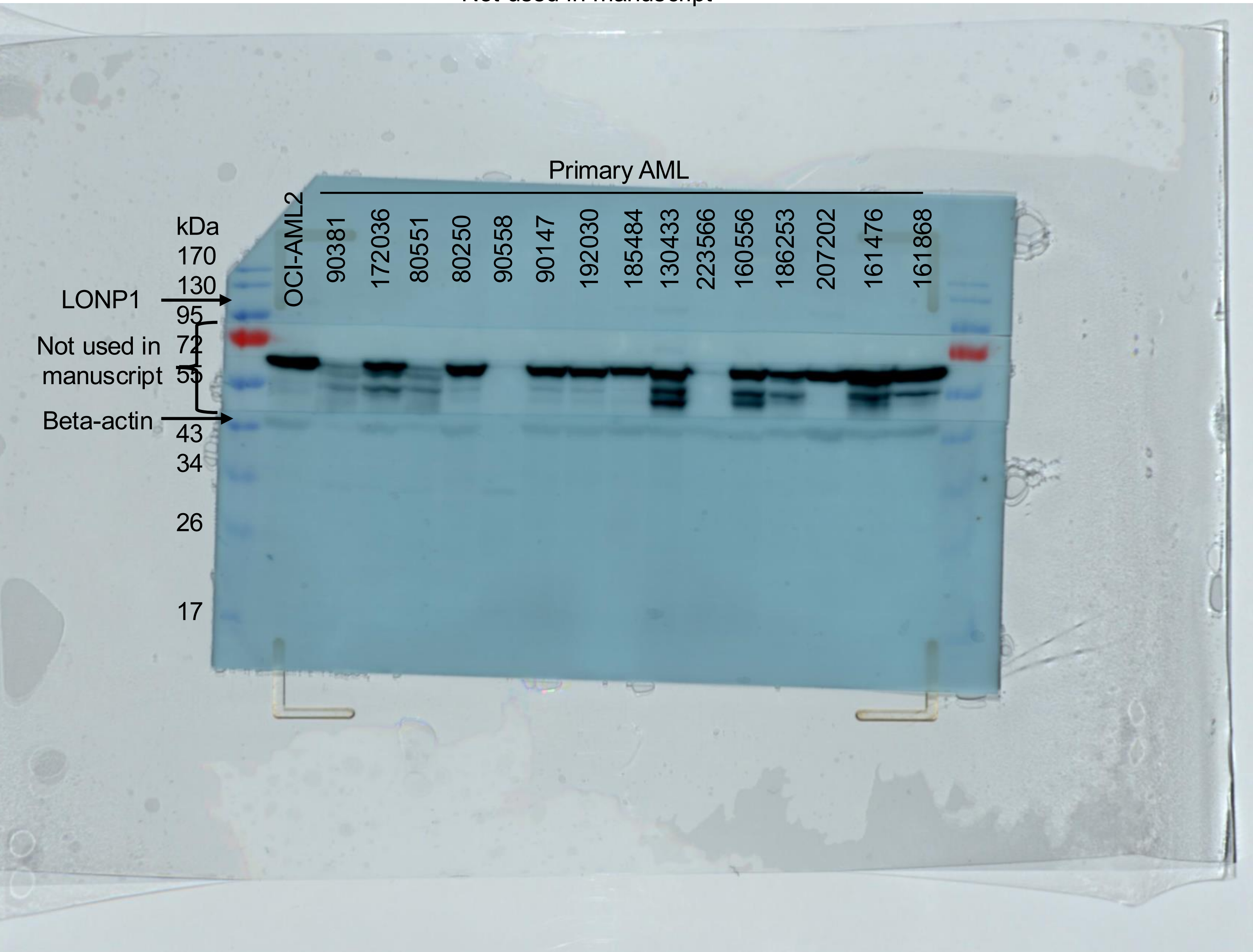

Unused fragment removed

Re-imaged blot used in manus

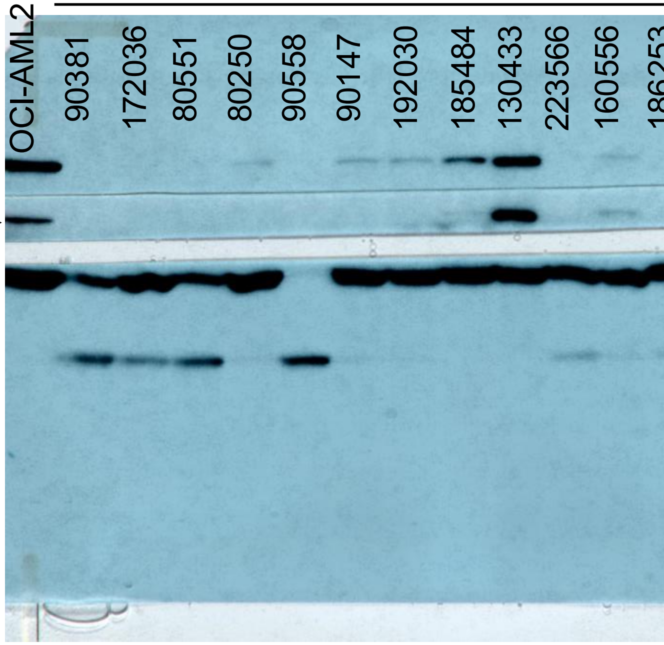

Figure S5B

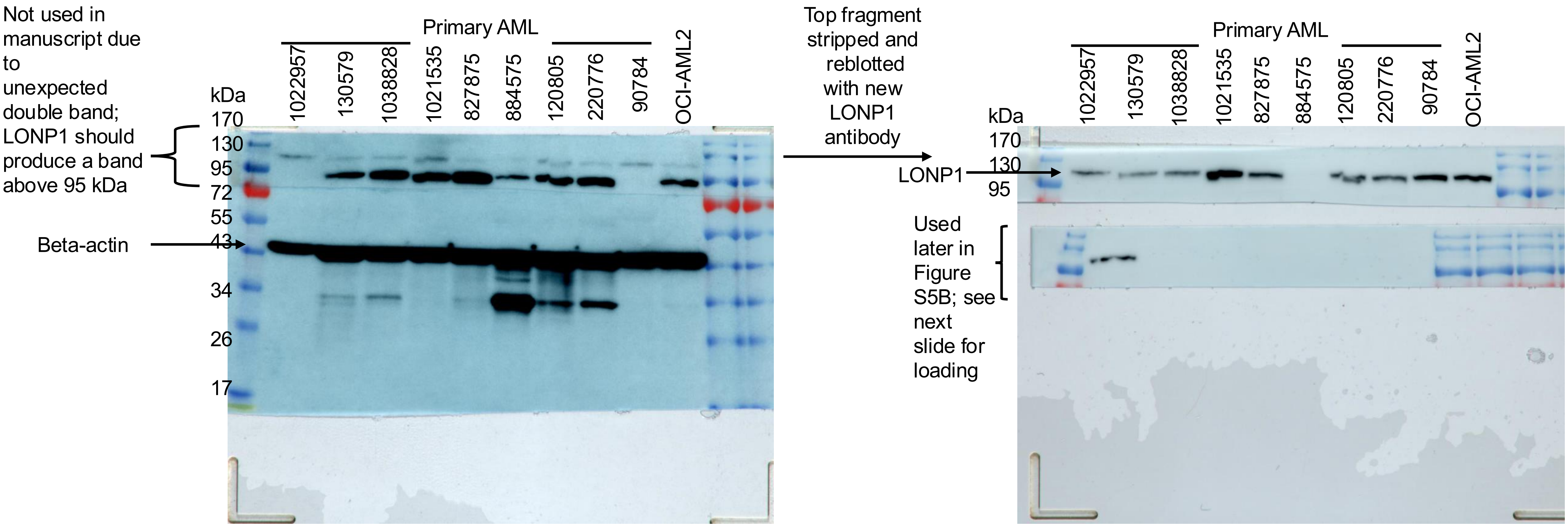

Not used in manuscript due to unexpected double band; LONP1 should produce a band above 95 kDa
Primary AML
1022957
130579
1038828
1021535
827875
884575
120805
220776
90784
OCI-AML2
kDa
170
130
95
72
55
43
34
26
17
Beta-actin
Top fragment stripped and reblotted with new LONP1 antibody
LONP1
Used later in Figure S5B; see next slide for loading

Figure S5B

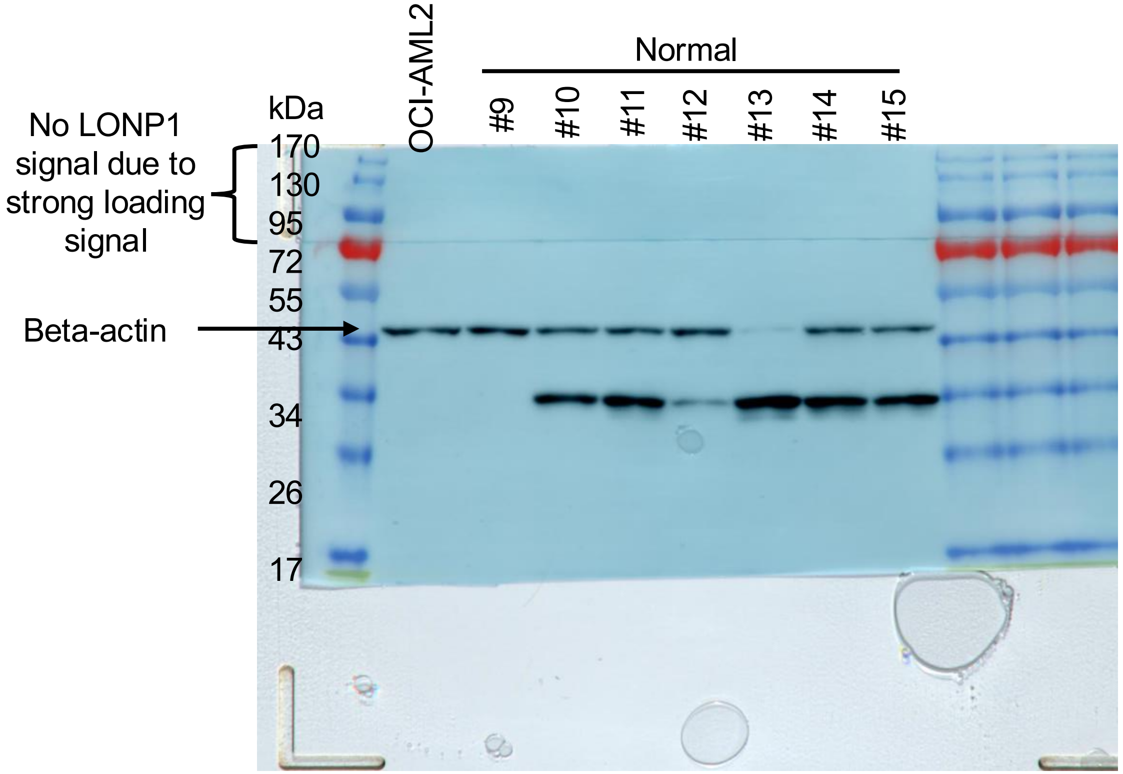

Fragment re-imaged alone without beta-actin fragment

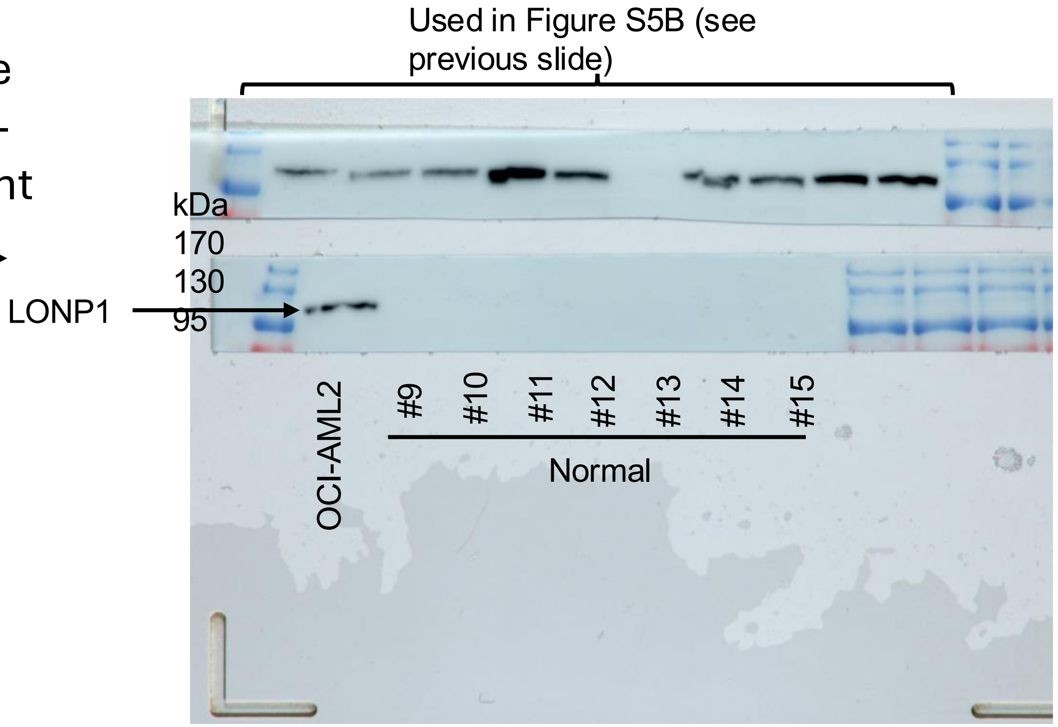

Figure S9A

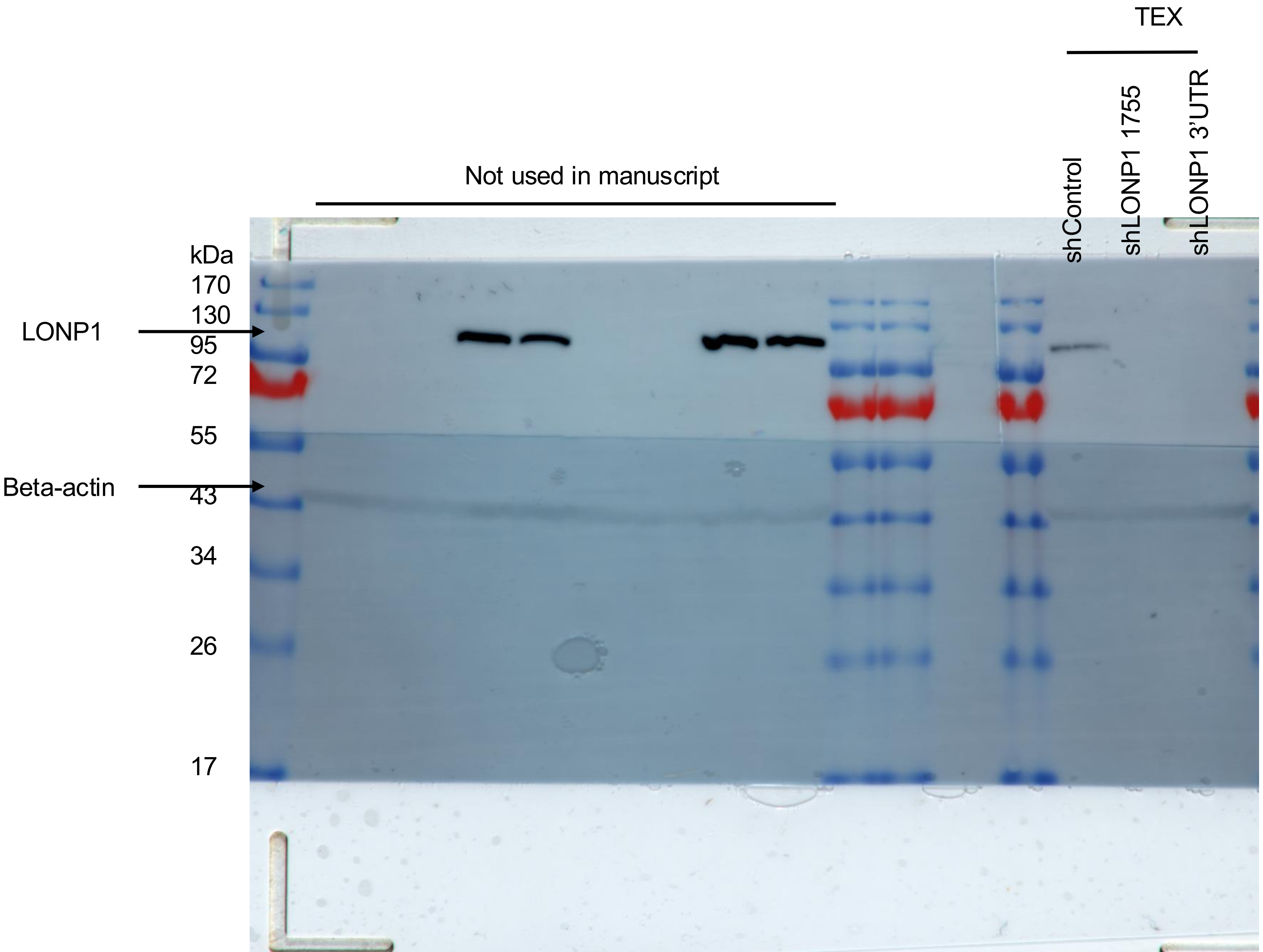
TEX
Not used in manuscript
shControl
shLONP1 1755
shLONP1 3'UTR
kDa
170
130
95
72
55
43
34
26
17
LONP1
Beta-actin

Figure S9C

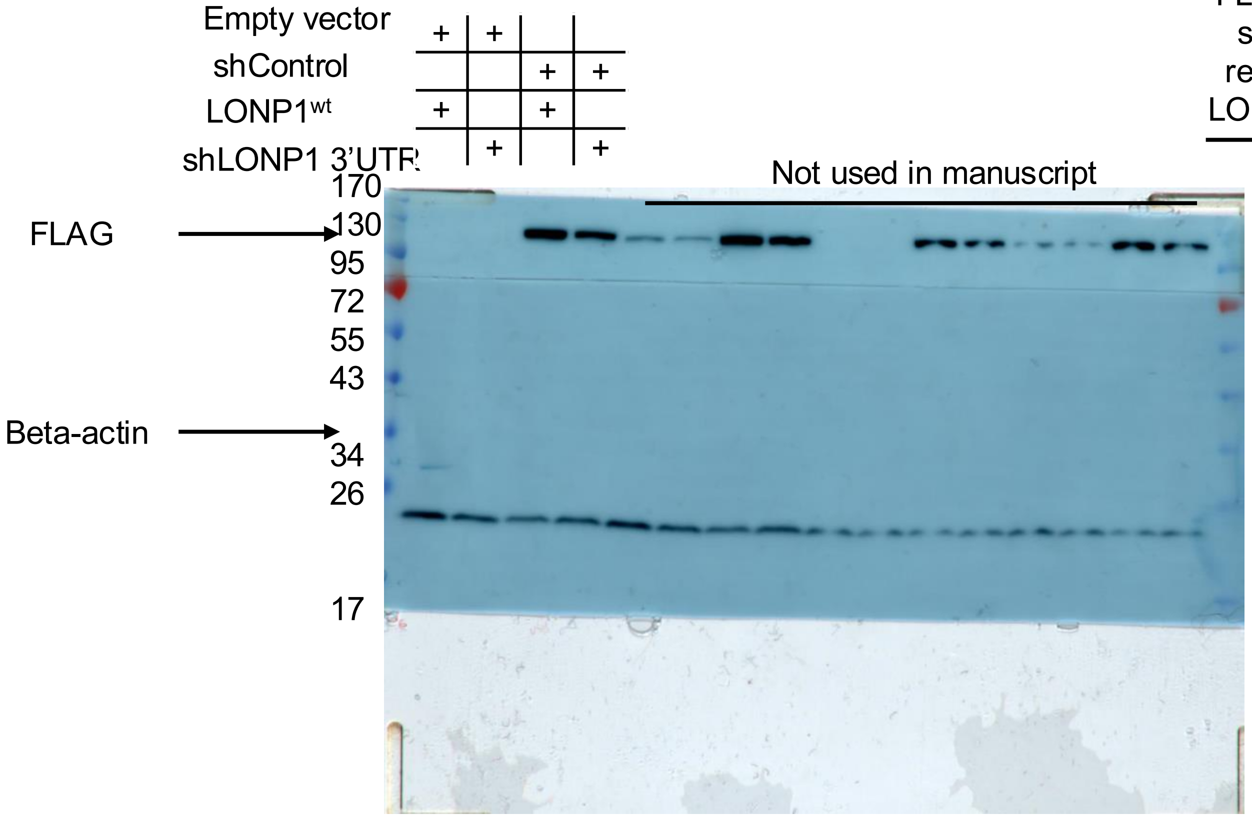

FLAG fragment stripped and re-blotted with LONP1 antibody

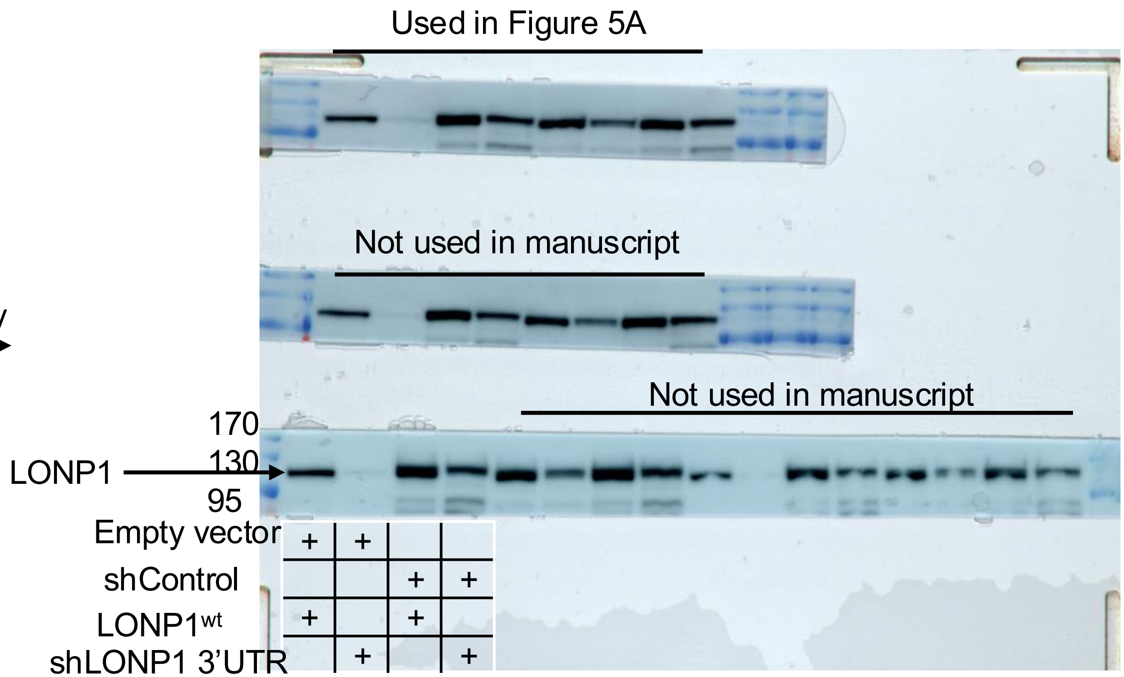

Figure S16

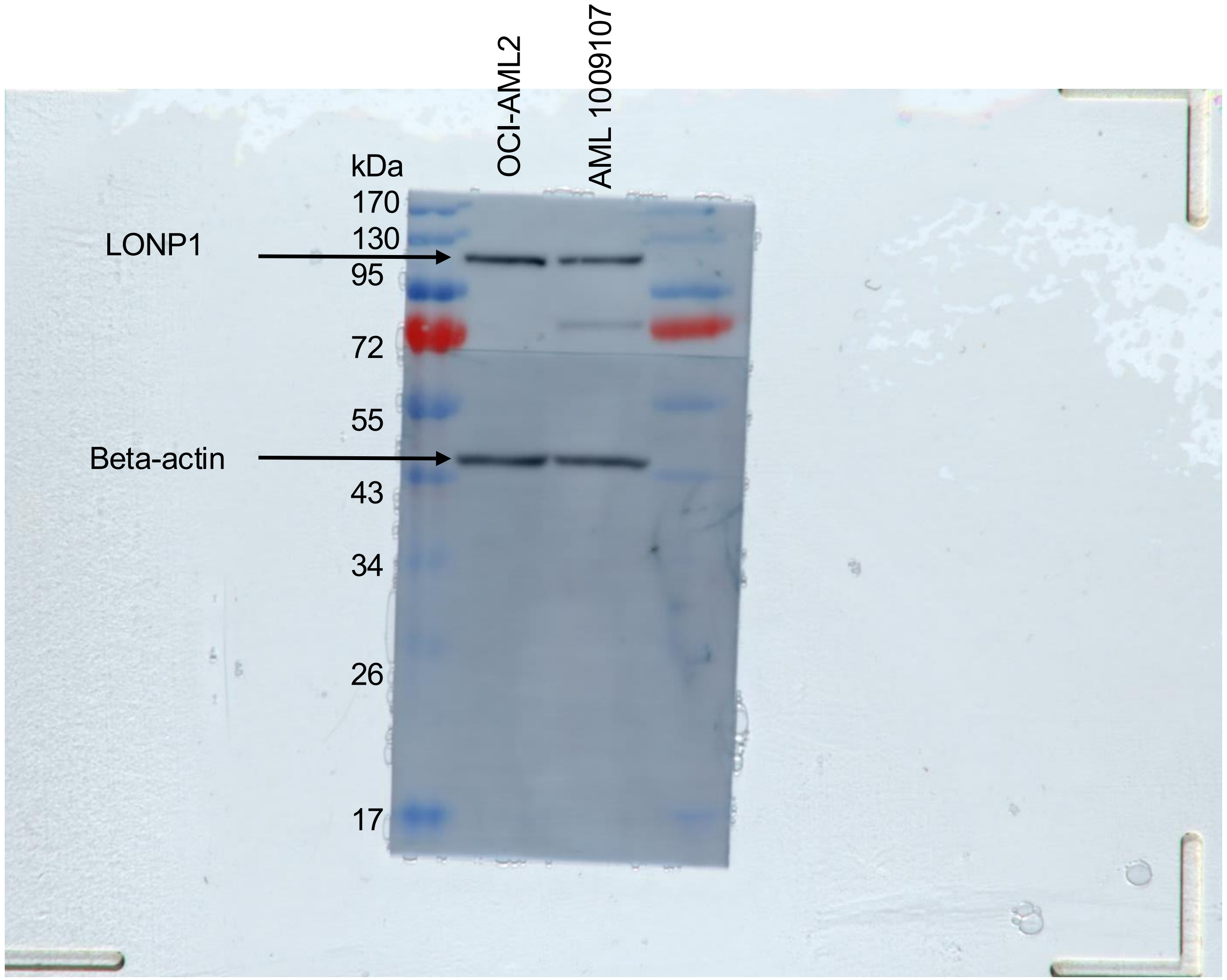

OCI-AML2
AML 1009107
kDa
170
130
95
72
55
43
34
26
17
LONP1
Beta-actin

Figure S16

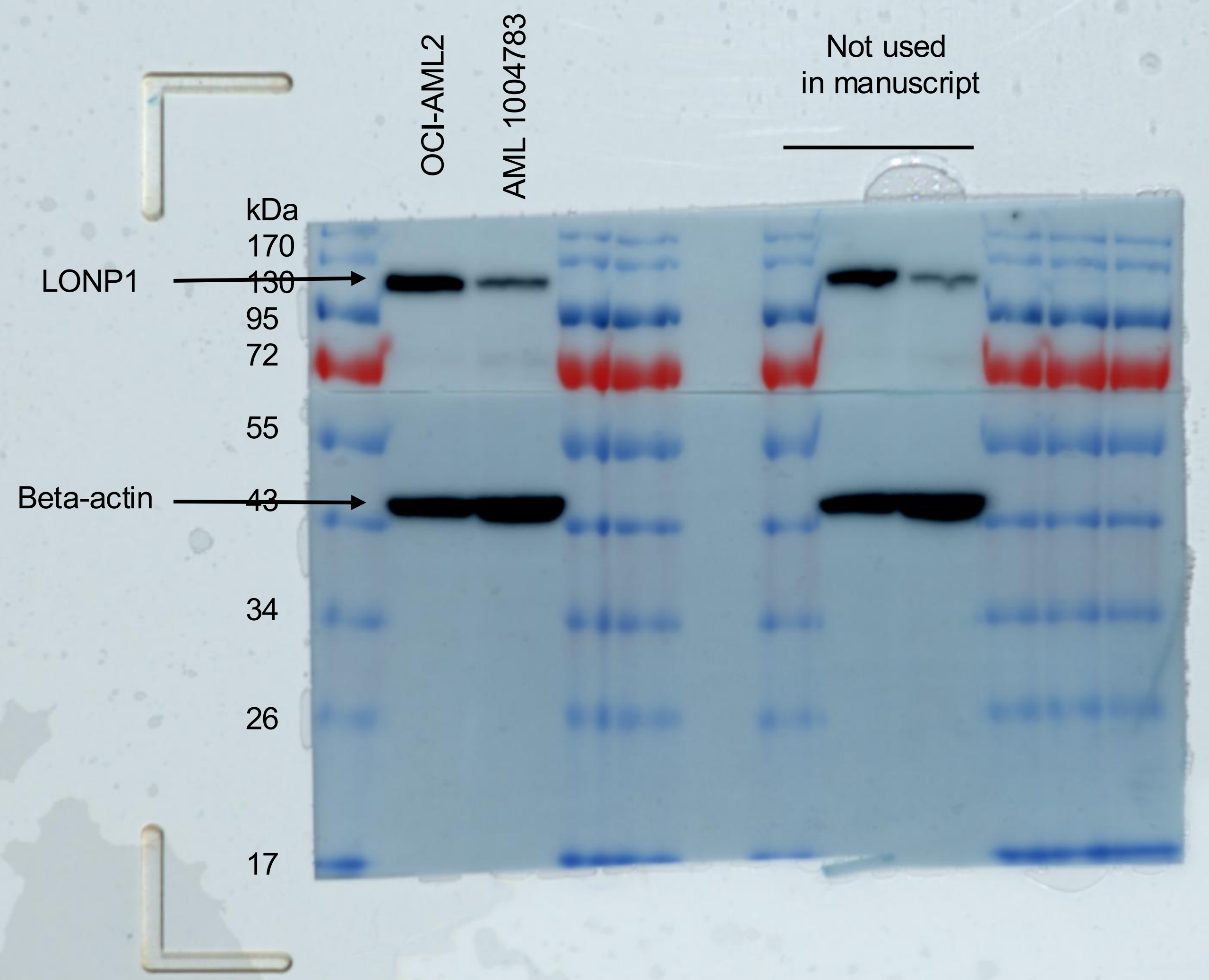
OCI-AML2
AML 1004783
Not used
in manuscript
kDa
170
130
95
72
55
43
34
26
17
LONP1
Beta-actin

Figure S16

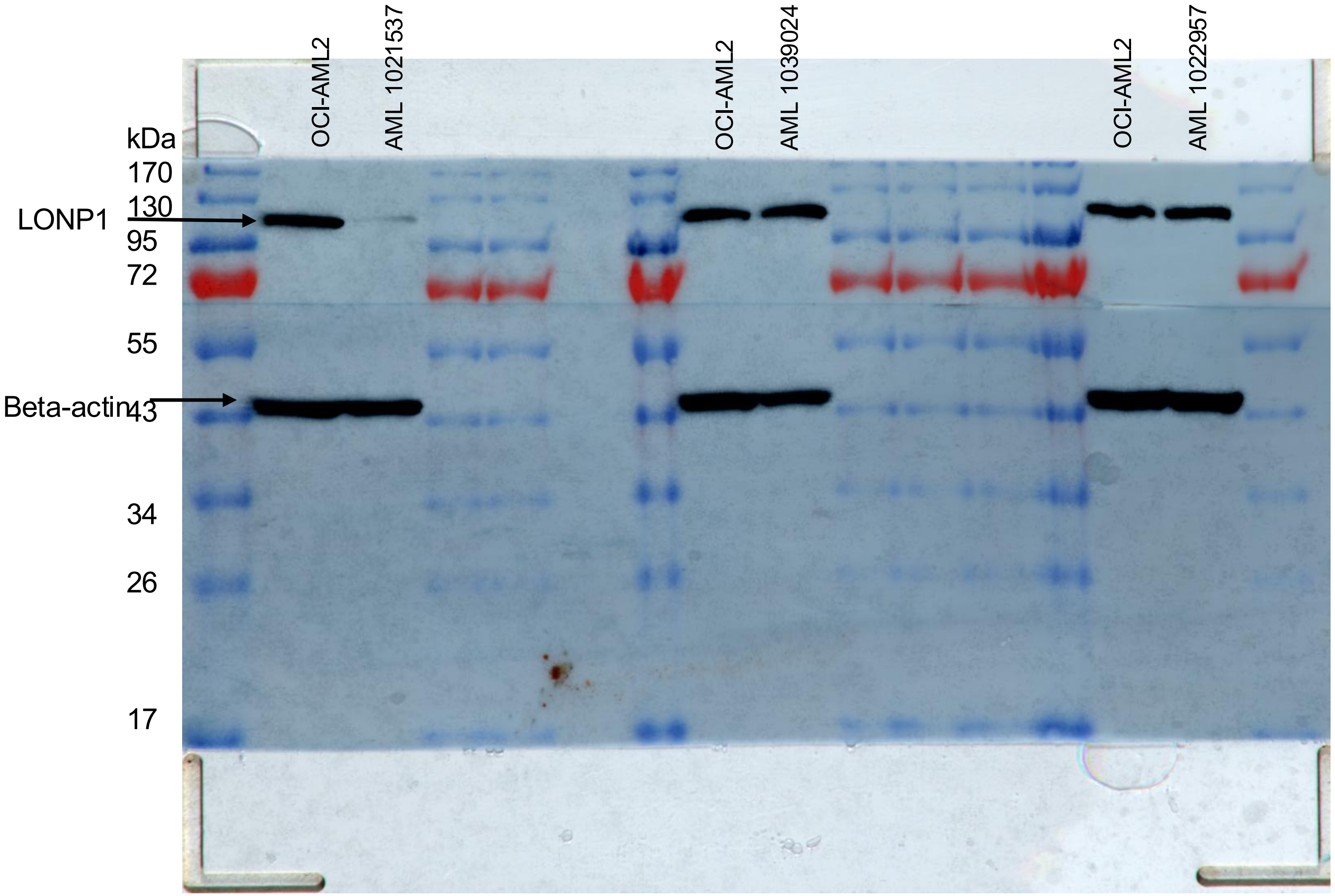
OCI-AML2
AML 1021537
OCI-AML2
AML 1039024
OCI-AML2
AML 1022957
kDa
170
130
95
72
55
43
34
26
17
LONP1
Beta-actin

Figure S16

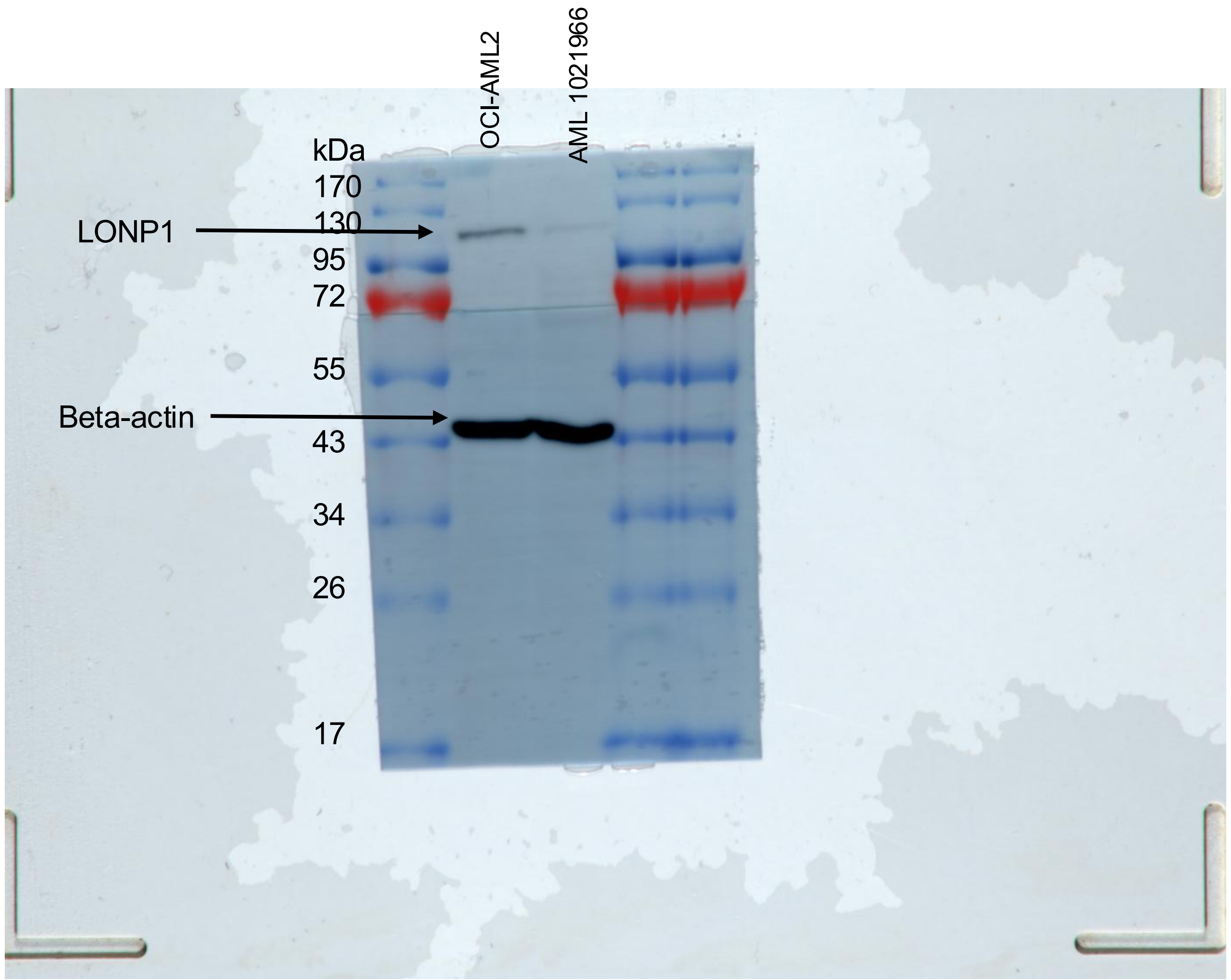
OCI-AML2
AML 1021966
kDa
170
130
95
72
55
43
34
26
17
LONP1
Beta-actin

Figure S16

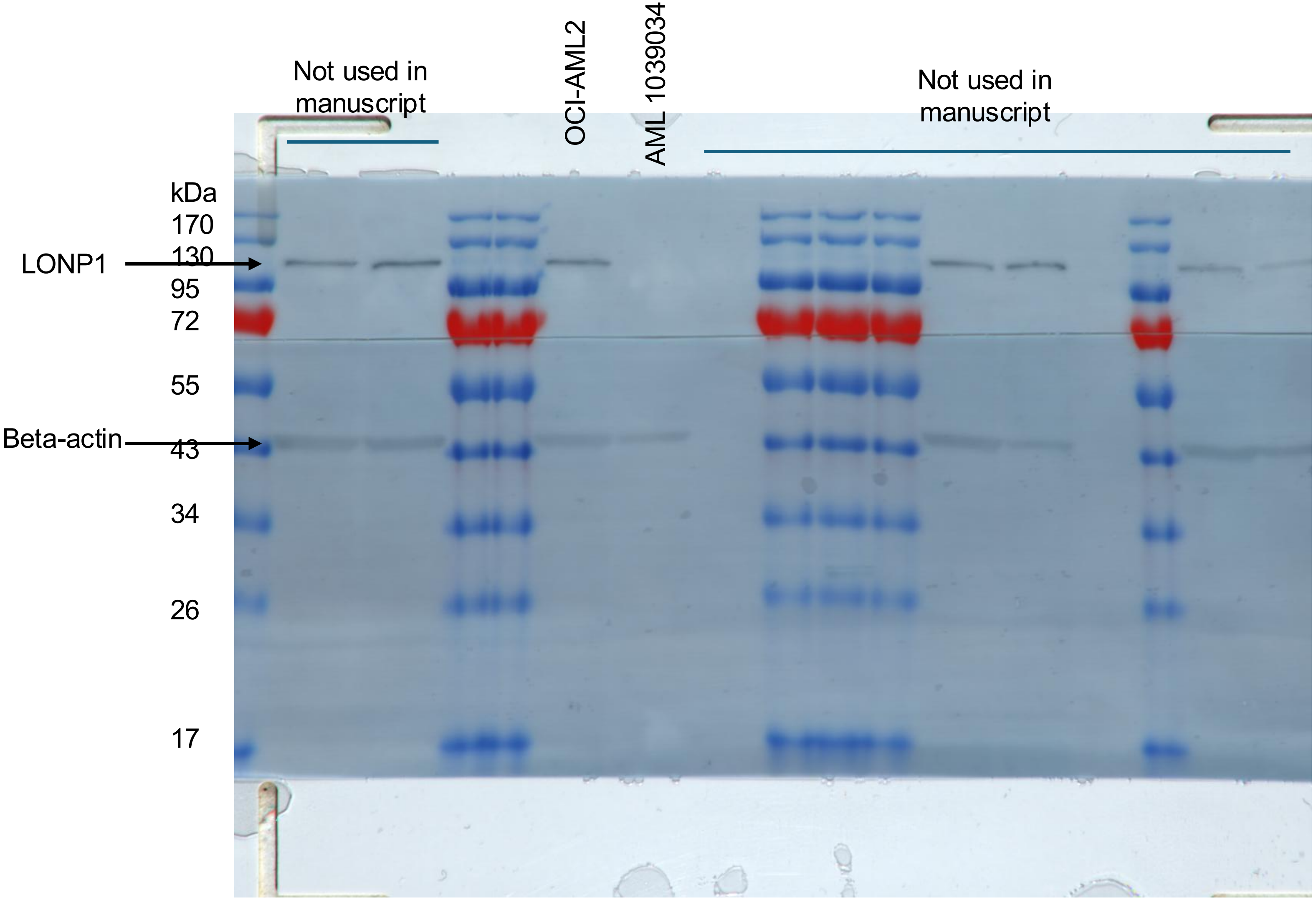
Not used in manuscript
OCI-AML2
AML 1039034
Not used in manuscript
kDa
170
130
95
72
55
43
34
26
17
LONP1
Beta-actin

Figure S18

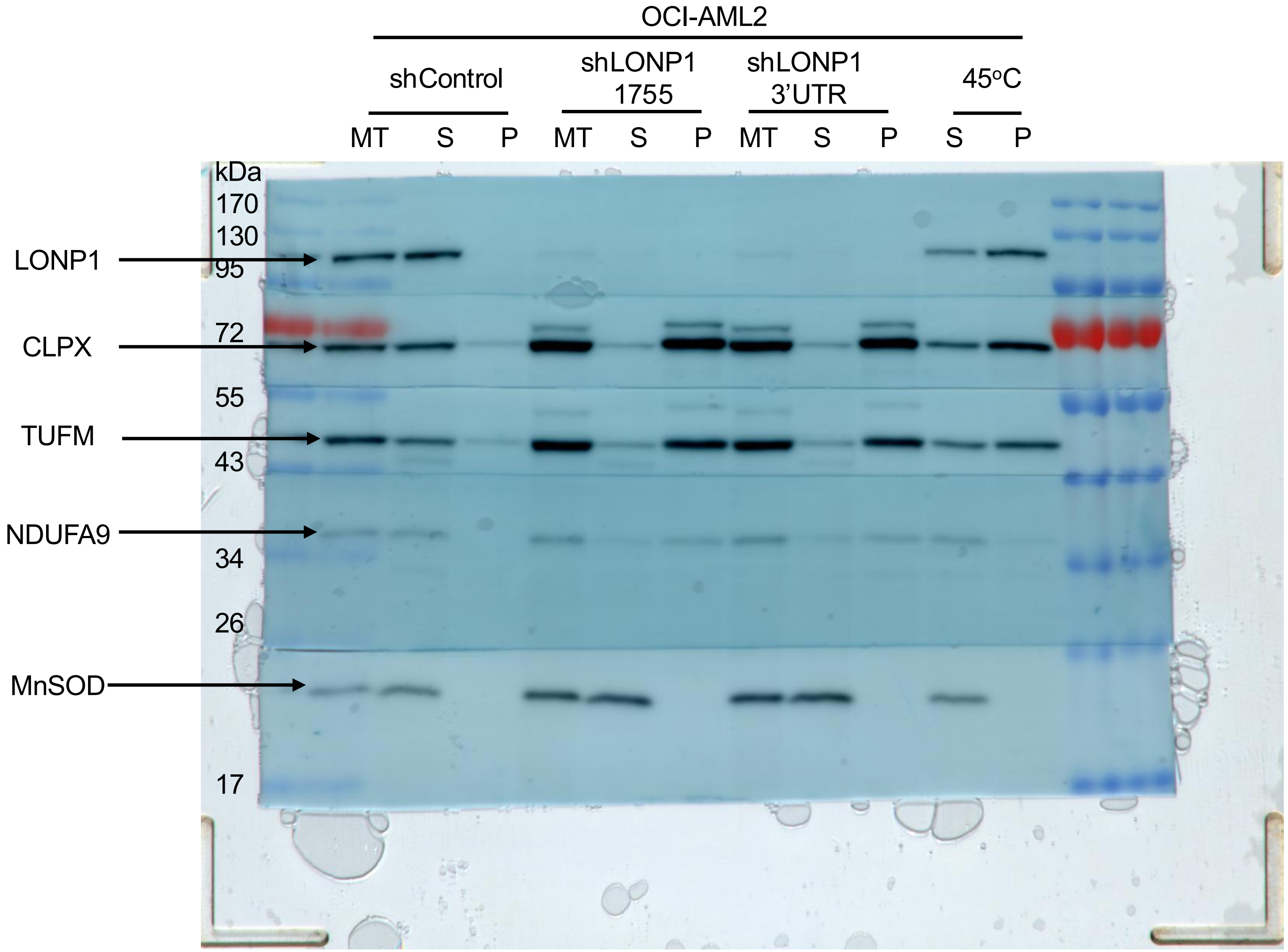
OCI-AML2
shControl
shLONP1 1755
shLONP1 3'UTR
45°C
MT S P MT S P MT S P S P
kDa
170
130
95
72
55
43
34
26
17
LONP1
CLPX
TUFM
NDUFA9
MnSOD

Figure S19A

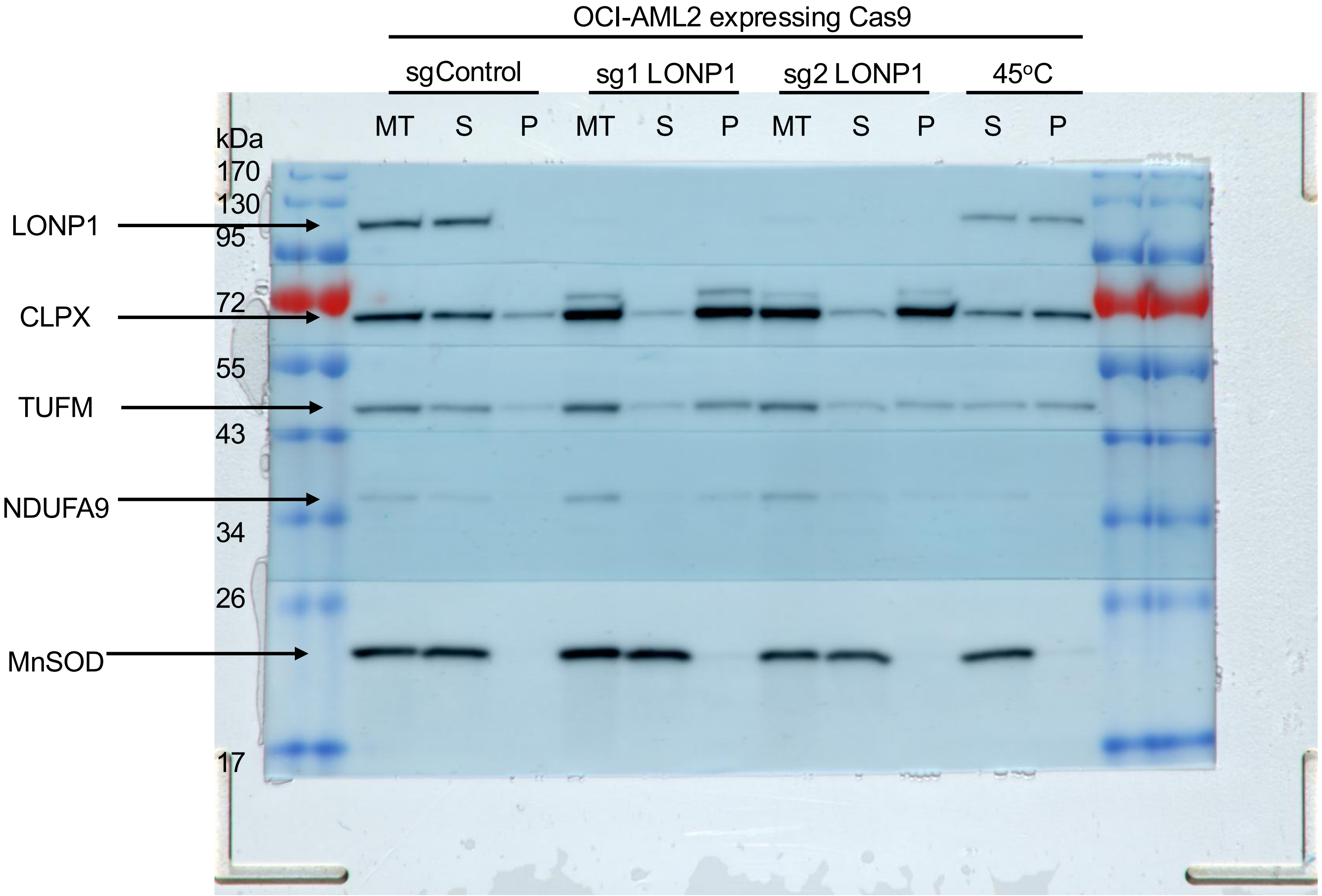
OCI-AML2 expressing Cas9
sgControl
sg1 LONP1
sg2 LONP1
45oC
MT S P MT S P MT S P S P
kDa
170
130
95
72
55
43
34
26
17
LONP1
CLPX
TUFM
NDUFA9
MnSOD

Figure S19A

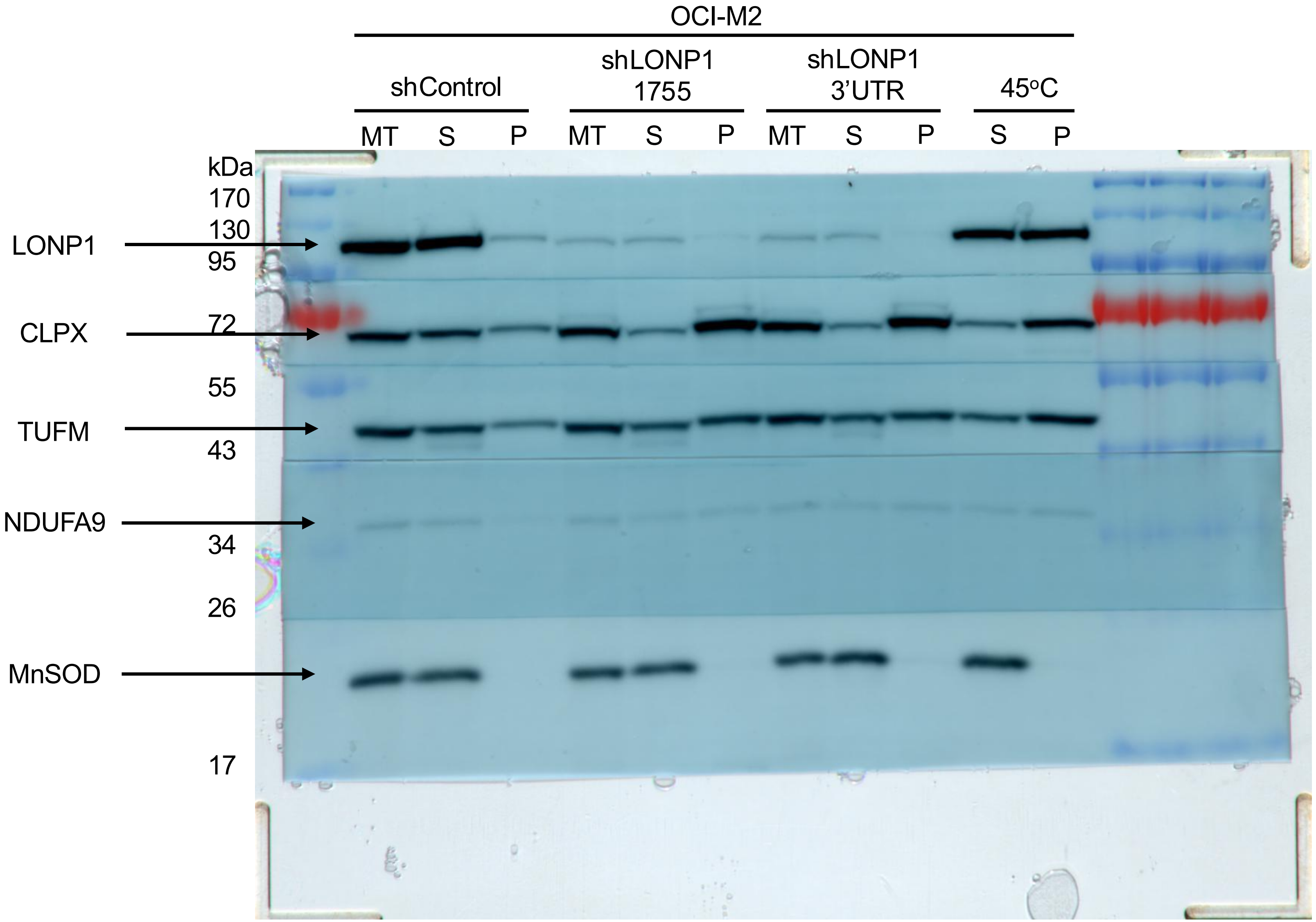

OCI-M2
shControl
shLONP1 1755
shLONP1 3'UTR
45°C
MT S P MT S P MT S P S P
kDa
170
130
95
72
55
43
34
26
17
LONP1
CLPX
TUFM
NDUFA9
MnSOD

Figure S19A

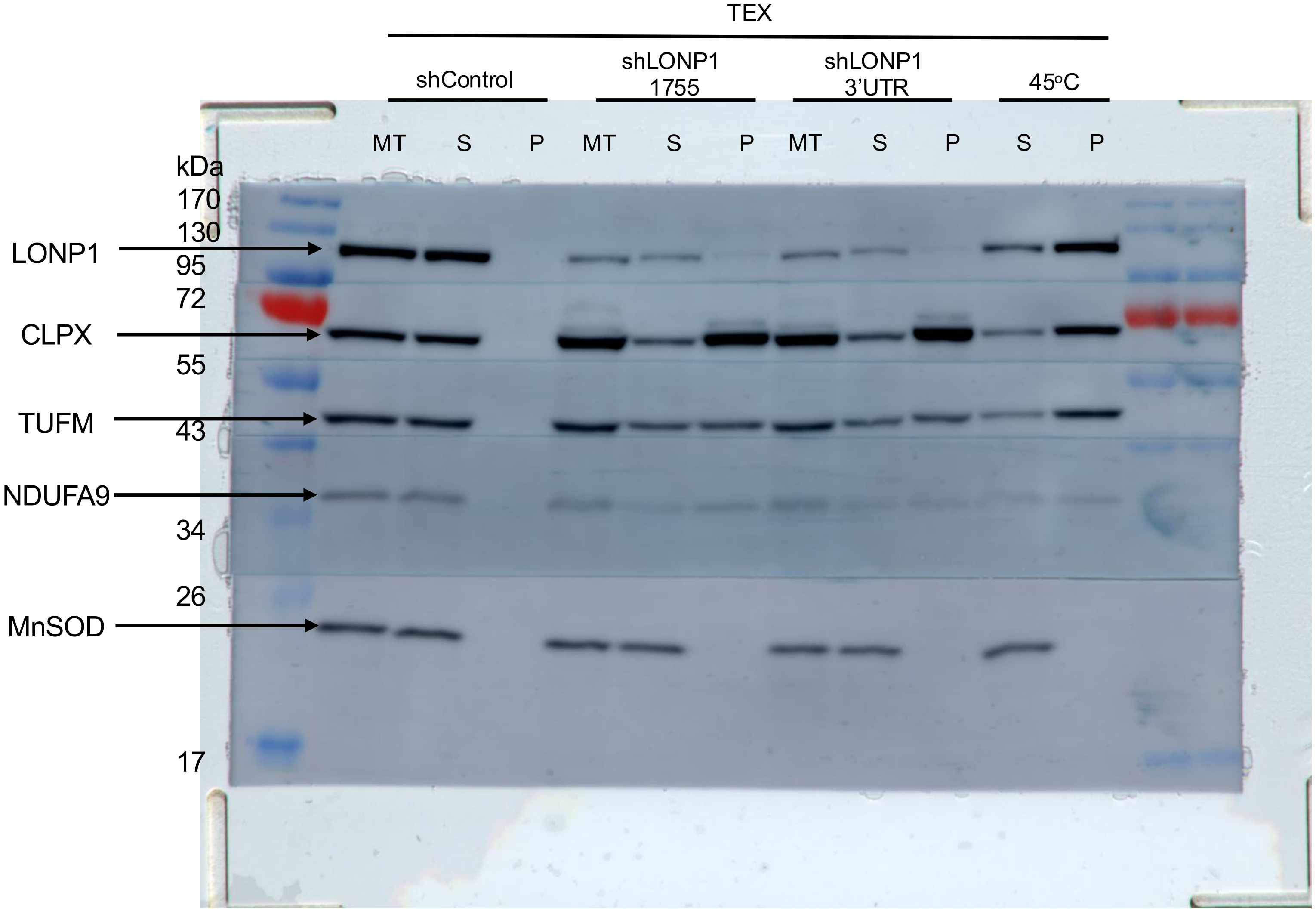
TEX
shControl
shLONP1 1755
shLONP1 3'UTR
45oC
MT S P MT S P MT S P S P
kDa
170
130
95
72
55
43
34
26
17
LONP1
CLPX
TUFM
NDUFA9
MnSOD

Figure S19A

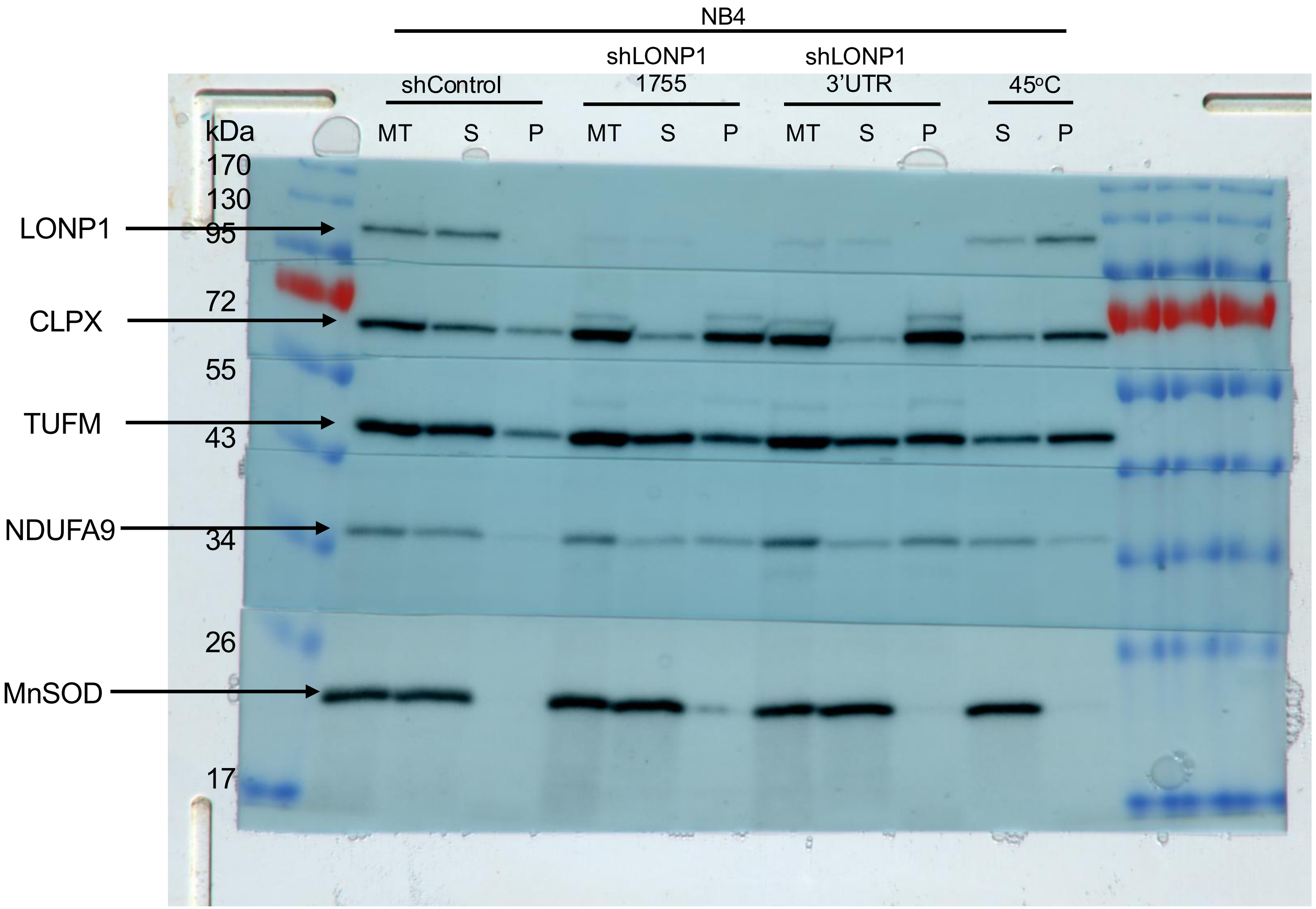

NB4
shControl
shLONP1 1755
shLONP1 3'UTR
45°C
MT S P MT S P MT S P S P
kDa
170
130
95
72
55
43
34
26
17
LONP1
CLPX
TUFM
NDUFA9
MnSOD

Figure S21

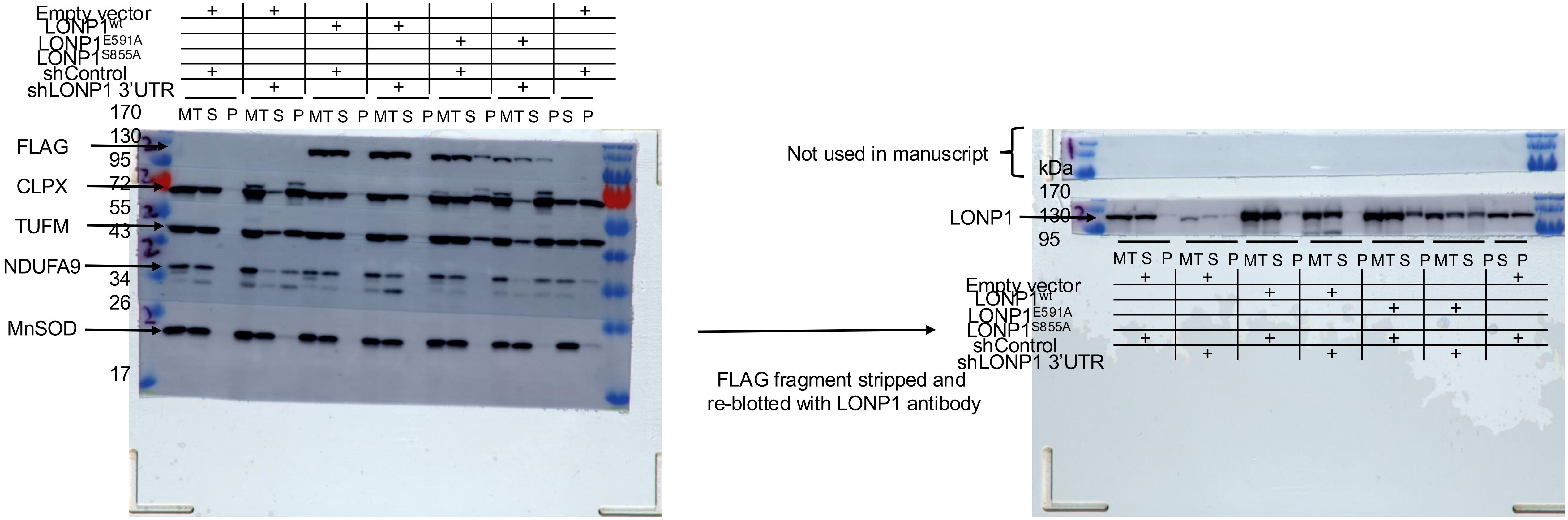

Empty vector
LONP1wt
LONP1E591A
LONP1S855A
shControl
shLONP1 3'UTR
170
130
95
72
55
43
34
26
17
MT S P MT S P MT S P MT S P MT S P MT S P S P
FLAG
CLPX
TUFM
NDUFA9
MnSOD
FLAG fragment stripped and re-blotted with LONP1 antibody
Not used in manuscript
kDa
LONP1

Fig S21

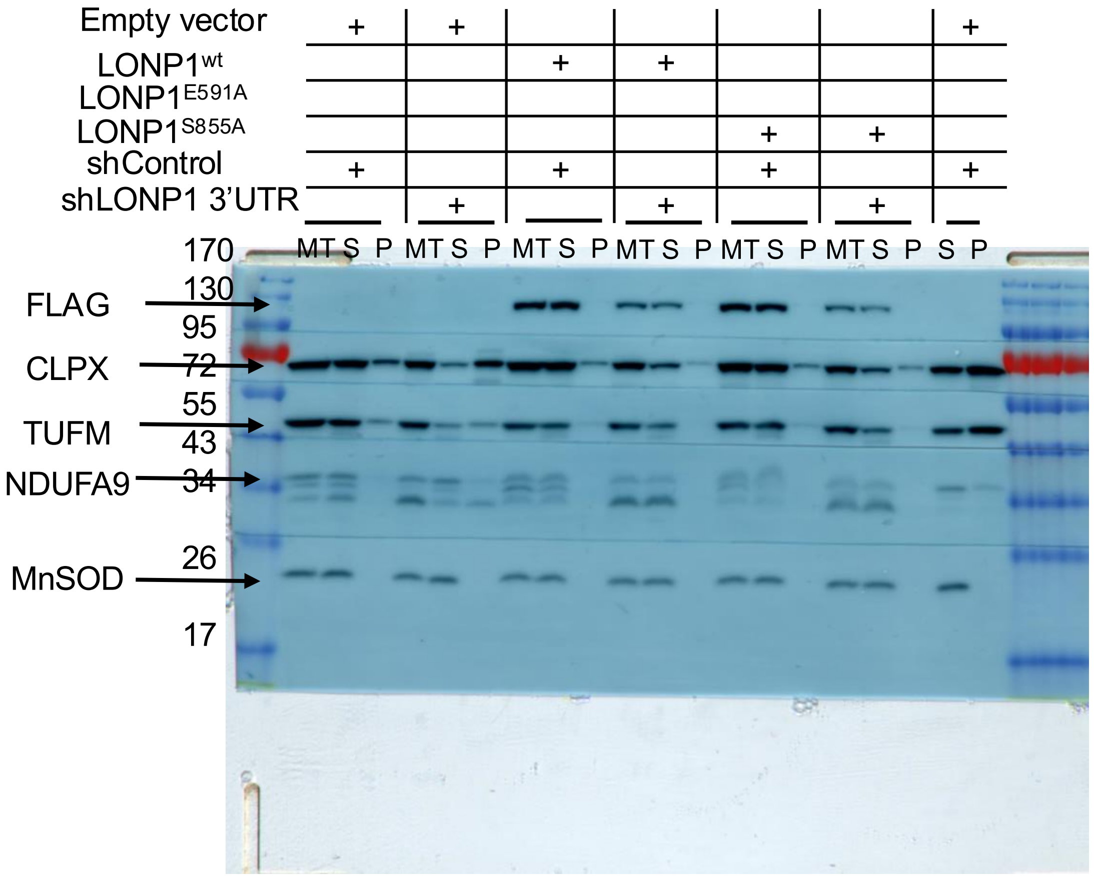

FLAG fragment stripped and re-blotted with LONP1 antibody

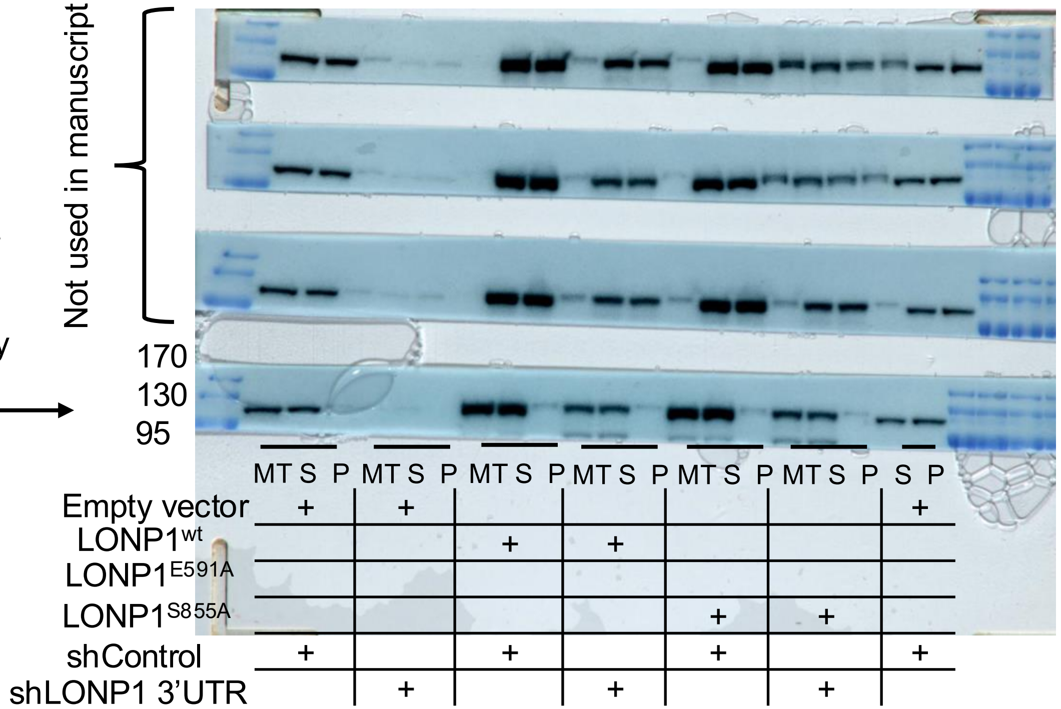

Supplement: Unedited blot and gel images [file jci-136-196687-s086.pdf]
